# Calamene-Type Sesqui-, Mero-, and Bis-sesquiterpenoids from Cultures of *Heimiomyces* sp., a Basidiomycete Collected in Africa

**DOI:** 10.1021/acs.jnatprod.2c01015

**Published:** 2023-02-13

**Authors:** Sebastian Pfütze, Atchara Khamsim, Frank Surup, Cony Decock, Josphat C. Matasyoh, Marc Stadler

**Affiliations:** † Department of Microbial Drugs, Helmholtz Centre for Infection Research GmbH (HZI) and German Centre for Infection Research (DZIF), Partner Site Hannover-Braunschweig, Inhoffenstraße 7, 38124 Braunschweig, Germany; ⊥ Institute of Microbiology, Technische Universität Braunschweig, Spielmannstraße 7, 38106 Braunschweig, Germany; ‡ Mycothèque de l’Université Catholique de Louvain (BCCM/MUCL), Earth and Life Institute, Microbiology, B-1348 Louvain-la-Neuve, Belgium; § Department of Chemistry, Egerton University, P.O. Box 536, Njoro 20115, Kenya

## Abstract

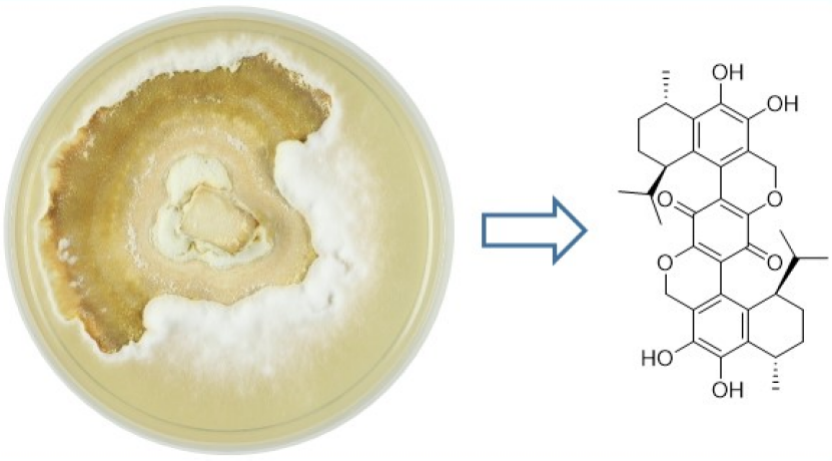

New meroterpenoids bis-heimiomycins A–D (**1**–**4**) and heimiomycins D and E (**5** and **6**) were isolated from solid rice cultures of *Heimiomyces* sp., while new calamene-type sesquiterpenoids heimiocalamene A (**7**) and B (**8**) were isolated from shake cultures, respectively. Structures of the metabolites were elucidated by 1D and 2D NMR in addition to HRESIMS data. While relative configurations were assigned by ROESY data, absolute configurations were derived from the structurally related, previously described calamenes, which we herein name heimiocalamenes C–E (**9**–**11**). A plausible biosynthetic pathway was proposed for **1**–**6**, with a radical reaction connecting their central *para*-benzoquinone building block to calamene-sesquiterpenoids. Based on the assumption of a common biosynthesis, we reviewed the structure of the known nitrogen-containing derivative **11**, calling the validity of the originally proposed structure into question. Subsequently, the structure of **11** was revised by analysis of HMBC and ROESY NMR data. Only heimiomycin D (**5**) displayed cytotoxic effects against cell line KB3.1.

Basidiomycota represent the second largest phylum in the kingdom of fungi and are well known to show both a high biological diversity by comprising more than 35,000 species^1^ and a high chemical diversity of their secondary metabolites, leading to the identification of many different classes of natural products and especially important bioactive molecules like strobilurins and pleuromutilins.^2^ In particular, these species can be found in partly untapped ecosystems that have not been exhaustively explored yet. For this reason, *Heimiomyces* sp. (MUCL 56078, collected from Mount Elgon National Reserve, Kenya) was evaluated for its secondary metabolite profile, and previous studies led to the identification of several new secondary metabolites (**9**–**14**).^3^ In the course of this work, the presence of a vast amount of secondary metabolites was observed within the extracts, which led to further studies on this strain. Herein, we present the isolation, structure elucidation, and biological evaluation of new meroterpenoids bis-heimiomycins A–D and heimiomycins D and E (**1**–**6**) from solid rice cultures, as well as the new calamene-type sesquiterpenoids heimiocalamenes A (**7**) and B (**8**) from shaking cultures of *Heimiomyces* sp. Furthermore, known compounds heimiomycin B (**13**), hispidin (**15**), and hypholomin B (**16**) (Figures 2 and S4) were isolated from both liquid and solid cultures. Heimiomycin B (**13**) was recently published together with heimiomycins A and C, as well as three new calamene derivatives (Figure 2), which we propose to name heimiocalamenes C–E (**9**–**11**), after isolation from liquid cultures of *Heimiomyces* sp.,^3^ emphasizing that this species shows a very diverse secondary metabolite profile.

**Figure 1:**
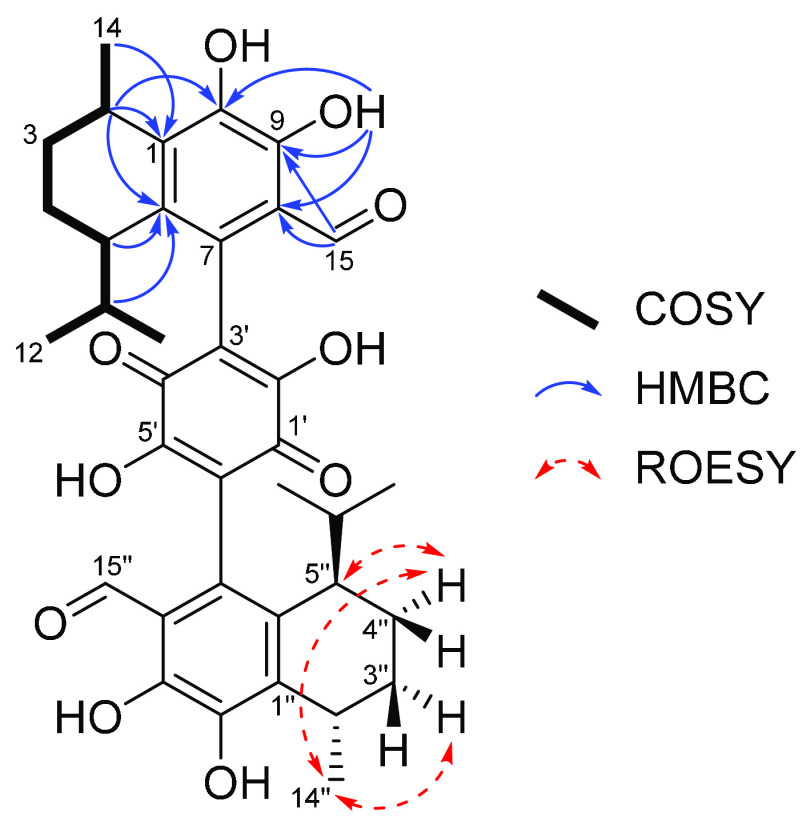
Key COSY, HMBC, and ROESY correlations of bis-heimiomycin A (**1**).

**Figure 2:**
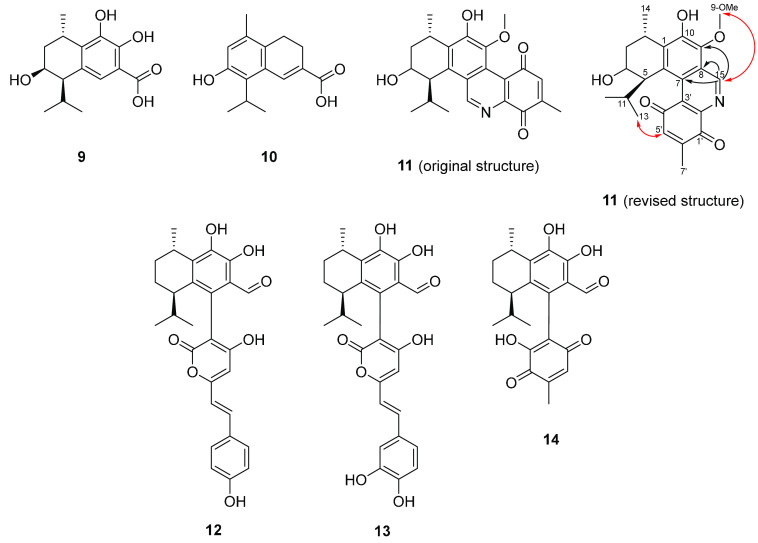
Compounds previously isolated from *Heimiomyces* sp. **9**–**11**: heimiocalamenes C–E (including originally proposed and revised structure of **11** with key HMBC (black arrows) and ROESY (red arrows) correlations); **12**–**14**: heimiomycins A–C.

## Results and Discussion

Bis-heimiomycin A (**1**) was isolated as a red oil from extracts of solid rice cultures. Its HRESIMS data indicated the molecular formula C_36_H_40_O_10_ according to the molecular ion cluster at *m*/*z* 633.2695 [M + H]^+^, indicating 17 degrees of unsaturation. Based on the molecular formula together with the carbon and proton resonances a symmetry within the molecule was assumed: ^1^H NMR (Table 1) and HSQC data led to the identification of three methyls (H_3_-12/12″, H_3_-13/13″, H_3_-14/14″), four methylenes (H-3α/3″α, H-3β/3″β, H-4α/4″α, H-4β/4″β), three methines (H-2β/2″β, H-5α/5″α, H-11/11″), and one aldehyde function (H-15/15″). The ^13^C (Table 1) and HMBC NMR data revealed the presence of 16 carbon resonances, comprising one carbonyl carbon (C-15/15″), seven nonprotonated sp^2^-hybridized carbons (C-1/1″, C-2′/5′, C-6/6″, C-7/7″, C-8/8″, C-9/9″, C-10/10″), three methines (C-2/2″, C-5/5″, C-11/11″), two methylene carbons (C-3/3″, C-4/4″), and three methyl carbons (C-12/12″, C-14/14″, C-13/13″). By analyzing the COSY data a spin system between H_3_-14, H-2, H_2_-3, H_2_-4, H-5, H-11, and H_3_-12/H_3_-13 was constructed. HMBC correlations from H_3_-14 to C-1/C-2/C-3, H-11 to C-4/C-5/C-6/C-12/C-13, and H_3_-12/H_3_-13 to C-5/C-11 revealed a 5-isopropyl-2-methylcyclohex-1-ene substructure. Further HMBC correlations from H-2 to C-1/C-10, H-5 to C-6, 9-OH to C-8/C-9/C-10, and H-15 to C-8/C-9/C-10 led to the identification of a dihydroxybenzaldehyde substructure that was fused to the 5-isopropyl-2-methylcyclohex-1-ene substructure across C-1 and C-6. The carbonyl and sp^2^-hybridized carbons, as well as the two rings of the calamene-type substructure, accounted for 7 degrees of unsaturation, leaving 10 degrees of unsaturation to be assigned within the scaffold of bis-heimiomycin A (**1**). This led to the assumption of two calamene-type substructures being linked by a central dihydroxy-quinone moiety. Nevertheless, of the potential dihydroxy-quinone moiety only C-2′/C-5′ could be observed in the ^13^C spectrum, whereas signals for carbons C-1′/C-3′/C-4′/C-6′ were missing. To prevent rapid tautomerism, which was assumed to be the reason for the missing resonances, bis-heimiomycin A (**1**) was reacted with acetic anhydride in pyridine and positions C-15/C-15″, 2′OH/5′OH, 9OH/9″OH, and 10OH/10″OH were derivatized. Based on 1D and 2D NMR data (Table S5) of the resulting product **1b**, peracetylation and a ring closure at C-15/C-15″ of **1** were confirmed due to the appearance of six additional methyls (H_3_-AcC2-9/9″, H_3_-AcC2-10/10″, H_3_-AcC2-15/15″), six carbonyl carbons (AcC1-9/9″, AcC1-10/10″, AcC1-15/15″), and two oxymethines (H-15/15″). Key HMBC correlations from H-15 to C-2′/C-8/C-9/AcC1-15 and H-15″ to C-5′/C-8″/C-9″/AcC1-15″, as well as carbons C-1′/C-2′/C-3′/C-4′/C-5′/C-6′, could be observed in the HMBC and ^13^C spectra, leading to a confirmation of the structure previously assumed for **1**. The relative and absolute configurations were assigned as 2*S*,2′*S*,5*R*,5′*R* by comparison of ^13^C and ROESY data, as well as ECD spectra (Figure S1), to related calamene-type derivatives described by Cheng et al.,^3^ which were previously isolated from the same fungal strain (*Heimiomyces* sp. MUCL 56078) and showed similar correlations.

**Table 1:** ^13^C and ^1^H NMR Spectroscopic Data of Compounds **1**–**4** in Acetone-*d*_6_ (δ in ppm)

	**1**^b^^,^^c^		**2**^b^^,^^d^		**3**^b^^,^^d^		**4**^a^^,^^d^	
no.	δ_C_, type	δ_H_ (*J* in Hz)	δ_C_, type	δ_H_ (*J* in Hz)	δ_C_^g^, type	δ_H_ (*J* in Hz)	δ_C_, type	δ_H_ (*J* in Hz)
1	137.9, C		139.0, C		128.9, C		131.4, C	
2	27.8, CH	β: 3.42, m	28.8, CH	β: 3.41, m	27.5, CH	3.28, m	28.4, CH	β: 3.35, m
3	25.1, CH_2_	α: 1.49, br d (13.6)	26.1, CH_2_	α: 1.48, m	26.3, CH_2_	1.45, m	26.8, CH_2_	α:1.49, m
		β: 2.15, m		β: 2.09, m		2.08, s		β: 1.99, m
4	19.6, CH_2_	α: 1.82, m	20.4, CH_2_	α: 1.79, m	19.9, CH_2_	1.77, m	19.7, CH_2_	α: 1.70, m
		β: 1.89, br s		β: 2.00, m		1.99, m		β: 1.96, m
5	40.8, CH	α: 2.48, br s	42.0, CH	α: 2.35, br s	41.2, CH	2.74, m	40.3, CH	α: 2.81, m
6	132.6, C		133.6, C		124.6, C		132.5, C	
7	132.6, C		124.2, C		118.4, C		117.8, C	
8	115.9, C		116.4, C		122.2, C		120.3, C	
9	147.8, C		148.8, C		142.2, C		145.8, C	
9OH		11.94		11.90				
10	142.7, C		143.5, C		143.7, C		138.2, C	
11	33.1, CH	1.75, m	33.6, CH	1.85, m	34.0, CH	1.63, m	34.6, CH	1.70, m
12	21.6, CH_3_^e^	0.82, d (6.9)	22.2, CH_3_	0.84, d (6.9)	21.8, CH_3_	0.65, d (6.9)	22.0, CH_3_	0.64, d (6.7)
13	20.0, CH_3_	0.79, d (6.9)	21.1, CH_3_	0.78, d (6.9)	20.1, CH_3_	0.68, d (6.9)	20.0, CH_3_	0.61, d (6.7)
14	21.6, CH_3_^e^	1.23, d (6.9)	22.6, CH_3_	1.24, d (6.9)	22.6, CH_3_	1.21, d (6.9)	22.7, CH_3_	1.21, d (7.0)
15	198.8, CH	9.88, s	199.5, CH	9.79, s	62.5, CH_2_	4.55, d (13.8)	67.0, CH_2_	4.68, d (13.0)
						4.82, d (13.8)		5.64, d (13.0)
1′	f	f	180.9, C		f	f	178.5, C	
2′	147.5, C		162.4, C		f	f	154.2, C	
3′	f	f	114.4, C		f	f	125.1, C	
4′	f	f	180.7, C		f	f	178.5, C	
5′	147.5, C		157.6, C		157.0, C		154.2, C	
6′	f	f	114.4, C		117.2, C		125.1, C	
1″	137.9, C		131.4, C		131.0, C		131.4, C	
2″	27.8, CH	β: 3.42, m	28.5, CH	β: 3.36, m	28.1, CH	3.36, m	28.4, CH	β: 3.35, m
3″	25.1, CH_2_	α: 1.49, br d (13.6)	26.5, CH_2_	α: 1.48, m	26.3, CH_2_	1.45, m	26.8, CH_2_	α:1.49, m
		β: 2.15, m		β: 2.09, m		2.08, s		β: 1.99, m
4″	19.6, CH_2_	α: 1.82, m	20.2, CH_2_	α: 1.79, m	19.9, CH_2_	1.77, m	19.7, CH_2_	α: 1.70, m
		β: 1.89, br s		β: 2.00, m		1.99, m		β: 1.96, m
5″	40.8, CH	α: 2.48, br s	41.5, CH	α: 2.75, m	41.7, CH	2.24, m	40.3, CH	α: 2.81, m
6″	132.6, C		132.6, C		126.7, C		132.5, C	
7″	132.6, C		117.5, C		132.1, C		117.8, C	
8″	115.9, C		119.9, C		119.5, C		120.3, C	
9″	147.8, C		146.0, C		138.0, C		145.8, C	
9″OH		11.94						
10″	142.7, C		138.3, C		145.6, C		138.2, C	
11″	33.1, CH	1.75, m	34.3, CH	1.66, sptd (6.9)	33.3, CH	1.85, m	34.6, CH	1.70, m
12″	21.6, CH_3_^e^	0.82, d (6.9)	20.4, CH_3_	0.69, d (6.9)	20.7, CH_3_	0.72, d (6.9)	22.0, CH_3_	0.64, d (6.7)
13″	20.0, CH_3_	0.79, d (6.9)	22.2, CH_3_	0.67, d (6.9)	22.2, CH_3_	0.83, d (6.9)	20.0, CH_3_	0.61, d (6.7)
14″	21.6, CH_3_^e^	1.23, d (6.9)	23.0, CH_3_	1.21, d (6.9)	22.6, CH_3_	1.21, d (6.9)	22.7, CH_3_	1.21, d (7.0)
15″	198.8, CH	9.88, s	67.2, CH_2_	4.73, d (12.9)	66.9, CH_2_	4.73, d (13.1)	67.0, CH_2_	4.68, d (13.0)
				5.65, d (12.9)		5.64, d (13.1)		5.64, d (13.0)

a ^1^H 500 MHz.

b ^1^H 700 MHz.

c ^13^C 125 MHz.

d ^13^C 175 MHz.

e Overlapped.

f ^1^H/^13^C chemical shifts not shown due to the absence of corresponding signals.

g ^13^C chemical shifts were extracted from the HMBC NMR spectrum, since compound **3** was degraded after the measurement of the ^1^H, COSY, HSQC, and HMBC NMR data.

Bis-heimiomycin B (**2**), C (**3**), and D (**4**) were isolated as closely related congeners of **1**. With the molecular formula C_36_H_40_O_9_, bis-heimiomycin B (**2**) implies the loss of an oxygen in comparison to **1**. Proton NMR (Table 1) and HSQC data of **2** indicated the presence of an additional oxymethylene (H_2_-15″). HMBC correlations from H_2_-15″ to C-5′/C-6″/C-7″/C-8″/C-9″/C-10″ revealed a ring closure at C-15″ after elimination of an oxygen. In the 1D and 2D NMR spectra of bis-heimiomyin C (**3**) the presence of another oxymethylene group (H_2_-15) and the absence of the aldehyde H-15 occurred as a key difference in **2**. Interactions from H_2_-15 to C-7/C-8/C-9 obtained from HMBC data were consistent with the reduction of **3** at C-15″. Finally, bis-heimiomycin D (**4**) with the molecular formula C_36_H_40_O_8_ implied the loss of another oxygen in comparison to **2**, affording a second ring closure at C-15, leading to a symmetric molecule as observed for **1**. This was confirmed by the replacement of the aldehyde H-15/H-15″ by an oxymethylene H_2_-15/H_2_-15″. Relative and absolute configurations of **2**–**4** were deduced from **1** due to comparison of ECD spectra (Figure S1), ^13^C NMR, and ROESY data to those of **1** and other calamene-type compounds described before,^3^ as well as the common biological source of these compounds.

Heimiomycin D (**5**) was isolated as a red oil from extracts of solid rice cultures. It was shown to possess the molecular formula C_22_H_24_O_7_ by HRESIMS data according to the molecular ion cluster at *m*/*z* 401.1593 [M + H]^+^ requiring 11 degrees of unsaturation. The proton (Table 2) and HSQC NMR spectra of **5** were highly similar to those of heimiomycin C (**14**).^3^ The key difference is an additional hydroxy group at position C-5′. The relative and absolute configurations were deduced as 2*S*,5*R* from **14** by comparison of ECD spectra and ^13^C and ROESY data (Figure S2) to those of **14**(3) and due the common source of both compounds.

**Table 2:** ^13^C and ^1^H NMR Spectroscopic Data of Compounds **5** and **6** in Acetone-*d*_6_ and **7** and **8** in Methanol-*d*_4_ (δ in ppm)

	**5**^b^^,^^d^		**6**^a^^,^^c^		**7**^a^^,^^c^		**8**^a^^,^^c^	
no.	δ_C_, type	δ_H_ (*J* in Hz)	δ_C_, type	δ_H_ (*J* in Hz)	δ_C_, type	δ_H_ (*J* in Hz)	δ_C_, type	δ_H_ (*J* in Hz)
1	139.0, C		139.2, C		144.8, C		130.6, C	
2	28.6, CH	β: 3.39, m	28.6, CH	3.41, dq (6.8, 6.8)	35.1, CH	β: 2.32, m	121.4, C	
3	26.4, CH_2_	α: 1.46, m	26.3, CH_2_	α: 1.48, m	30.7, CH_2_	α: 1.88, m	146.8, C	
		β: 2.07, m		β: 2.08, m		β: 1.22, m		
4	20.0, CH_2_	1.80, m	19.9, CH_2_	1.83, m	21.4, CH_2_	α: 1.69, m	142.0, C	
						β: 1.46, m		
5	40.8, CH	α: 2.51, m	41.0, CH	α: 2.46, br s	43.6, CH	α: 2.14, m	132.6, C	
6	133.4, C		133.3, C		131.3, C		124.0, C	
7	126.5, C		124.7, C		138.2, CH	7.04, d (2.4)	136.7, CH	7.98, s
8	117.4, C		117.2, C		125.5, C		126.6, C	
9	148.3, C		148.6, C		22.4, CH_2_	2.56, m	22.7, CH_2_	2.43, br dd (8.2, 8.2)
9OH		11.84, s		11.86				
10	142.8, C		143.6, C		28.1, CH_2_	2.18, m	26.2, CH_2_	2.70, br dd (8.2, 8.2)
11	34.3, CH	1.70, m	34.4, CH	1.67, sptd (6.9, 6.9)	30.9, CH	1.97, m	28.5, CH	3.53, qq (7.2, 7.2)
12	22.2, CH_3_	0.71, d (6.9)	20.6, CH_3_	0.71, d (6.9)	21.3, CH_3_	0.96, d (6.9)	22.1, CH_3_^e^	1.39, d (7.2)^e^
13	20.7, CH_3_	0.69, d (6.9)	22.1, CH_3_	0.75, d (6.9)	17.7, CH_3_	0.73, d (6.9)	22.1, CH_3_^e^	1.39, d (7.2)^e^
14	22.4, CH_3_	1.19, m	22.3, CH_3_	1.21, d (6.8)	18.9, CH_3_	1.01, d (7.0)	12.1, CH_3_	2.18, s
15	199.6, CH	9.76, s	199.2, CH	9.82, s	168.5, C		171.7, C	
1′	183.6, C		f	f				
2′	146.7, C		f	f				
2′OH		6.83, s	f	f				
3′	119.6, C		114.0, C					
4′	183.5, C		f	f				
5′	146.7, C		f	f				
5′OH		6.83, s	f	f				
6′	112.5, C		104.9, CH	6.09, s				
7′	7.8, CH_3_	1.90, s	f	f				

a ^1^H 500 MHz.

b ^1^H 700 MHz.

c ^13^C 125 MHz.

d ^13^C 175 MHz.

e Overlapped.

f ^1^H/^13^C chemical shifts not shown due to the absence of signals.

For heimiomycin E (**6**) a molecular formula of C_21_H_22_O_7_ was identified by HRESIMS data, indicating the formal loss of a CH_2_ fragment in comparison to **5**. Highly similar NMR spectra showed the absence of methyl group C-7′, leaving an sp^2^-hybridized methine (H-6′) as the difference between both. In contrast to **5**, the signals for C-1′, C-2′, C-4′, and C-5′ were not visible, most likely due to tautomerism.

New calamene-type sesquiterpenoids heimiocalamenes A (**7**) and B (**8**) were isolated from the mycelial extracts of liquid cultures. The molecular formula of **7** was assigned as C_15_H_22_O_2_ according to the molecular ion cluster at *m*/*z* 235.1689 [M + H]^+^ in the HRESIMS spectrum. The NMR spectra of **7** were highly similar to those of **9**. Key differences are the loss of the hydroxy group at C-4, resulting in a methylene (H_2_-4) and the replacement of the oxygenated sp^2^-hybridized carbons at C-9 and C-10 by two methylenes (H_2_-9 and H_2_-10). The relative and absolute configurations were deduced as 2*S*,5*R* by analogy to assignments for **9**,^3^ as these compounds were isolated from the same biological source (*Heimiomyces* sp. MUCL 56078) and showed similar ROESY correlations. Heimiocalamene B (**8**) was identified as the 3-hydroxy derivative of **10** due to close similarities of their NMR data. ^13^C (Table 2) and HMBC data revealed the presence of the oxygenated sp^2^-hybridized carbon at position C-3. We propose to name compounds **9**–**11** as heimiocalamenes C–E, because they have been isolated from the same biological source and show structural similarities to **7** and **8**.

Minor isomers were observed in the HPLC-MS and NMR data for compounds **1** and **2**. After purification via preparative HPLC, results from the analytical HPLC of compound **1** showed the presence of two peaks with the same molecular mass (Figure S5) in a ratio of 9:1. Since this ratio adjusted spontaneously, it is most likely caused by interconversion of **1** between two different forms of the compound. The presence of two peaks with the same molecular mass was also observed in the HPLC-MS data of compound **2** (Figure S6). This is also reflected in the ^1^H and ^13^C spectra of both **1** and **2**, where additional weak signals of the minor isomers can be observed (Tables S4 and S6). However, two possible explanations to cause the presence of these minor isomers can be taken into account. On the one hand, the quinone substructure of **1**–**6** could possess a *para*- or *ortho*-orientation, while both would show similar features, and on the other hand, there is the possibility of atropisomerism within the molecules that would lead to different stereoisomers.

Compounds **1**–**6** were described to possess a *para*-quinone substructure due to comparison of their UV spectra (Figure S7) and ^13^C data (Tables S4–10) to the ones of structurally related *para*- and *ortho*-quinones previously described in the literature.^4^ Especially, absorption maxima at lower wavelengths (maxima with strongest intensity at λ_max_ = 240–300 nm and with medium intensity at λ_max_ = 285–440 nm), characteristic for *para*-quinones, were observed, while the absorption maximum at 500–580 nm, characteristic for *ortho*-quinones, was not observed. However, UV spectra of **1b** and **4** slightly differed from the ones of the other compounds. Therefore, IR spectra of compounds **1b** and **4** (Figures S8 and S9) were measured and compared to the ones of similar quinones described in the literature to support the structural assignment of the *para*-quinone, since for *ortho*-quinones a characteristic and well-separated carbonyl band around 1680–1700 cm^–1^ was not observed.^4^

For compounds **1**–**6** there is the possibility of hindered rotation around the C-3′/C-7 and C-6′/C-7″ bonds. In the case of bis-heimiomycin A (**1**) and heimiomycins D and E (**5** and **6**) a tautomerism within the *p*-benzoquinone substructure is preventing atropisomerism, while compounds **2**–**4** did not show any effects in their ECD data that indicate the presence of atropisomers. The rapid conversion of possible atropisomers can be rationalized by low rotational barriers of the C-7/C-3′ and C-6′/C-7″ bonds. An effective radius of only 1.53 pm and rotational barrier of 27.1 kJ/mol had been determined by Bott et al. for the hydroxy group.^5^ Thus, we assume the keto and hydroxy substituents to be small enough for allowing rotation of the C-7/C-3′ and C-6′/C-7″ bonds.
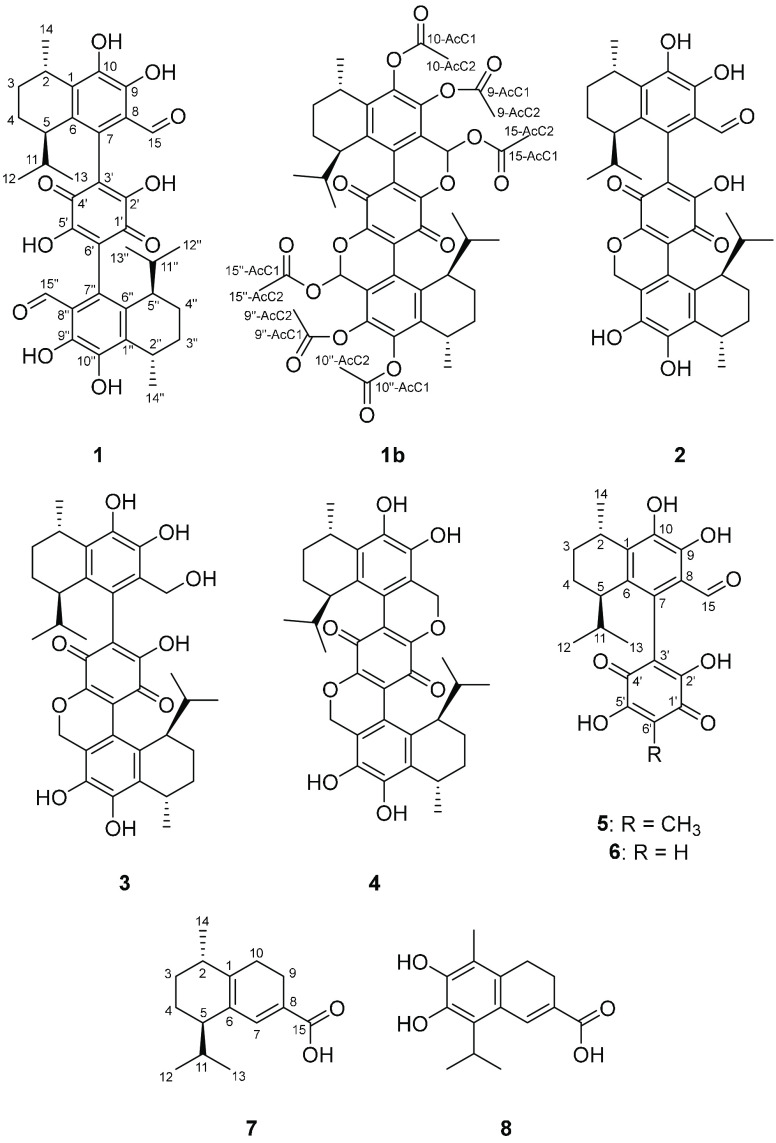


Additionally, the previously described compounds heimiomycin B (**13**),^3^ hispidin (**15**),^6^ and hypholomin B^7^ (**16**) were observed in both liquid and solid cultures (Figures 2 and S4). Hispidin and its derivatives, including hypholomin B, are reported to show antioxidant effects.^8−10^

The variety of secondary metabolites produced by *Heimiomyces* sp. MUCL 56078 can be explained by the combination of calamene-type sesquiterpenoid precursors with various oxidized building blocks. In the case of **1**–**4**, the resulting intermediate undergoes another linkage reaction to a second calamene-type sesquiterpenoid precursor. These reactions might follow a radical mechanism, similar to the biosynthesis of the bibenzoquinone oosporein,^11^ or an electrophilic aromatic substitution mechanism (Figure S5). Both proposed mechanisms are expected to leave the configurations of carbon centers C-2 and C-5 unaffected. Nevertheless, two sesquiterpenoids being linked via a *p*-benzoquinone is an uncommon feature for natural products.^12^ So far only a few similar compounds, like popolohuanones F–H^13,14^ and nakijiquinone E,^15^ have been described in the literature.

However, the structure of the nitrogen-containing derivative **11**, isolated in the preceding study, did not match this logic. In particular, the biogenetic origin of the Schiff base carbon C-15 could not be mechanistically derived from the parental calamene scaffold. Therefore, we carefully reviewed the NMR data of **11** (Table S13) and observed HMBC correlations from H-15 to C-7, C-8, and C-9. By contrast, a correlation from H-15 to C-6 was not observed, which would have been expected for the structure proposed in our earlier study. In addition, ROESY correlations were observed between H-15/9-OMe and H_3_-13/H-5′, respectively, indicating that the linkages of C-15 and C-8 to the calamene moiety have to be exchanged (Figure 2). This assignment does explain the addition of an amino-*p*-benzoquinone to a calamene precursor as a possible biosynthetic pathway to **11**.

All isolated compounds were evaluated for their antimicrobial activities in a serial dilution assay against several Gram positive and Gram negative bacteria as well as fungal strains, but were mostly inactive (Table S1). Furthermore, they were tested for cytotoxicity against the human cervical cancer cell line KB3.1 and the murine fibroblast cell line L929,^16^ where only heimiomycin D (**5**) showed cytotoxic effects against KB3.1 with an IC_50_ of 6.3 μM (Table S2) and therefore was tested against further cell lines (Table S3), resulting in effects on breast cancer cell line MCF-7 (IC_50_ of 2.5 μM), ovarian cancer cell line SKOV-3 (IC_50_ of 3 μM), and skin cancer cell line A431 (IC_50_ of 4.25 μM). Quinone derivatives have been reported to be an important class of molecules, showing a number of various biological activities, presumably due to their ability to undergo nucleophilic attacks and electron reductions.^17,18^ For this reason, compounds **1**–**6** should be considered as candidates for various other targets, such as antiviral or antioxidant assays.

In summary, cultivation of a *Heimiomyces* sp. led to the isolation and identification of new meroterpenoids (**1**–**6**) and two new calamene-type sesquiterpenoids (**7** and **8**), as well as the previously described hispidin (**15**), hypholomin B (**16**), and heimiomycin B (**13**), from shaking and solid rice cultures. Together with the compounds isolated in the preceding study (**9**–**14**),^3^ this *Heimiomyces* sp. showed a vast chemical diversity of its secondary metabolite profile, which emphasizes that Basidiomycota, especially unexplored species from the tropics, should be explored as potentially rich sources of novel natural products in the ongoing search for new drug leads.

## Experimental Section

### General Experimental Procedures

Measurements of the optical rotation were performed using a PerkinElmer 241 polarimeter. UV spectra were obtained using a Shimadzu UV–vis spectrophotometer UV-2450, and ECD spectra were measured using a Jasco J-815 spectropolarimeter. Measurements of the IR spectra were performed using a PerkinElmer FT-IR spectrometer Spectrum 100. NMR spectra were recorded using a Bruker Avance III 500 MHz spectrometer equipped with a BBFO (Plus) SmartProbe (^1^H 500 MHz, ^13^C 125 MHz) and a Bruker Avance III 700 MHz spectrometer equipped with a 5 mm TCI cryoprobe (^1^H 700 MHz, ^13^C 175 MH), and NMR data were referenced to selected chemical shifts of acetone-*d*_6_ (^1^H: 2.05 ppm, ^13^C: 29.32 ppm) and MeOH-*d*_4_ (^1^H: 3.31 ppm, ^13^C: 49.15 ppm), respectively. HRESIMS mass spectra were measured using the Agilent 1200 series HPLC-UV system in combination with an ESI-TOF-MS (Maxis, Bruker). Measurements were performed with a 2.1 × 50 mm, 1.7 μm, C18 Acquity UPLC BEH (Waters) column, using Milli-Q H_2_O + 0.1% formic acid as solvent A and MeCN + 0.1% formic acid as solvent B (gradient: 5% B for 0.5 min increasing to 100% B in 19.5 min and maintaining 100% B for 5 min, flow rate: 0.6 mL/min, UV detection: 200–600 nm).

### Fungal Material

*Heimiomyces* sp. (MUCL 56078) was collected from Mount Elgon National Reserve (1°7′6″ N, 34°31′30″ E) in Kenya by C. Decock and J. C. Matasyoh. Identification of the genus and deposition of a dried specimen were carried out as described by Cheng et al.^3^

### Fermentation and Extraction

Cultures of *Heimiomyces* sp. were maintained on YM6.3 agar plates.

For the seed cultures, three 50 mm^2^ sized pieces of well-grown mycelium from YM6.3 agar plates were transferred into a 500 mL Erlenmeyer shape culture flask containing 200 mL of YM6.3 medium (10 g/L malt extract, 4 g/L d-glucose, 4 g/L yeast extract, pH 6.3). The incubation was performed at 23 °C and 140 min^–1^ on a rotary shaker. After 23 days of cultivation the culture broth was homogenized with an Ultra-Turrax (T25 easy clean digital, IKA), equipped with a S 25 N – 25 F dispersing tool, at 8000 rpm for 10–20 s.

#### Solid Rice Cultures

The inoculum (8 mL per flask) was transferred into four 500 mL Erlenmeyer shape culture flasks containing BRFT medium (1 g/L yeast extract, 0.5 g/L sodium tartrate, 0.5 g/L K_2_HPO_4_, 100 mL of the solution added to 28 g of brown rice). Afterward, the medium was loosened with a spatula to make it accessible for oxygen and homogeneously distribute the inoculum. The incubation was performed at 23 °C in an incubator. After 72 days the fermentation process was stopped. At first, the medium and the mycelium were covered with acetone. Following this, the medium was loosened with a spatula and mixed with the acetone. Extraction was carried out by using ultrasonication for 30 min. Liquid and solid phase were separated by filtration. This procedure was repeated, followed by evaporation (40 °C) of the organic solvent with a rotary evaporator. The remaining aqueous phase was extracted with EtOAc (1:1) in a separatory funnel, twice. The organic phase was evaporated to dryness (40 °C). Furthermore, the extract was dissolved in 5 mL of MeOH. Afterward, 50 mL of a 1:1 mixture of heptane and MeOH/H_2_O (1:1) was added. Extraction was carried out in a separatory funnel twice, and the heptane and aqueous phases were collected separately. Finally, both were evaporated to dryness (40 °C). This led to the isolation of 476 mg of aqueous extract and 206 mg of heptane extract. A second fermentation of six solid rice cultures was performed using the same conditions, leading to the isolation of 1071 mg of aqueous extract (no heptane extraction).

#### Liquid Cultures

The inoculum (3 mL per flask) was transferred into 21 500 mL Erlenmeyer shape culture flasks containing 200 mL of YM6.3 medium and five 500 mL Erlenmeyer shape culture flasks containing 200 mL of MOF medium (75 g/L mannitol, 16.2 g/L MES, 15 g/L oat flour, 5 g/L yeast extract, 4 g/L l-glutamic acid, pH 6.0). The incubation was performed at 23 °C and 140 min^–1^ on a rotary shaker. Glucose consumption was monitored using test strips (Medi-Test Glucose, Macherey-Nagel). The fermentation process was stopped 2 days after the culture broth tested negative for glucose (33 days in total for YM6.3 cultures and 27 days in total for MOF cultures). Mycelium and supernatant were separated by centrifugation at 5100 min^–1^ for 15 min (lab centrifuge 4-16KS, Sigma Laborzentrifugen GmbH). The mycelium was overlaid with acetone and afterward extracted in an ultrasonic bath for 30 min, twice. Solid and liquid phases were separated by filtration, followed by evaporation (40 °C) of the organic solvent with a rotary evaporator. The remaining aqueous phase was diluted with H_2_O and extracted against EtOAc. Following this, the organic phase was evaporated to dryness (40 °C). The supernatant was extracted with EtOAc (1:1) twice in a separatory funnel. The organic phase was kept and evaporated to dryness (40 °C). This led to the isolation of 567 mg of extract from the mycelium and 651 mg of extract from the supernatant of YM6.3 cultures, as well as 211 mg of extract from the mycelium and 339 mg of crude extract from the supernatant of MOF cultures. Filtration of the extracts was performed by using an SPME Strata-X 33 μm Polymeric RP cartridge (Phenomenex, Inc.).

### Analytical HPLC

The obtained extracts were dissolved in acetone to yield a concentration of 10 mg/mL. Solvation was aided by ultrasonication at 40 °C for 10 min. Samples were analyzed by an analytical HPLC device (Dionex UltiMate 3000 series) coupled to an ion trap mass spectrometer (amazon speed by Bruker) to conduct the measurements. HPLC grade H_2_O and HPLC grade MeCN supplemented by 0.1% formic acid were used as mobile phase. With a flow rate of 600 μL/min, 2 μL of the injected samples was separated over an ACQUITY-UPLC BEH C18 column (50 × 2.1 mm; particle size: 1.7 μm) by Waters. Starting with 5% of MeCN, the amount was increased to 100% in 20 min and retained for 5 min at 100%. The obtained chromatograms were evaluated with the appropriate Bruker analysis software (Data Analysis 4.4).

### Isolation of Compounds **1**–**8**

After evaluation of the analytical data, the extracts were separated via RP HPLC using a Gilson PLC 2250 purification system.

#### Solid Rice Cultures (BRFT)

The extract obtained from the aqueous phase of the solid rice cultures of the first fermentation was purified using a Gemini LC column 250 × 50 mm, 110 Å, 10 μm (Phenomenex); solvent A: Milli-Q H_2_O + 0.1% formic acid, solvent B: MeCN + 0.1% formic acid, flow rate: 60 mL/min, gradient: 5 min B at 25%, increasing to 80% B in 55 min, increasing to 100% B in 10 min, maintaining 100% B for 10 min. The fraction at 60.5–61.5 min led to 2.76 mg of **3**, the fraction at 63.5–64.5 min led to 6.8 mg of compound **4**, and the fraction at 65.5–66.5 min led to 2.8 mg of compound **2**. The extract obtained from the solid rice cultures of the second fermentation was purified using a Synergi Polar RP 250 × 50 mm, 80 Å, 10 μm (Phenomenex) column; solvent A: Milli-Q H_2_O + 0.1% formic acid, solvent B: MeCN + 0.1% formic acid, flow rate: 60 mL/min, gradient: 5 min B at 20%, increasing to 40% B in 5 min, increasing to 85% B in 50 min, increasing to 100% B in 5 min, maintaining 100% B for 10 min. The fraction at 29.5–30.5 min led to 7.4 mg of **6**, the fraction at 37.0–37.75 min led to 4.5 mg of compound **5**, and the fraction at 48.0–48.75 min led to 43.2 mg of compound **1**.

#### Liquid Cultures (YM6.3)

The extract obtained from the supernatant was purified using a Gemini LC column 250 × 50 mm, 110 Å, 10 μm (Phenomenex); solvent A: Milli-Q H_2_O + 0.1% formic acid, solvent B: MeCN + 0.1% formic acid, flow rate: 60 mL/min, gradient: 5 min B at 20%, increasing to 65% B in 50 min, increasing to 100% B in 20 min, maintaining 100% B for 10 min. The fraction at 36.5–37.5 min led to 4.2 mg of **8**.

#### Liquid Cultures (MOF)

The extract obtained from the mycelium of the culture broth was purified using a Gemini LC column 250 × 50 mm, 110 Å, 10 μm (Phenomenex); solvent A: Milli-Q H_2_O + 0.1% formic acid, solvent B: MeCN + 0.1% formic acid, flow rate: 50 mL/min, gradient: 5 min B at 30%, increasing to 50% B in 10 min, increasing to 100% B in 50 min, maintaining 100% B for 10 min. The fraction at 48.0–49.0 min led to 2.5 mg of **7**.

### Acetylation of **1**

Acetylation was performed as previously described by Duncan et al.;^19^ 16 mg of **1** was dissolved in 4.8 mL of pyridine, and afterward 2.4 mL of acetic anhydride (resulting in a 2:1 mixture) was added. The solution was left at room temperature for 3–4 h. Following this, the reagents were removed by evaporation (40 °C) with a rotary evaporator. The product was dissolved in acetone and analyzed by HPLC/MS. Due to side product formation, a purification was performed via RP HPLC using a Gilson PLC 2050 purification system. The sample was purified using an XBridge Prep C18 column, 19 × 250 mm, 5 μm (Waters); solvent A: Milli-Q H_2_O + 0.1% formic acid, solvent B: MeCN + 0.1% formic acid, flow rate: 20 mL/min, gradient: 5 min B at 70%, increasing to 90% B in 35 min, increasing to 100% B in 5 min, maintaining 100% B for 5 min. The fraction at 20.5–21.5 min led to 3.3 mg of compound **1b**.

#### Bis-heimiomycin A (**1**):

red oil; [α]^25^_D_ +16 (*c* 0.01, acetone); UV/vis (0.01 mg/mL, MeCN) λ_max_ (log ε) 372 (3.89), 283 (4.55), 245 (4.45) nm; ECD (0.2 mg/mL, MeOH) λ (Δε) 297 (+32.5), 261 (−34.0), 240 (+39.9), 204 (−29.5) nm, Figure S1; ^1^H and ^13^C NMR data (acetone-*d*_6_), Table 1; ESIMS *m*/*z* 633.30 [M + H]^+^, 631.11 [M – H]^−^; HRESIMS *m*/*z* 633.2695 [M + H]^+^ (calcd for C_36_H_41_O_10_, 633.2694); *t*_R_ = 13.3 min (analytical HPLC).

#### Bis-heimiomycin B (**2**):

red oil; [α]^25^_D_ +150 (*c* 0.01, MeOH); UV/vis (0.01 mg/mL, MeOH) λ_max_ (log ε) 378 (3.78), 287 (4.14), 208 (4.40) nm; ECD (0.2 mg/mL, MeOH) λ (Δε) 342 (+6.0), 315 (+1.3), 295 (+20.8), 245 (−2.3), 223 (+17.0), 200 (−11.6), 194 (+2.5) nm, Figure S1; ^1^H and ^13^C NMR data (acetone-*d*_6_), Table 1; ESIMS *m*/*z* 617.31 [M + H]^+^, 615.37 [M – H]^−^; HRESIMS *m*/*z* 617.2749 [M + H]^+^ (calcd for C_36_H_41_O_9_, 617.2745); *t*_R_ = 13.3 min (analytical HPLC).

#### Bis-heimiomycin C (**3**):

red oil; [α]^25^_D_ +130 (*c* 0.01, MeOH); UV/vis (0.01 mg/mL, MeOH) λ_max_ (log ε) 373 (3.93), 293 (4.16), 205 (4.54) nm; ECD (0.2 mg/mL, MeOH) λ (Δε) 315 (+2.8), 293 (+9.9), 264 (+3.7), 242 (+8.2), 230 (+2.1) nm, Figure S1; ^1^H and ^13^C NMR data (acetone-*d*_6_), Table 1; ESIMS *m*/*z* 601.32 [M – H_2_O + H]^+^, 617.36 [M – H]^−^; HRESIMS *m*/*z* 641.2720 [M + Na]^+^ (calcd for C_36_H_42_NaO_9_, 641.2721); *t*_R_ = 12.5 min (analytical HPLC).

#### Bis-heimiomycin D (**4**):

red oil; [α]^25^_D_ −290 (*c* 0.01, MeOH); UV/vis (0.01 mg/mL, MeOH) λ_max_ (log ε) 372 (3.86), 327 (4.17), 303 (4.15), 204 (4.59) nm; ECD (0.2 mg/mL, MeOH) λ (Δε) 387 (+6.7), 328 (−5.5), 293 (+17.8), 267 (+10.0), 261 (+10.4), 251 (+6.8), 240 (+15.0), 233 (+11.9), 218 (+29.8), 200 (−17.1) nm, Figure S1; ^1^H and ^13^C NMR data (acetone-*d*_6_), Table 1; ESIMS *m*/*z* 601.31 [M + H]^+^, 599.35 [M – H]^−^; HRESIMS *m*/*z* 601.2797 [M + H]^+^ (calcd for C_36_H_41_O_8_, 601.2796); *t*_R_ = 12.9 min (analytical HPLC).

#### Heimiomycin D (**5**):

red oil; [α]^25^_D_ +27 (*c* 0.01, acetone); UV/vis (0.01 mg/mL, MeCN) λ_max_ (log ε) 305 (3.86) nm; ECD (0.2 mg/mL, MeCN) λ (Δε) 299 (+16.7), 259 (−1.1), 223 (+13.8) nm, Figure S2; ^1^H and ^13^C NMR data (acetone-*d*_6_), Table 2; ESIMS *m*/*z* 401.16 [M + H]^+^, 398.94 [M – H]^−^; HRESIMS *m*/*z* 401.1593 [M + H]^+^ (calcd for C_22_H_25_O_7_, 401.1595); *t*_R_ = 9.8 min (analytical HPLC).

#### Heimiomycin E (**6**):

red oil; [α]^25^_D_ +170 (*c* 0.01, MeCN); UV/vis (0.01 mg/mL, MeCN) λ_max_ (log ε) 369 (3.73), 283 (4.33), 240 (4.18) nm; ECD (0.2 mg/mL, MeCN) λ (Δε) 295 (+36.9), 275 (−9.1), 255 (−1.3), 246 (−4.0), 204 (+5,1) nm, Figure S2; ^1^H and ^13^C NMR data (acetone-*d*_6_), Table 2; ESIMS *m*/*z* 387.14 [M + H]^+^, 384.93 [M – H]^−^; HRESIMS *m*/*z* 387.1437 [M + H]^+^ (calcd for C_21_H_23_O_7_, 387.1438); *t*_R_ = 8.4 min (analytical HPLC).

#### Heimiocalamene A (**7**):

yellow oil; [α]^25^_D_ +53 (*c* 0.01, acetone); UV/vis (0.01 mg/mL, MeCN) λ_max_ (log ε) 305 (3.86) nm; ECD (0.2 mg/mL, MeOH) λ (Δε) 307 (−2.1), 254 (+1.0), 235 (+0.4), 202 (+2.5), 194 (−0.03) nm, Figure S3; ^1^H and ^13^C NMR data (acetone-*d*_6_), Table 2; ESIMS *m*/*z* 235.10 [M + H]^+^, 232.90 [M – H]^−^; HRESIMS *m*/*z* 235.1689 [M + H]^+^ (calcd for C_15_H_23_O_2_, 235.1693); *t*_R_ = 11.7 min (analytical HPLC).

#### Heimiocalamene B (**8**):

yellow oil; [α]^25^_D_ +70 (0.01, MeOH); UV/vis (0.01 mg/mL, MeOH) λ_max_ (log ε) 310 (3.59), 246 (3.75), 198 (4.47) nm; ^1^H and ^13^C NMR data (MeOH-*d*_4_), Table S2; ESIMS *m*/*z* 263.05 [M + H]^+^, 260.82 [M – H]^−^; HRESIMS *m*/*z* 263.1275 [M + H]^+^ (calcd for C_15_H_19_O_4_, 263.1275); *t*_R_ = 6.1 min (analytical HPLC).

## Supporting Information Available

ECD spectra of compounds **1**–**7**; protocol: antimicrobial and cytotoxicity assay, Tables S1–S3; structures of compounds **15** and **16**, Figure S4; ^13^C and ^1^H NMR data of compound **1b**, Table S5; 1D and 2D NMR data of compounds **1**–**8** and **11**, Tables S4–S13; 1D and 2D NMR spectra of compounds **1**–**8** and **11** (PDF)
