# Supplementary material for: Calamene-Type Sesqui-, Mero-, and Bis-sesquiterpenoids from Cultures of Heimiomyces sp., a Basidiomycete Collected in Africa
Source: J Nat Prod. 2023 Feb 13;86(2):390–7. doi: 10.1021/acs.jnatprod.2c01015 (PMC9972471; doi:10.1021/acs.jnatprod.2c01015)

Supporting information

SI Experimental Section

ECD spectra of compounds 1-7.

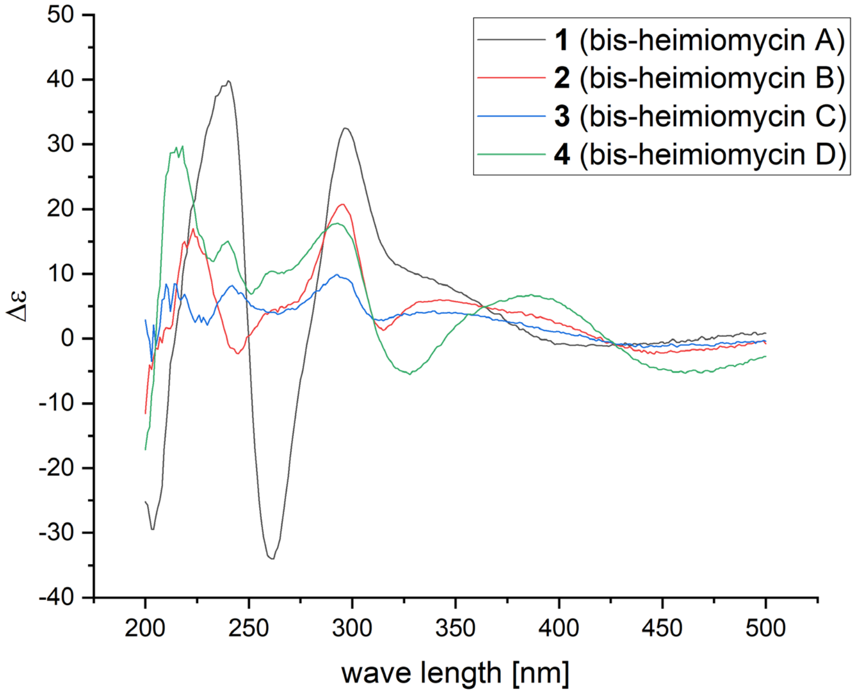

Figure S1. ECD spectra of **1-4**.

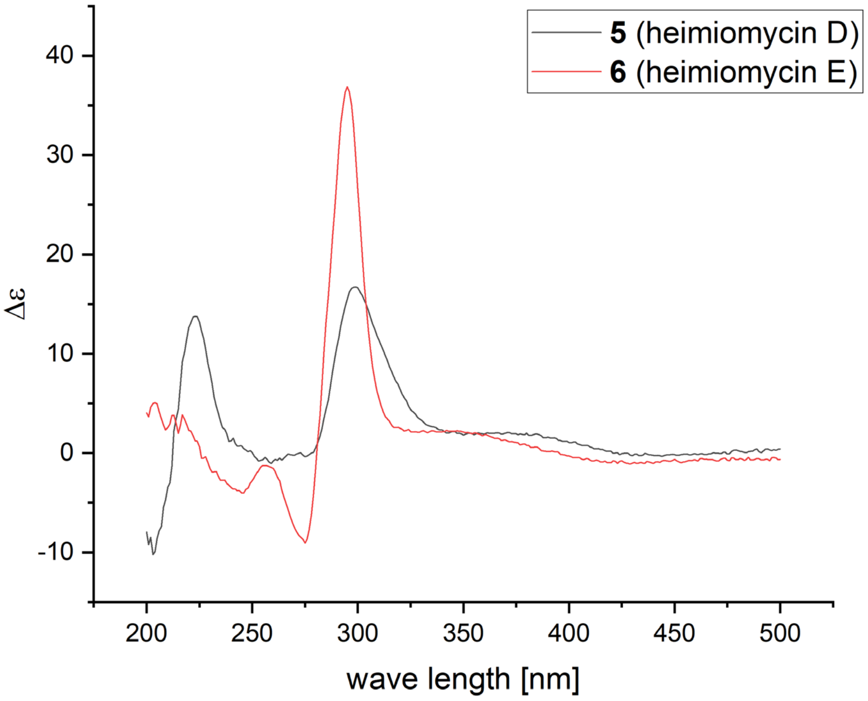

Figure S2. ECD spectra of **5** and **6.**

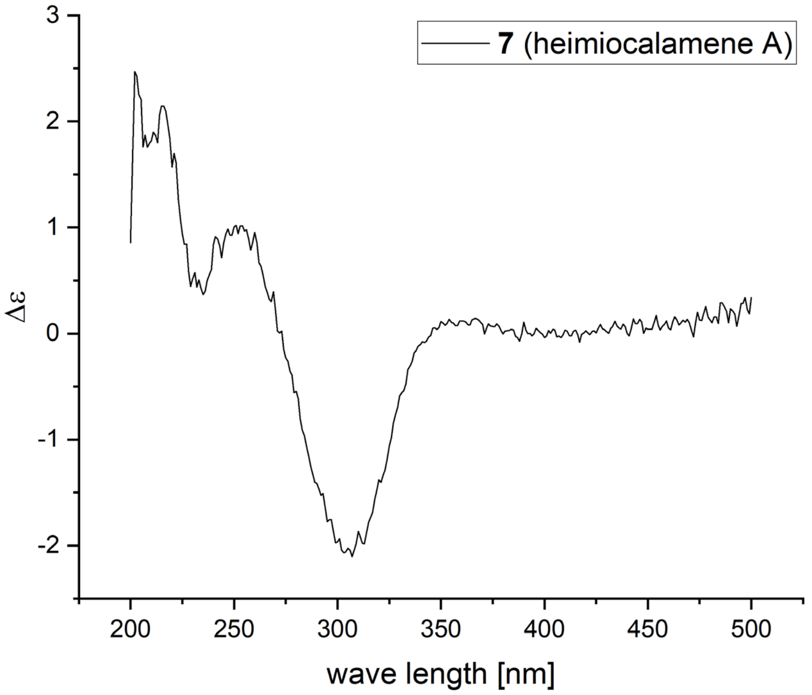

Figure S3. ECD spectra of **7**.

Evaluation of antimicrobial activity.
To assess the antimicrobial activity of each compound, a serial dilution assay to ascertain their minimum inhibitory concentration (MIC) against several yeast, fungal and bacterial strains was carried out in 96-well microtiter plates, as previously described by Harms et al [2].

Table S1. Minimum inhibitory concentration (MIC in µg/mL) for yeast, bacterial and fungal strains.

The serial dilution assay was performed using 1 mg/mL stock solutions of **1**-**8**, **13**, **15** and **16**. As positive control the antimicrobials [O] Oxytetracyclin, [G] Gentamycin and [K] Kanamycin were used for bacteria and [N] Nystatin was used for filamentous fungi and yeast. Methanol and acetone (20 µL) were tested as negative control and did not show inhibitory effects. – no inhibition was observed. Compounds **1**-**8**, **13**, **15** and **16** did not show activities against *Schizosaccharomyces pombe* (DSM 70572), *Pichia anomala* (DSM 6766), *Candida albicans* (DSM 1665), *Escherichia coli* (DSM 1116), *Mycobacterium smegmatis* (ATCC 700084), *Pseudomonas aeruginosa* (DSM PA14), *Chromobacterium violaceum* (DSM 30191) and *Acinetobacter baumannii* (DSM 30008).

**compound**	***Bacillus subtilis DSM 10***	***Staphylococcus aureus DSM 346***	***Mucor hiemalis DSM 2656***	***Rhodotorula glutinis DSM 10134***
**1** (Bis-Heimiomycin A)	33.3	-	-	66.6
**2** (Bis-Heimiomycin B)	16.6	33.3	-	-
**3** (Bis-Heimiomycin C)	16.6	33.3	-	-
**4** (Bis-Heimiomycin D)	-	-	-	-
**5** (Heimiomycin D)	33.3	33.3	-	-
**6** (Heimiomycin E)	16.6	66.6	-	-
**7** (Heimiocalamene A)	66.6	66.6	33.3	66.6
**8** (Heimiocalamene B)	66.6	-	-	66.6
**13** (Heimiomycin B)	66.6	66.6	-	-
**15** (Hispidin)	-	-	-	-
**16** (Hypholomin B)	33.3	-	-	-
positive control	*8.3 [O]*	*0.83 [O]*	*8.3 [N]*	*4.2 [N]*

IC_{50} in µg/ml; - no activity

Evaluation of cytotoxicity.

Assessment of in vitro cytotoxicity was performed in 96-well plates, as previously published by Harms et al [1] against the mouse fibroblast cell line L929 and the cervix carcinoma cell line KB3.1. Heimiomycin D (**5**) was additionally tested against human breast carcinoma cell line MCF-7, human lung carcinoma cell line A549, human ovarian carcinoma cell line SKOV-3, human prostate carcinoma cell line PC-3 and human epidermoid carcinoma cell line A431.

Table S2. Half inhibitory concentration (IC_{50} in µM).

The cytotoxicity assay was performed using 1 mg/mL stock solutions of **1**-**8**, **13**, **15** and **16**. Epothilon B was tested as positive control. Methanol and acetone (20 µL) were tested as negative control and did not show inhibitory effects. – no inhibition was observed.

compound	KB3.1	L929
**1** (Bis-Heimiomycin A)	-	-
**2** (Bis-Heimiomycin B)	30.8	-
**3** (Bis-Heimiomycin C)	27.5	32.4
**4** (Bis-Heimiomycin D)	35.0	-
**5** (Heimiomycin D)	6.3	45.0
**6** (Heimiomycin E)	-	57.0
**7** (Heimiocalamene A)	85.5	-
**8** (Heimiocalamene B)	31.3	37.4
**13** (Heimiomycin B)	34.6	-
**15** (Hispidin)	93.5	85.4
**16** (Hypholomin B)	-	-
Epo B (positive control)	*$5{,}5 \times 10^{-5}$*	*$5{,}8 \times 10^{-3}$*

IC_{50} in µM; - no activity

Table S3. Half inhibitory concentration (IC_{50} in µM) of **5**.

The extended cytotoxicity assay against further cell lines was performed using a 1 mg/mL stock solution of **5**. Epothilon B was tested as positive control. Acetone (20 µL) was tested as negative control and did not show inhibitory effects.

	Heimiomycin D (5)	Epo B
**MCF-7**	2.5	3.0×10^{-5}
**A549**	14.3	6.7×10^{-5}
**SKOV-3**	3.0	1.6×10^{-4}
**PC-3**	30.0	1.2×10^{-4}
**A431**	4.3	5.1×10^{-5}

IC_{50} in µM

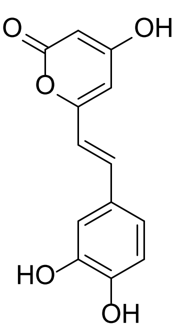

15

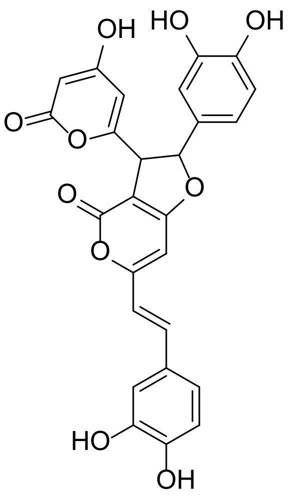

16

Figure S4. Known compounds isolated from *Heimiomyces* sp. **15**: hispidin, **16**: hypholomi

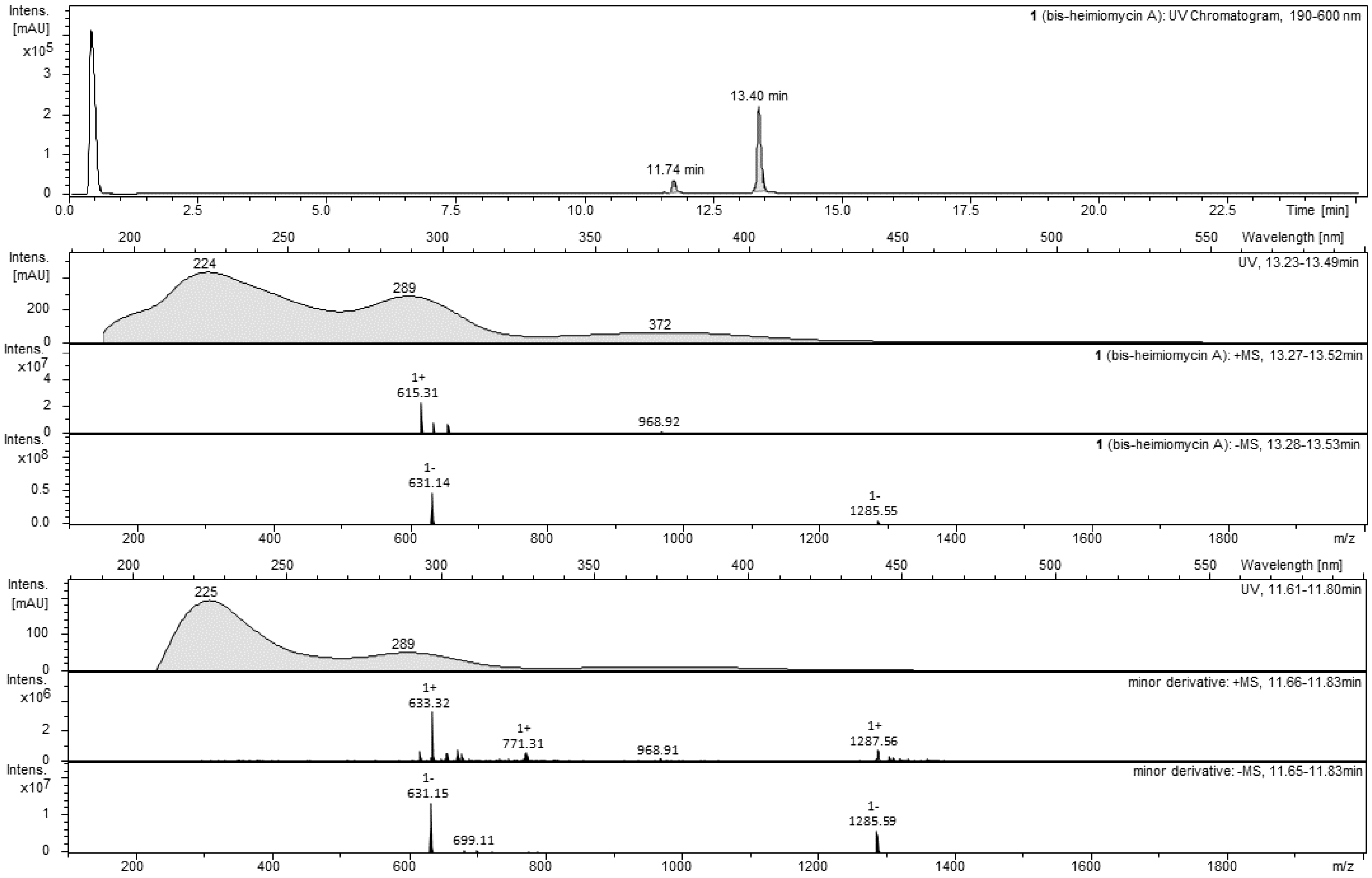

Figure S5. HPLC-UV/vis chromatogram at 190-600 nm, DAD, and ESI-MS (+/-) traces of compound **1**

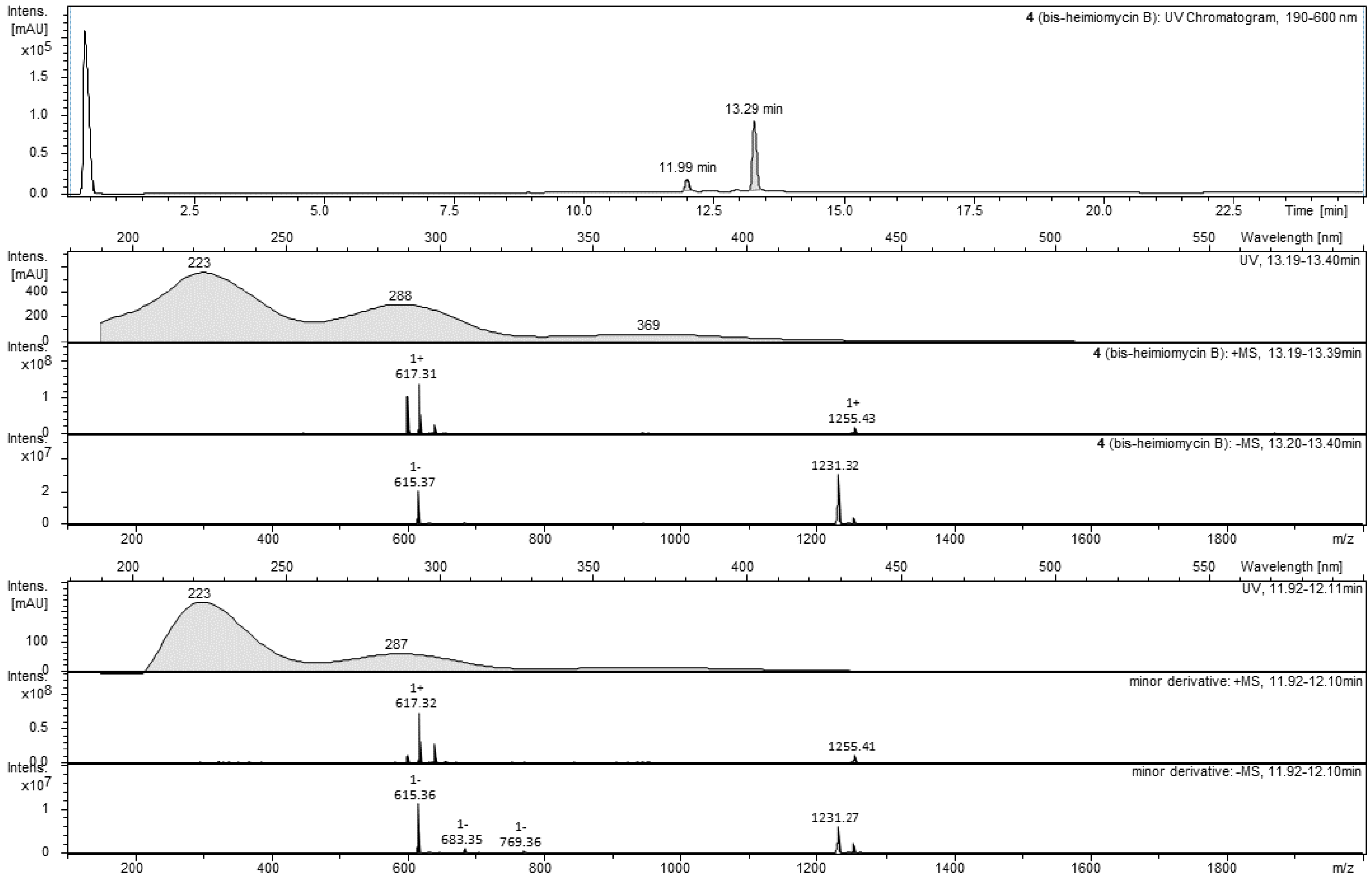

Figure S6. HPLC-UV/vis chromatogram at 190-600 nm, DAD, and ESI-MS (+/-) traces of compound **2**

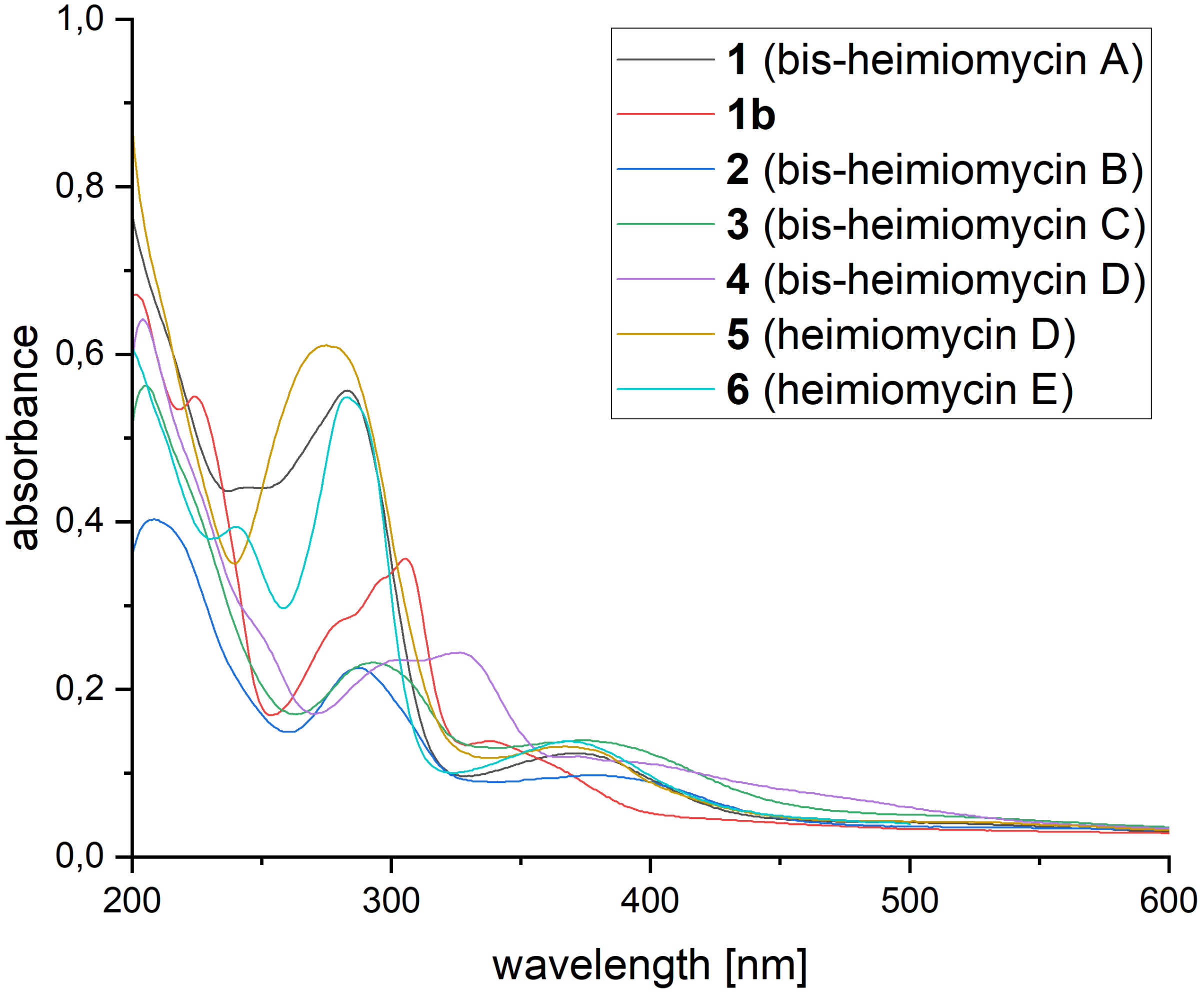

Figure S7. UV/vis chromatogram at 200-600 nm of compounds **1**-**6**.

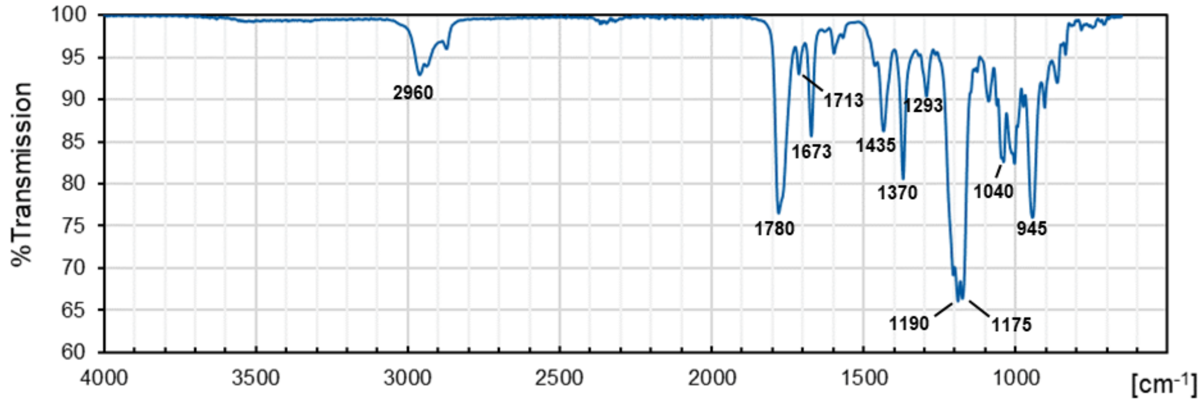

Figure S8. IR spectrum (ATR) of compound **1b** from 4000 to 650 cm^{-1}

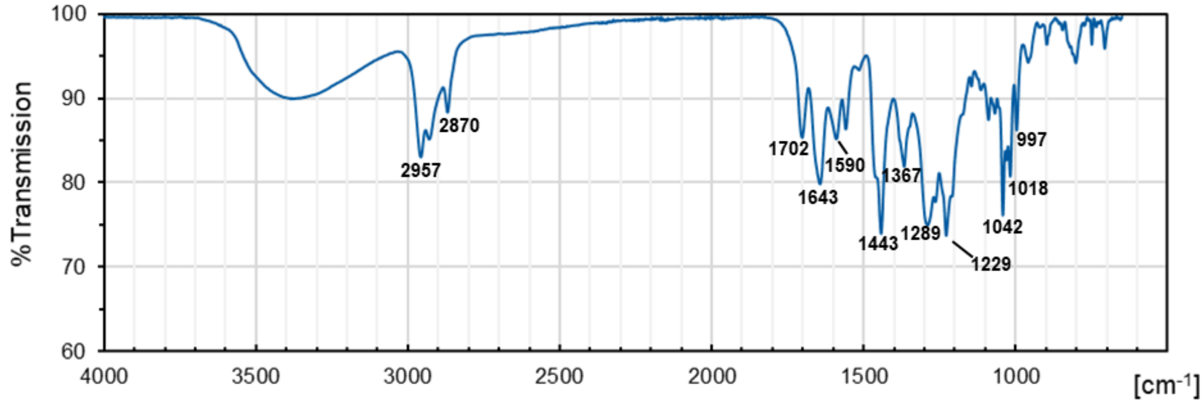

Figure S9. IR spectrum (ATR) of compound **4** from 4000 to 650 cm^{-1}

References

(1) Harms, K.; Surup, F.; Stadler, M.; Stchigel, A. M.; Marin-Felix, Y. *Microorganisms*. 2021, p 1191.

NMR spectroscopic data of 1-8 and 11.

Table S4. NMR Data (^{1}H, 500 MHz, [13]C 175 MHz) of compound **1** in Acetone-d_6 (δ in ppm)

No.	δ_C , type	δ_H (J in Hz)	COSY	C to H HMBC	N/ROESY	δ_C , type[a]	δ_H (J in Hz)[a]
1	137.9, C						
2	27.8, CH	β: 3.42, m	14, 3β	4, 14, 3, 6, 1, 10		27.68, CH	
3	25.1, CH_2	α: 1.49, br d (13.6)<br>β: 2.15, m	4α, 4β, 3β<br>3α, 2β	4, 14, 2, 5, 1	14<br>13	24.86, CH_2	
4	19.6, CH_2	α: 1.82, m<br>β: 1.89, br s	3α, 5α<br>3α	3, 5	14, 5α<br>12	19.60, CH_2	
5	40.8, CH	α: 2.48, br s	11, 4α	3, 6	12, 4α	39.74, CH	2.62, br s
6	132.6, C						
7	132.6, C						
8	115.9, C						
9	147.8, C						
9OH		11.94		8, 10, 9			11.76
10	142.7, C						
11	33.1, CH	1.75, m	12, 5α	4, 13, 12, 5, 6		33.60, CH	
12	21.6, CH_3[†]	0.82, d (6.9)	11	13, 11, 5	4β, 5α		
13	20.0, CH_3	0.79, d (6.9)		12, 11, 5	3β		
14	21.6, CH_3[†]	1.23, d (6.9)	2β	3, 2, 1	3α, 4α		
15	198.8, CH	9.88, s		8, 10, 9		197.20, CH	9.78, s
1'	-	-					
2'	147.5, C						
3'	-	-					
4'	-	-					
5'	147.5, C						
6'	-	-					
1"	137.9, C						
2"	27.8, CH	β: 3.42, m	14", 3"β	4", 14", 12", 3", 6", 1", 10"		27.68, CH	
3"	25.1, CH_2	α: 1.49, br d (13.6)<br>β: 2.15, m	4"α, 4"β, 3"β<br>3"α, 2"β	4", 14", 2", 5", 1"	14"<br>13"	24.86, CH_2	
4"	19.6, CH_2	α: 1.82, m<br>β: 1.89, br s	3"α, 5"α<br>3"α	3", 5"	14", 5"α<br>12"	19.60, CH_2	
5"	40.8, CH	α: 2.48, br s	11", 4"α	3", 6"	12", 4"α	39.74, CH	2.62, br s
6"	132.6, C						
7"	132.6, C						
8"	115.9, C						
9"	147.8, C						
9"OH		11.94		8", 10", 9"			11.76
10"	142.7, C						
11"	33.1, CH	1.75, m	12", 5"α	4", 13", 5", 6"		33.60, CH	
12"	21.6, CH_3[†]	0.82, d (6.9)	11, 11"	13", 11", 5"	4"β, 5"α		
13"	20.0, CH_3	0.79, d (6.9)		11", 5"	3"β		
14"	21.6, CH_3[†]	1.23, d (6.9)	2"β	3", 2", 1"	3"α, 4"α		

15"	198.8, CH	9.88, s	8", 10", 9"	197.20, CH	9.78, s

† overlapped; - $^{1}H/^{13}C$ chemical shifts not shown due to absence of corresponding signals; [a] deviating $^{1}H/^{13}C$ shifts of minor isomers

Table S5. NMR Data (^{1}H, 700 MHz, ^{13}C 175 MHz) of compound **1b** in Acetone-d_6 (δ in ppm)

No.	δ_C , type	δ_H (J in Hz)	COSY	C to H HMBC
1	139.0, C			
2	28.7, CH	3.13, m	14, 3	4, 14, 3, 1, 6, 10
3	25.3, CH_2	1.56, m<br>2.03, m	4, 4<br>2	2, 11, 5, 6
4	18.5, CH_2	1.79, m<br>2.04, m	3, 4, 5<br>3, 4, 5	2, 5, 1<br>12, 11, 5
5	40.9, CH	2.88, m	11, 4, 4	13, 3, 11, 8, 7, 3', 1, 6
6	140.2, C			
7	122.0, C			
8	121.7, C			
9	137.3, C			
9-AcC1	167.7, C†			
9-AcC2	19.2, CH_3	2.29, s		9, 9-AcC1
10	142.6, C			
10-AcC1	167.7, C†			
10-AcC2	19.5, CH_3	2.38, s		10, 10-AcC1
11	34.0, CH	1.65, m	13, 5	13, 12, 5, 1
12	20.8, CH_3	0.63, d (6.7)		13, 11, 5
13	18.8, CH_3	0.65, d (6.7)	11	12
14	22.4, CH_3	1.21, d (6.9)	2	3, 2, 1, 6
15	85.8, CH	7.65, s		8, 9, 2', 15-AcC1
15-AcC1	168.2, C			
15-AcC2	19.6, CH_3	1.96, s		15, 15-AcC1
1'	176.4, C			
2'	149.1, C			
3'	122.3, C			
4'	176.4, C			
5'	149.1, C			
6'	122.3, C			
1"	139.0, C			
2"	28.7, CH	3.13, m	14", 3"	4", 14", 3", 1", 6", 10"
3"	25.3, CH_2	1.56, m<br>2.03, m	4", 4"<br>2"	2", 11", 5", 6"
4"	18.5, CH_2	1.79, m<br>2.04, m	3", 4", 5"<br>3", 4", 5"	2", 5", 1"<br>12", 11", 5"
5"	40.9, CH	2.88, m	11", 4", 4"	13", 3", 11", 8", 7", 6', 1", 6"
6"	140.2, C			
7"	122.0, C			
8"	121.7, C			
9"	137.3, C			
9"-AcC1	167.7, C†			
9"-AcC2	19.2, CH_3	2.29, s		9", 9"-AcC1
10"	142.6, C			
10"-AcC1	167.7, C†			
10"-AcC2	19.5, CH_3	2.38, s		10", 10"-AcC1
11"	34.0, CH	1.65, m	13", 5"	13", 12", 5", 1"

12"	20.8, CH_3	0.63, d (6.7)		13", 11", 5"
13"	18.8, CH_3	0.65, d (6.7)	11"	12"
14"	22.4, CH_3	1.21, d (6.9)	2"	3", 2", 1", 6"
15"	85.8, CH	7.65, s		8", 9", 5', 15"-AcC1
15"-AcC1	168.2, C			
15"-AcC2	19.6, CH_3	1.96, s		15", 15"-AcC1

† overlapped

Table S6. NMR Data (^{1}H, 700 MHz, ^{13}C 175 MHz) of compound **2** in Acetone-d_6 (δ in ppm)

No.	δ_C , type	δ_H (J in Hz)	COSY	C to H HMBC	N/ROESY	δ_C , type[a]	δ_H (J in Hz)[a]
1	139.0, C					138.94, C	
2	28.8, CH	β: 3.41, m	14, 3β	4, 14, 3, 6, 1, 10	4β	28.64, CH	
3	26.1, CH_2	α: 1.48, m<br>β: 2.09, m	4β, 3β<br>3α, 2β		14, 5α	25.84, CH_2	
4	20.4, CH_2	α: 1.79, m<br>β: 2.00, m	 3α		 2β	20.38, CH_2	
5	42.0, CH	α: 2.35, br s	11	3, 11, 6, 1	3α	41.37, CH	α: 2.12, br s
6	133.6, C					133.64, C	
7	124.2, C					124.57, C	
8	116.4, C						
9	148.8, C						
9OH		11.90		8, 10, 9			11.66
10	143.5, C					143.54, C	
11	33.6, CH	1.85, m	13, 12, 5α	13, 12, 5, 6			
12	22.2, CH_3	0.84, d (6.9)	11	13, 11, 5			
13	21.1, CH_3	0.78, d (6.9)	11	4, 12, 11, 5			
14	22.6, CH_3	1.24, d (6.9)	2β	3, 2, 1	3α	22.47, CH_3	
15	199.5, CH	9.79, s		8, 10, 9		197.49, CH	9.70, s
1'	180.9, C						
2'	162.4, C						
3'	114.4, C						
4'	180.7, C						
5'	157.6, C						
6'	114.4, C						
1"	131.4, C					131.47, C	
2"	28.5, CH	β: 3.36, m	14", 3"β	4", 14", 3", 1", 6", 9"		28.39, CH	
3"	26.5, CH_2	α: 1.48, m<br>β: 2.09, m	4"β<br>4"β, 2"β		14", 5"α	26.30, CH_2	
4"	20.2, CH_2	α: 1.79, m<br>β: 2.00, m	 3"α, 3"β			19.79, CH_2	
5"	41.5, CH	α: 2.75, m	11"	11", 7", 6"	3"α	40.39, CH	α: 2.68, m
6"	132.6, C					132.66, C	
7"	117.5, C					117.40, C	
8"	119.9, C					119.71, C	
9"	146.0, C					146.04, C	
10"	138.3, C					138.42, C	
11"	34.3, CH	1.66, sptd (6.9, 6.9)	13", 12", 5"α	12", 13", 5", 6"		34.77, CH	
12"	20.4, CH_3	0.69, d (6.9)	11"	13", 11", 5"			
13"	22.2, CH_3	0.67, d (6.9)	11"	12", 11", 5"			
14"	23.0, CH_3	1.21, d (6.9)	2"β	3", 2", 1"	3"α	23.04, CH_3	
15"	67.2, CH_2	4.73, d (12.9)<br>5.65, d (12.9)	15"<br>15"	7", 8", 6", 10", 9", 5'<br>7", 8", 10", 9", 5'		67.37, CH_2	4.67, d (12.9)<br>5.68, d (12.9)

[a] deviating ^{1}H/^{13}C shifts of minor isomers

Table S7. NMR Data (^{1}H, 700 MHz) of compound **3** in Acetone-d_6 (δ in ppm)

No.	δ_C* , type	δ_H (J in Hz)	COSY	C to H HMBC
1	128.9, C			
2	27.5, CH	3.28, m	14	13, 14, 3, 6, 1, 10
3	26.3, CH_2	1.45, m<br>2.08, S	4, 3<br>3, 4	
4	19.9, CH_2	1.77, m<br>1.99, m	3, 3, 5<br>5	3, 5
5	41.2, CH	2.74, m	11, 4, 4	
6	124.6, C			
7	118.4, C			
8	122.2, C			
9	142.2, C			
9OH				
10	143.7, C			
11	34.0, CH	1.63, m	12, 13, 5	13, 12, 5
12	21.8, CH_3	0.65, d (6.9)	11	13, 11, 5
13	20.1, CH_3	0.68, d (6.9)	11	12, 11, 5
14	22.6, CH_3	1.21, d (6.9)	2	3, 1
15	62.5, CH_2	4.55, d (13.8)<br>4.82, d (13.8)	15<br>15	7, 8, 9<br>7, 8, 9
1'	-	-		
2'	-	-		
3'	-	-		
4'	-	-		
5'	157.0, C			
6'	117.2, C			
1"	131.0, C			
2"	28.1, CH	3.36, m	14"	4", 14", 3", 6", 1", 7", 10"
3"	26.3, CH_2	1.45, m<br>2.08, S	4", 3"<br>3", 4"	
4"	19.9, CH_2	1.77, m<br>1.99, m	3", 3"	3"
5"	41.7, CH	2.24, m		12", 11", 7"
6"	126.7, C			
7"	132.1, C			
8"	119.5, C			
9"	138.0, C			
9"OH				
10"	145.6, C			
11"	33.3, CH	1.85, m	12", 13"	12", 13", 5"
12"	20.7, CH_3	0.72, d (6.9)	11"	13", 11", 5"
13"	22.2, CH_3	0.83, d (6.9)	11"	12", 11", 5"
14"	22.6, CH_3	1.21, d (6.9)	2"	3", 2", 1"
15"	66.9, CH_2	4.73, d (13.1)<br>5.64, d (13.1)	15"<br>15"	6', 8", 7", 9", 10", 5'<br>6', 8", 9", 10", 5'

- ^{1}H/^{13}C chemical shifts not shown due to absence of, * ^{13}C chemical shifts were extracted from the HMBC NMR spectrum, since compound 3 was degraded after the measurement of the 1H, COSY, HSQC and HMBC NMR data.

Table S8. NMR Data (^{1}H, 500 MHz, ^{13}C 175 MHz) of compound **4** in Acetone-d_6 (δ in ppm)

No.	δ_C , type	δ_H (J in Hz)	COSY	C to H HMBC	N/ROESY
1	131.4, C				
2	28.4, CH	β: 3.35, m	14, 3α, 3β	4, 14, 3, 1, 6, 9	4β, 3β
3	26.8, CH_2	α:1.49, m<br>β: 1.99, m	4α, 3β, 2β<br>3α, 2β	5, 1<br>4, 14	14<br>2β
4	19.7, CH_2	α: 1.70, m<br>β: 1.96, m	3α, 4β<br>4α, 5α	3<br>3, 11, 5	5α<br>2β
5	40.3, CH	α: 2.81, m	11, 4β	4, 3, 11, 7, 1, 6	12, 4α
6	132.5, C				
7	117.8, C				
8	120.3, C				
9	145.8, C				
10	138.2, C				
11	34.6, CH	1.70, m	12, 5α	13, 12, 5, 6	
12	22.0, CH_3	0.64, d (6.7)	11	13, 11, 5	5α
13	20.0, CH_3	0.61, d (6.7)		12, 11, 5	
14	22.7, CH_3	1.21, d (7.0)	2β	3, 2, 1	3α
15	67.0, CH_2	4.68, d (13.0)<br>5.64, d (13.0)	15<br>15	7, 8, 6, 10, 9, 2'<br>7, 8, 10, 9, 2'	
1'	178.5, C				
2'	154.2, C				
3'	125.1, C				
4'	178.5, C				
5'	154.2, C				
6'	125.1, C				
1"	131.4, C				
2"	28.4, CH	β: 3.35, m	14", 3"α, 3"β	4", 14", 3", 1", 6", 9"	4"β, 3"β
3"	26.8, CH_2	α:1.49, m<br>β: 1.99, m	4"α, 3"β, 2"β<br>3"α, 2"β	5", 1"<br>4", 14"	14"<br>2"β
4"	19.7, CH_2	α: 1.70, m<br>β: 1.96, m	3"α, 4"β<br>4"α, 5"α	3"<br>3", 11", 5"	5"α<br>2"β
5"	40.3, CH	α: 2.81, m	11", 4"β	4", 3", 11", 7", 1", 6"	12", 4"α
6"	132.5, C				
7"	117.8, C				
8"	120.3, C				
9"	145.8, C				
10"	138.2, C				
11"	34.6, CH	1.70, m	12", 5"α	13", 12", 5", 6"	
12"	22.0, CH_3	0.64, d (6.7)	11"	13", 11", 5"	5"α
13"	20.0, CH_3	0.61, d (6.7)		12", 11", 5"	
14"	22.7, CH_3	1.21, d (7.0)	2"β	3", 2", 1"	3"α
15"	67.0, CH_2	4.68, d (13.0)<br>5.64, d (13.0)	15"<br>15"	7", 8", 6", 10", 9", 5'<br>7", 8", 10", 9", 5'	

Table S9. NMR Data (^{1}H, 700 MHz, ^{13}C 175 MHz) of compound **5** in Acetone-d_6 (δ in ppm)

No.	δ_C , type	δ_H (J in Hz)	COSY	C to H HMBC	N/ROESY
1	139.0, C				
2	28.6, CH	β: 3.39, m	14, 3β	4, 14, 3, 6, 1, 10	3β
3	26.4, CH_2	α: 1.46, m<br>β: 2.07, m	4, 3β<br>3α, 2β		14<br>2β
4	20.0, CH_2	1.80, m	3α, 5α		12, 14
5	40.8, CH	α: 2.51, m	11, 4		
6	133.4, C				
7	126.5, C				
8	117.4, C				
9	148.3, C				
9OH		11.84, s		8, 10, 9	
10	142.8, C				
11	34.3, CH	1.70, m	13, 12, 5α	4, 13, 12, 5, 6	
12	22.2, CH_3	0.71, d (6.9)	11	13, 11, 5	4
13	20.7, CH_3	0.69, d (6.9)	11	12, 11, 5	
14	22.4, CH_3	1.19, m	2β	3, 2, 1	3α, 4
15	199.6, CH	9.76, s		8, 10, 9	
1'	183.6, C				
2'	146.7, C				
2'OH		6.83, s		3', 7, 2', 1'	
3'	119.6, C				
4'	183.5, C				
5'	146.7, C				
5'OH		6.83, s		5'	
6'	112.5, C				
7'	7.8, CH_3	1.90, s		6'	

Table S10. NMR Data (^{1}H, 500 MHz, ^{13}C 125 MHz) of compound **6** in Acetone-d_6 (δ in ppm)

No.	δ_C , type	δ_H (J in Hz)	COSY	C to H HMBC	N/ROESY
1	139.2, C				
2	28.6, CH	3.41, dq (6.8, 6.8)	14, 3β	4, 14, 3, 6, 1, 10	4, 3β
3	26.3, CH_2	α: 1.48, m<br>β: 2.08, m	4, 3β<br>3α, 4, 2β	4, 14, 5, 1<br>4, 14	14<br>2β
4	19.9, CH_2	1.83, m	3α, 3β, 5α	3, 11	2β
5	41.0, CH	α: 2.46, br s	11, 4		
6	133.3, C				
7	124.7, C				
8	117.2, C				
9	148.6, C				
9OH		11.86		8, 10, 9	
10	143.6, C				
11	34.4, CH	1.67, sptd (6.9, 6.9)	12, 13, 5α	13, 5, 6	
12	20.6, CH_3	0.71, d (6.9)	11	13, 11, 5	
13	22.1, CH_3	0.75, d (6.9)	11	12, 11, 5	
14	22.3, CH_3	1.21, d (6.8)	2β	3, 2, 1	3α
15	199.2, CH	9.82, s		8, 10, 9	
1'	-	-			
2'	-	-			
2'OH	-	-			
3'	114.0, C				
4'	-	-			
5'	-	-			
5'OH	-	-			
6'	104.9, CH	6.09, s			
7'	-	-			

- ^{1}H/^{13}C chemical shifts not shown due to absence of signals

Table S11. NMR Data (^{1}H, 500 MHz, ^{13}C 125 MHz) of compound **7** in Acetone-d_6 (δ in ppm)

No.	δ_C , type	δ_H (J in Hz)	COSY	C to H HMBC	N/ROESY
1	144.8, C				
2	35.1, CH	β: 2.32, m	14, 3β, 3α		3β
3	30.7, CH_2	α: 1.88, m<br>β: 1.22, m	3β, 4β, 4α, 2β<br>4β, 4α, 3α, 2β	14, 4, 2, 5, 1<br>4, 2, 1	14<br>2β
4	21.4, CH_2	α: 1.69, m<br>β: 1.46, m	3β, 4β, 3α, 11, 5α<br>3β, 4α, 3α, 5α	11, 5<br>3, 5, 6	14, 5α
5	43.6, CH	α: 2.14, m	4β, 4α		4α
6	131.3, C				
7	138.2, CH	7.04, d (2.4)		9, 5, 8, 6, 1, 15	
8	125.5, C				
9	22.4, CH_2	2.56, m	10	10, 8, 6, 7, 1, 15	
10	28.1, CH_2	2.18, m	9	9, 8, 6	
11	30.9, CH	1.97, m	13, 12, 4α	13, 12, 4, 5, 6	
12	21.3, CH_3	0.96, d (6.9)	11	13, 11, 5	
13	17.7, CH_3	0.73, d (6.9)	11	12, 11, 5	
14	18.9, CH_3	1.01, d (7.0)	2β	3, 2, 1	4α, 3α
15	168.5, C				

Table S12. NMR Data (^{1}H, 500 MHz, ^{13}C 125 MHz) of compound **8** in MeOH-d_4 (δ in ppm)

No.	δ_C , type	δ_H (J in Hz)	COSY	C to H HMBC
1	130.6, C			
2	121.4, C			
3	146.8, C			
4	142.0, C			
5	132.6, C			
6	124.0, C			
7	136.7, CH	7.98, s		9, 6, 8, 1, 5, 15
8	126.6, C			
9	22.7, CH_2	2.43, br dd (8.2, 8.2)	10	10, 8, 1, 7, 15
10	26.2, CH_2	2.70, br dd (8.2, 8.2)	9	9, 2, 6, 8, 1, 3
11	28.5, CH	3.53, qq (7.2, 7.2)	12, 13	12, 13, 6, 5
12	22.1, CH_3†	1.39, d (7.2)†	11	11, 5
13	22.1, CH_3†	1.39, d (7.2)†	11	11, 5
14	12.1, CH_3	2.18, s		2, 6, 1, 4, 3
15	171.7, C			

† overlapped

Table S13. NMR Data (^{1}H, 700 MHz, ^{13}C 175 MHz) of compound **11** in DMSO-d_6 (δ in ppm)

No.	δ_C , type	δ_H (J in Hz)	COSY	C to H HMBC	N/ROESY
1	142.0, C				
2	29.0, CH	3.41, m	14		14
3	25.9, CH_2	1.50, m<br>2.05, m			14<br>11
4	19.1, CH_2	1.63, m<br>2.02, m	 5	6	12<br>5
5	41.4, CH	3.00, m	11, 4	6	13, 11, 4, 5'
6	136.2, C				
7	123.8, C				
8	124.3, C				
9	138.6, C				
9OMe	62.3, CH_3	3.88, s		9	15, 10OH
10	148.1, C				
10OH		10.21, s		9, 1, 10	14, 9OMe
11	32.7, CH	1.45, m	13, 12, 5	12, 13, 5, 6	13, 12, 3, 5, 5'
12	18.6, CH_3	0.38, d (6.7)	11	13, 11, 5	11, 4
13	21.5, CH_3	0.30, d (6.7)	11	12, 11, 5	11, 5, 5'
14	22.8, CH_3	1.25, m	2	3, 2, 1	3, 2, 10OH
15	149.9, CH	9.46, s		7, 8, 9, 2'	9OMe
1'	183.7, C				
2'	141.5, C				
3'	129.3, C				
4'	187.7, C				
5'	135.0, CH	7.08, s	7'	7', 3', 6', 1'	13, 11, 7', 5
6'	145.3, CH				
7'	15.4, CH_3	2.13, d (1.5)	5'	5', 6', 1'	5'

^{1}H NMR spectrum (500 MHz, acetone-d_6) of bis-heimiomycin A (**1**).

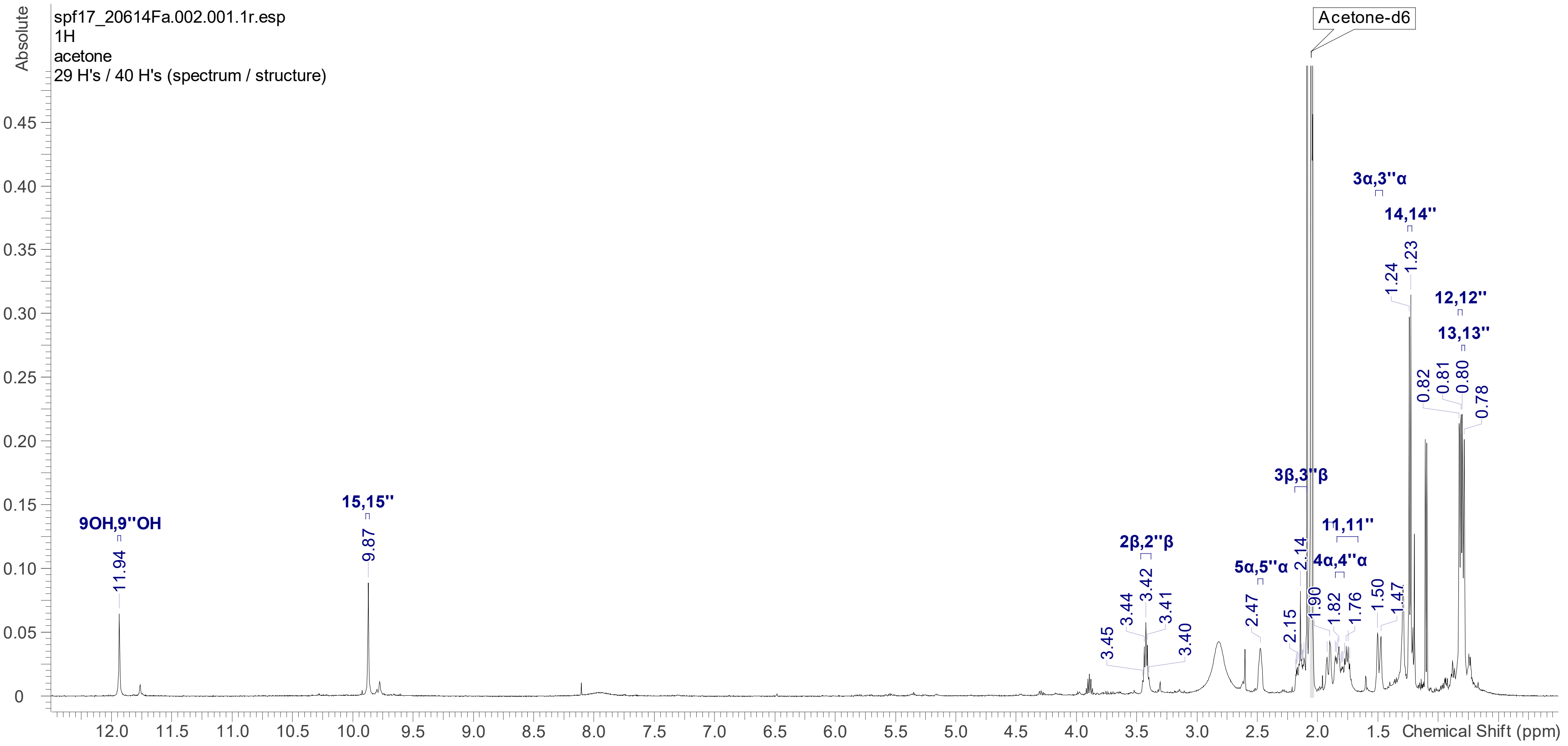

^{13}C NMR spectrum (175 MHz, acetone-d_6) of bis-heimiomycin A (**1**).

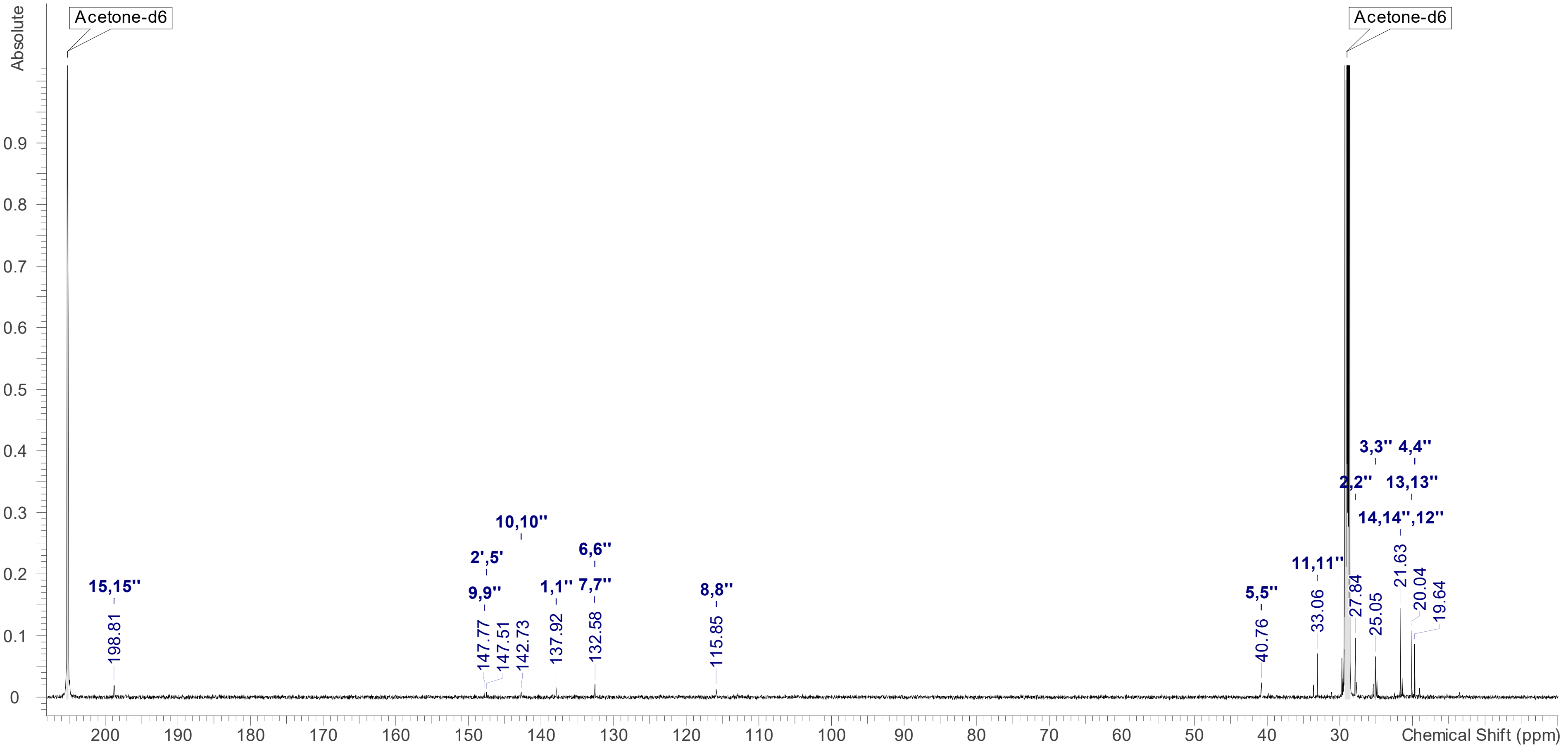

COSY NMR spectrum (500 MHz, acetone-d_6) of bis-heimiomycin A (**1**).

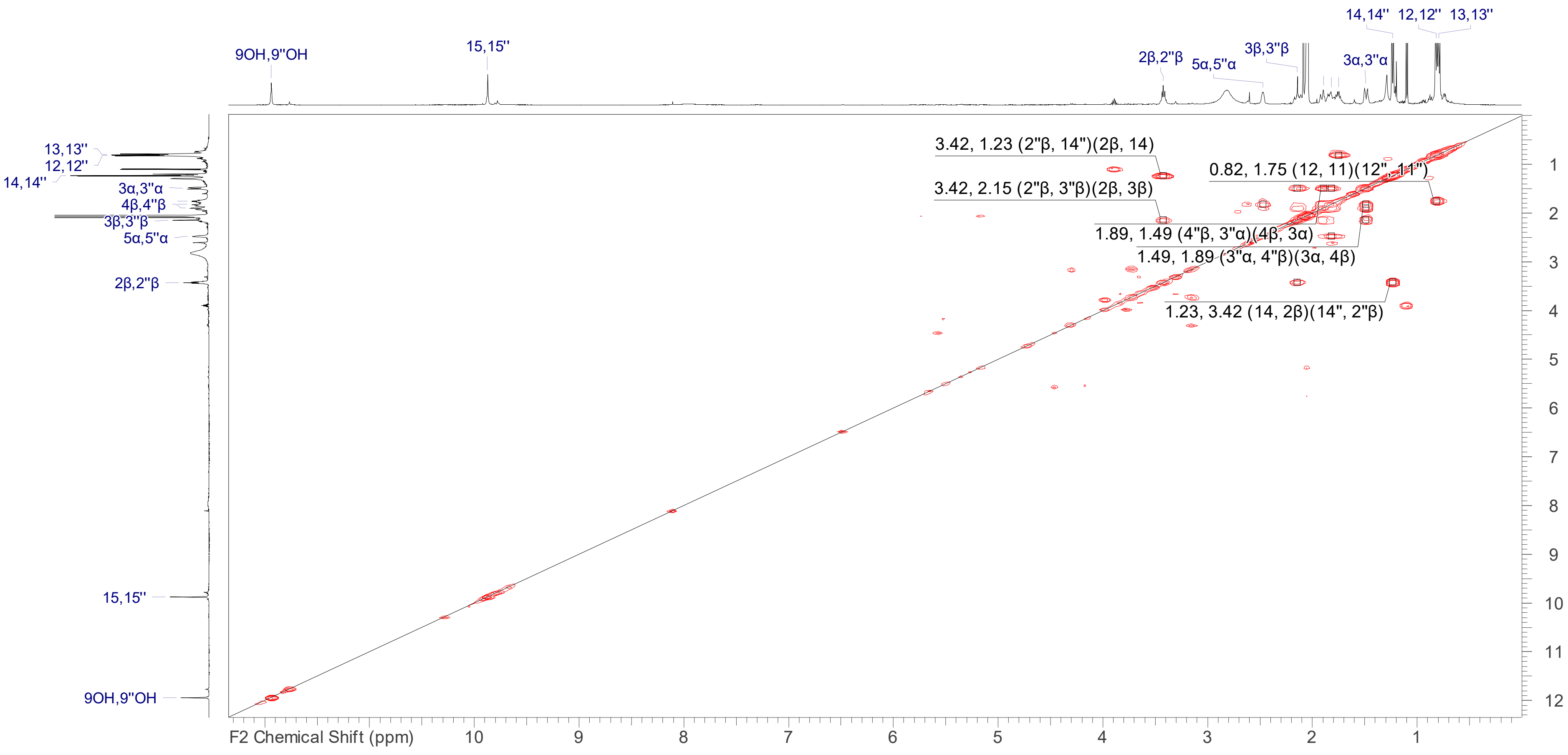

HSQC NMR spectrum (500 MHz, acetone-d_6) of bis-heimiomycin A (**1**).

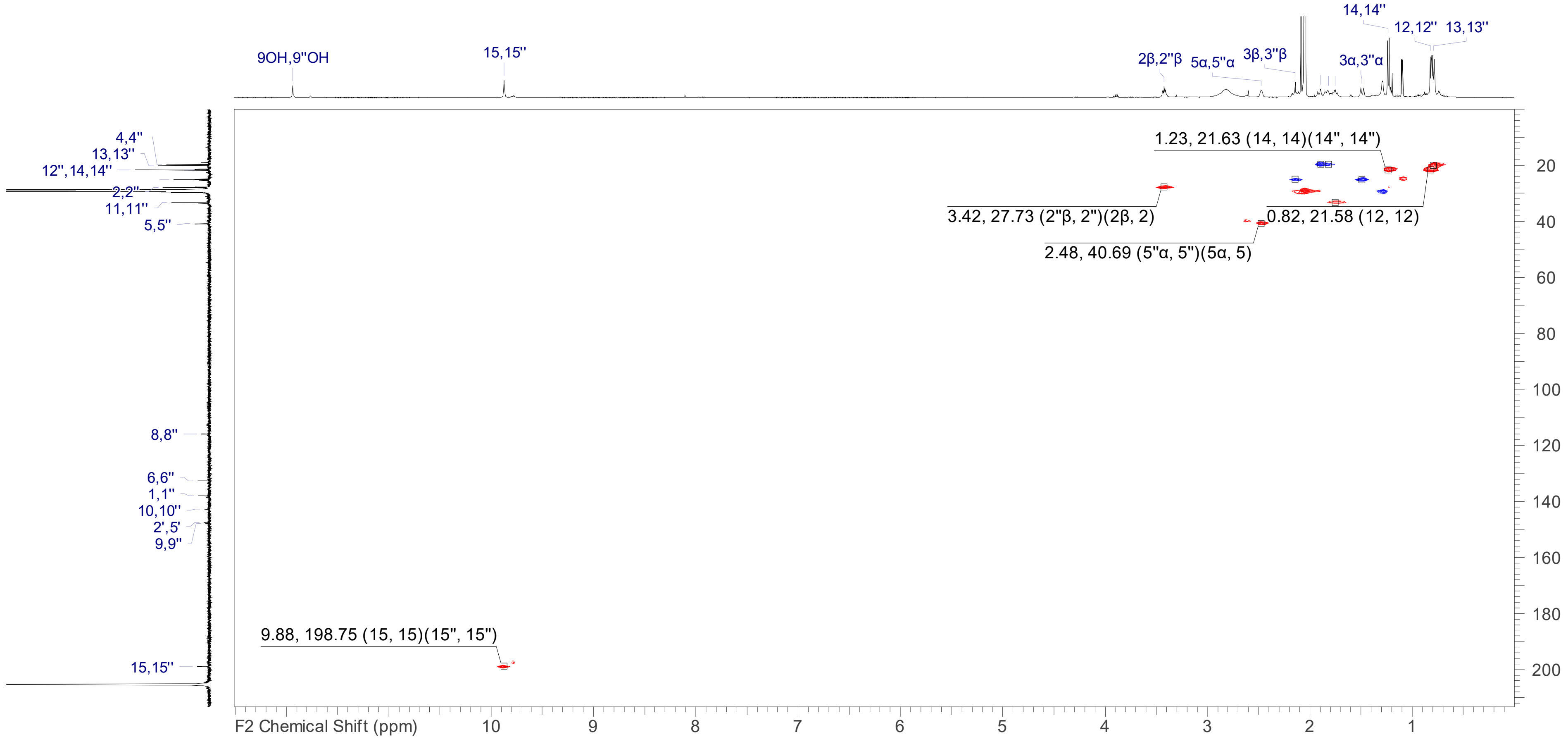

HMBC NMR spectrum (500 MHz, acetone-d_6) of bis-heimiomycin A (**1**).

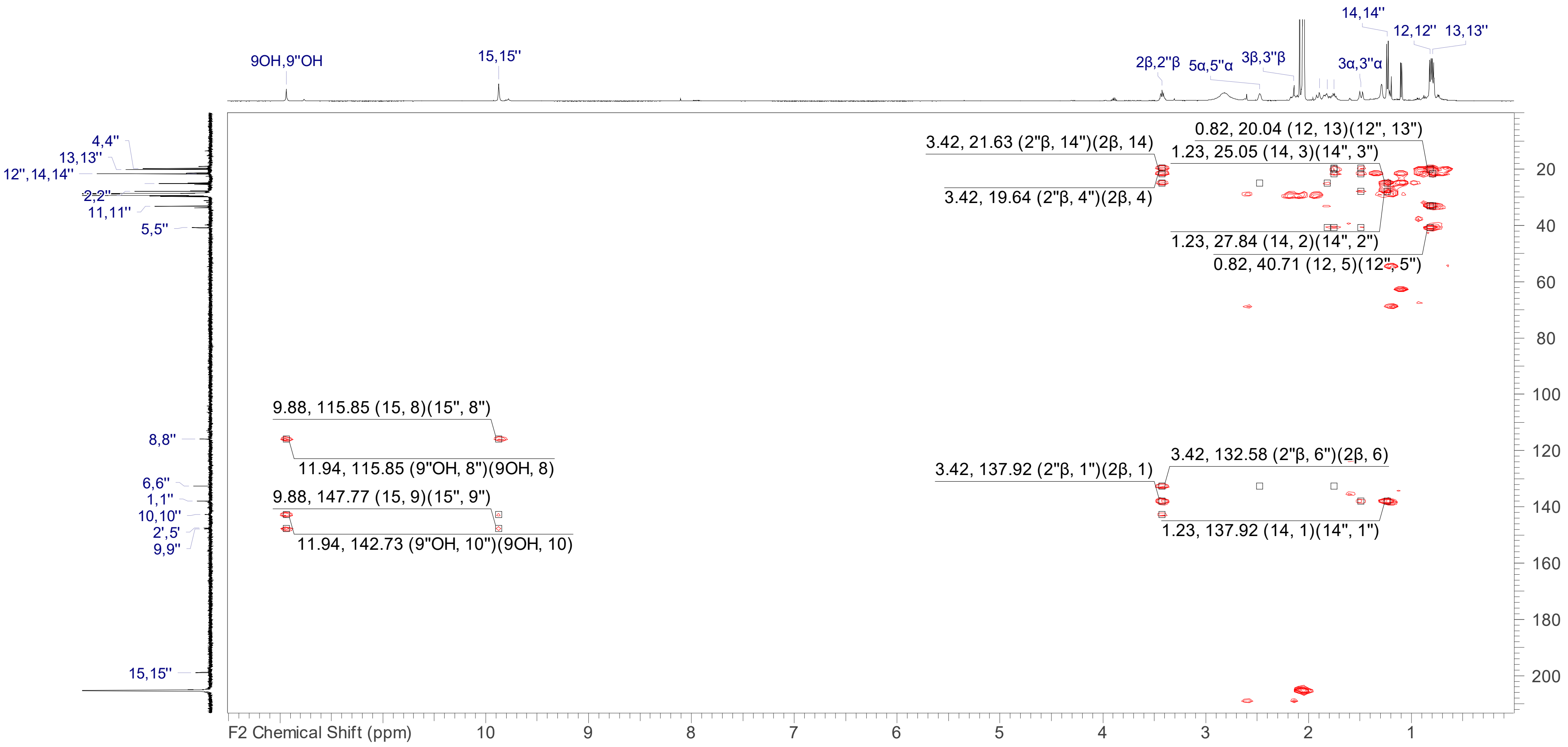

ROESY NMR spectrum (500 MHz, acetone-d_6) of bis-heimiomycin A (**1**).

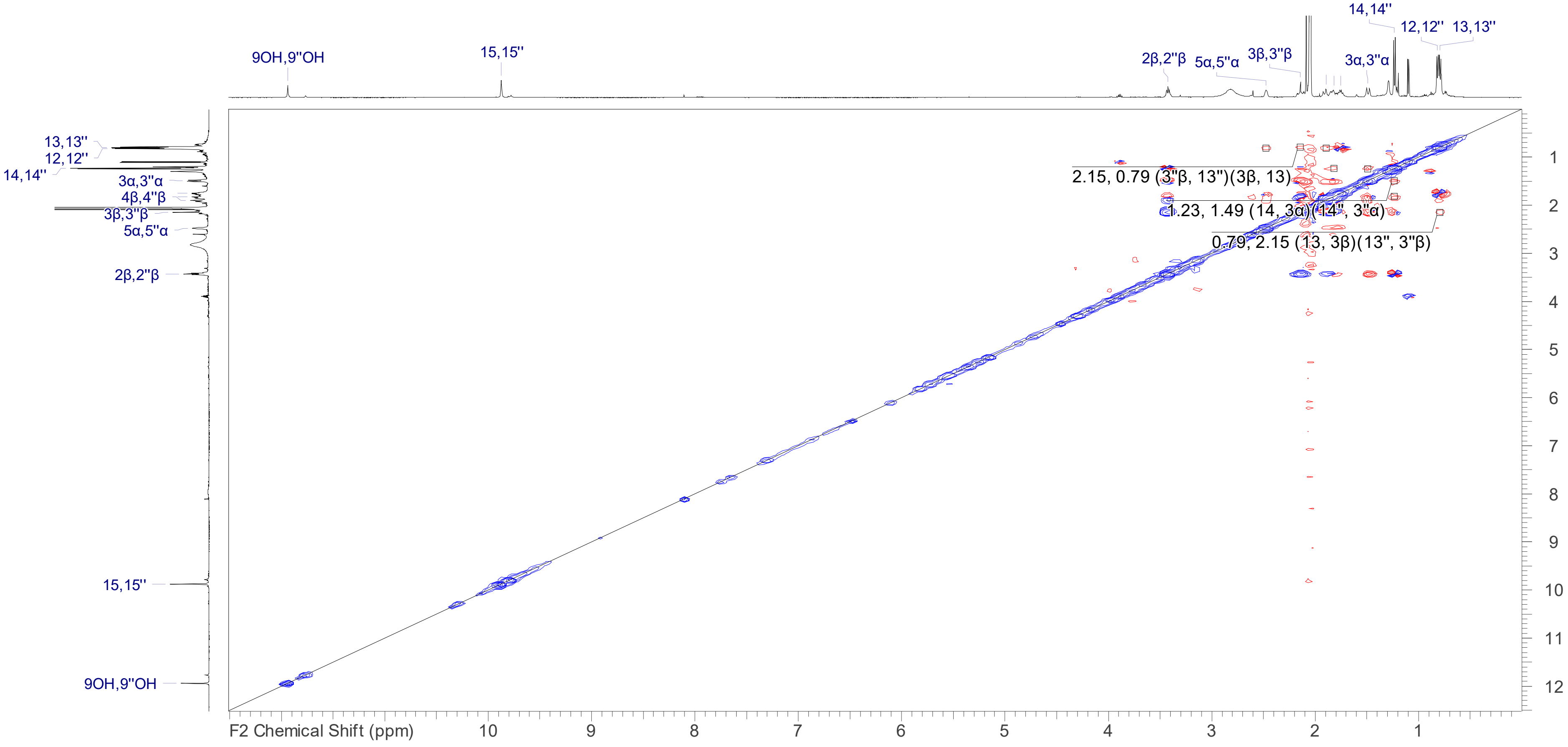

^{1}H NMR spectrum (700 MHz, acetone-d_6) of derivative of bis-heimiomycin A (**1b**).

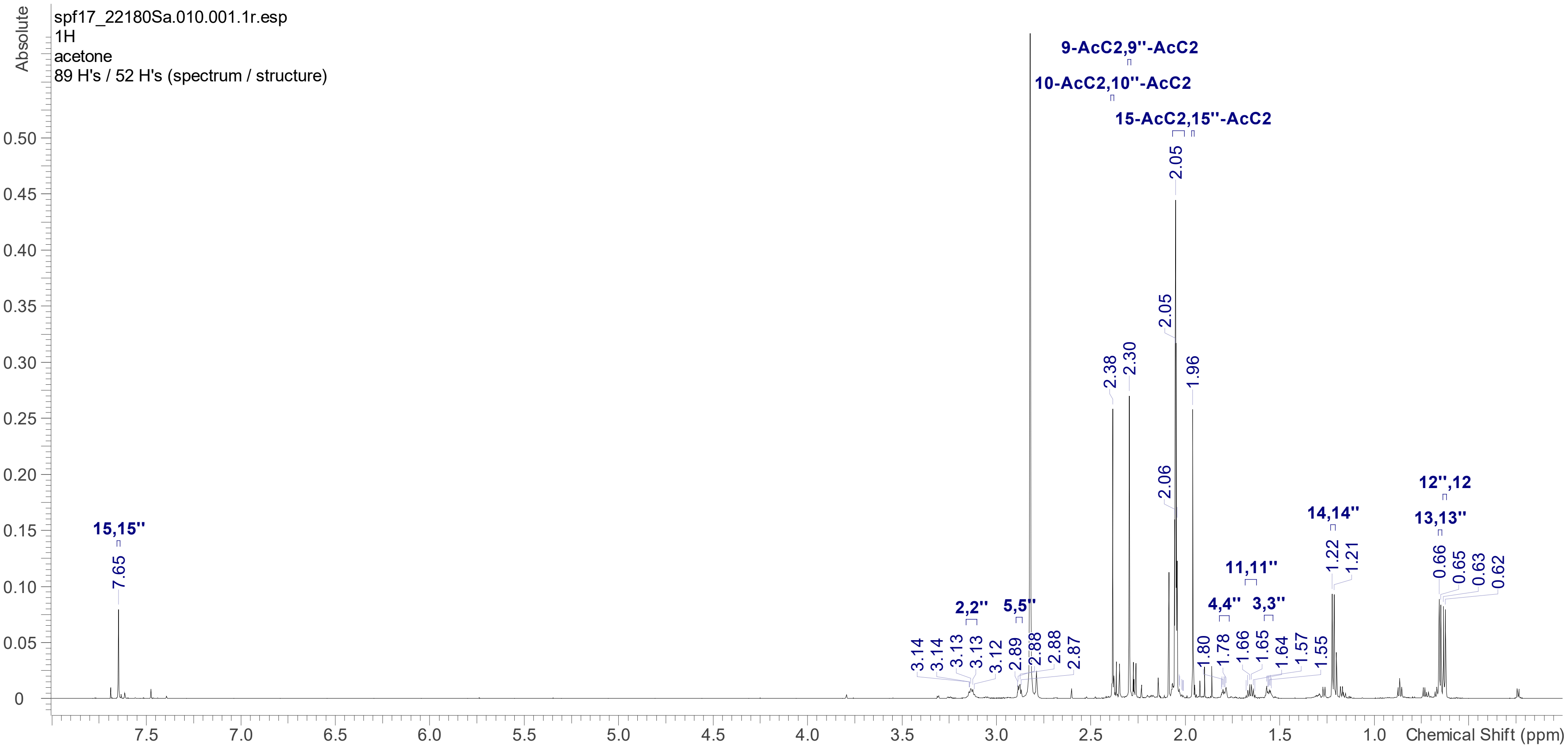

^{13}C NMR spectrum (175 MHz, acetone-d_6) of derivative of bis-heimiomycin A (**1b**).

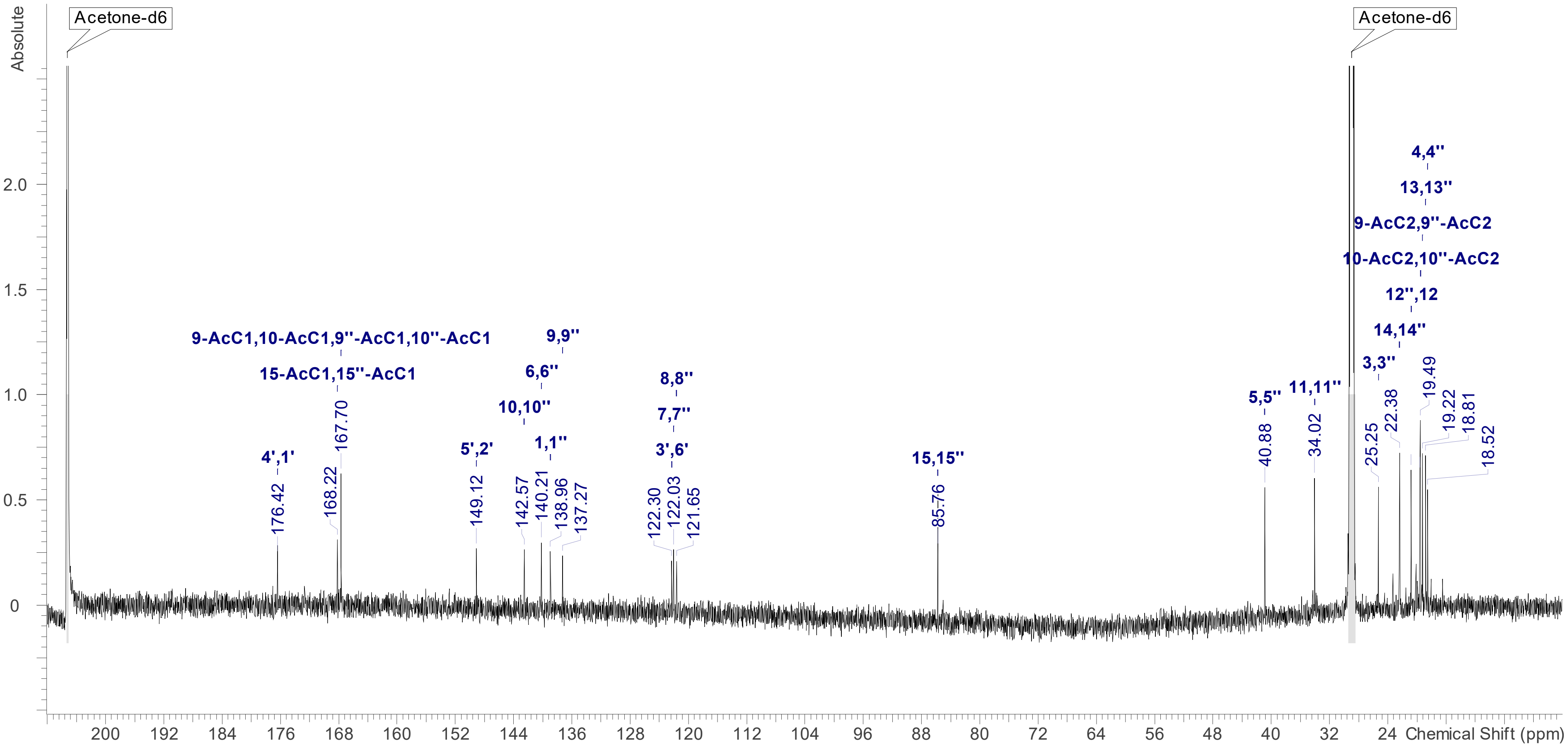

COSY NMR spectrum (700 MHz, acetone-d_6) of derivative of bis-heimiomycin A (**1b**).

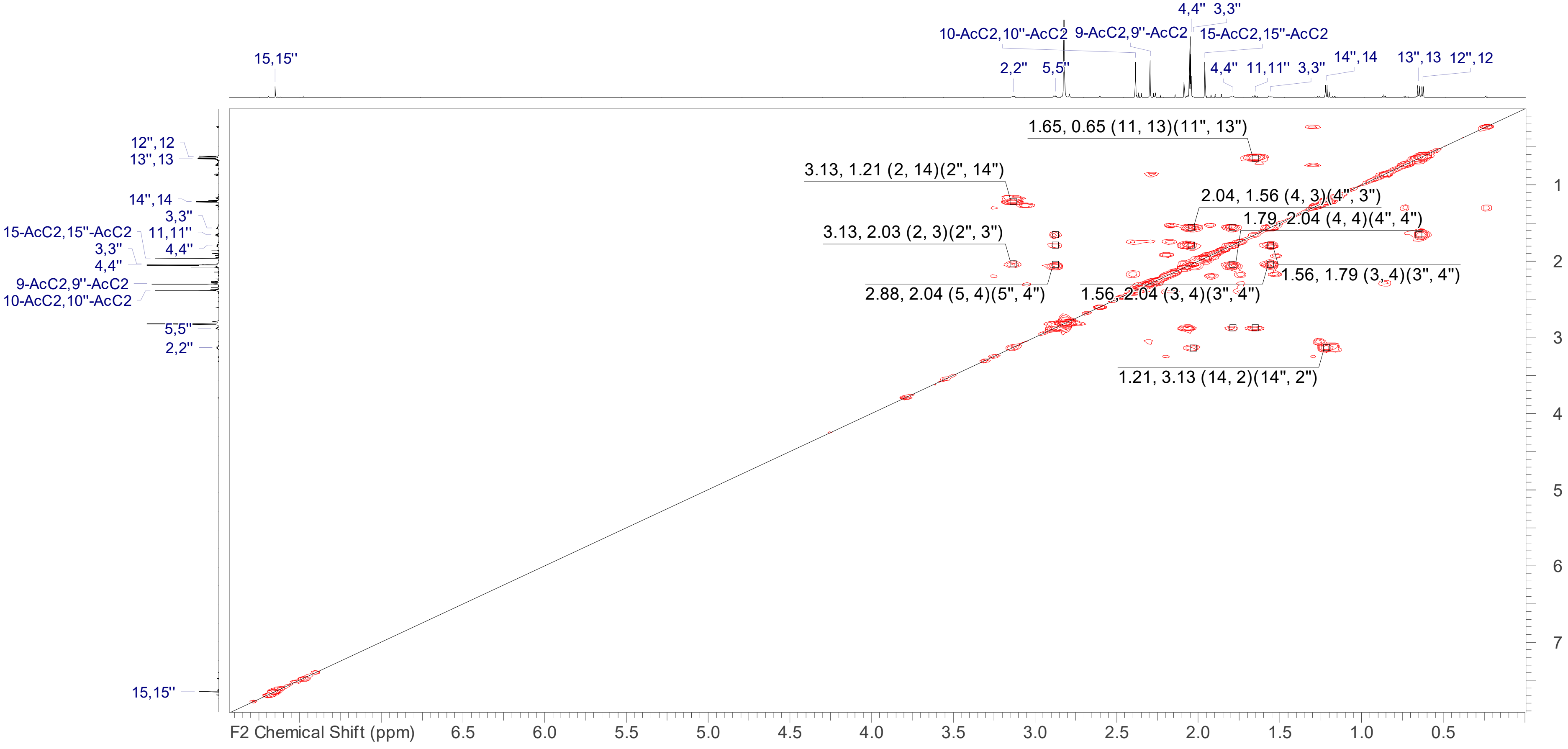

HSQC NMR spectrum (700 MHz, acetone-d_6) of derivative of bis-heimiomycin A (**1b**).

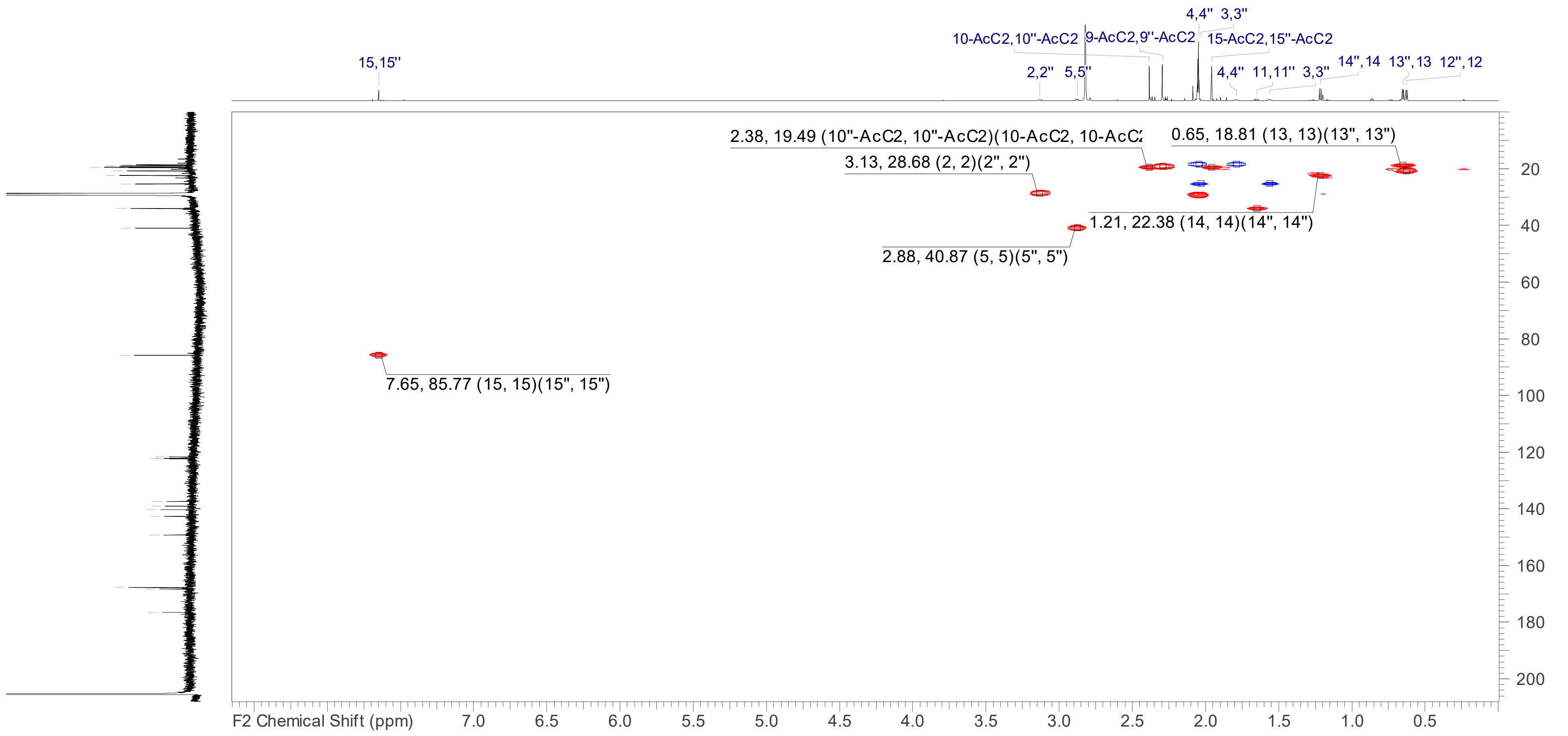

HMBC NMR spectrum (700 MHz, acetone-d_6) of derivative of bis-heimiomycin A (**1b**).

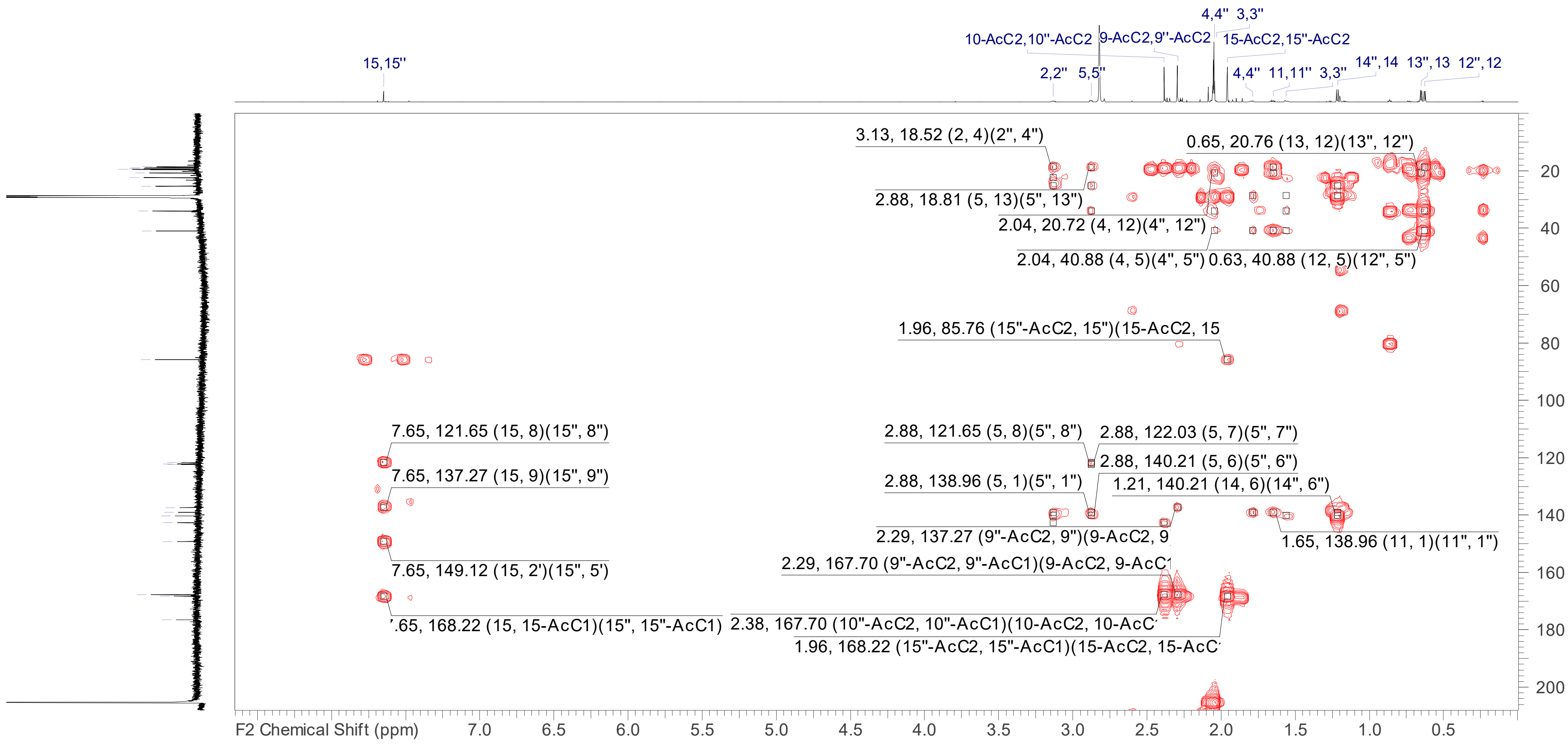

^{1}H NMR spectrum (700 MHz, acetone-d_6) of bis-heimiomycin B (**2**).

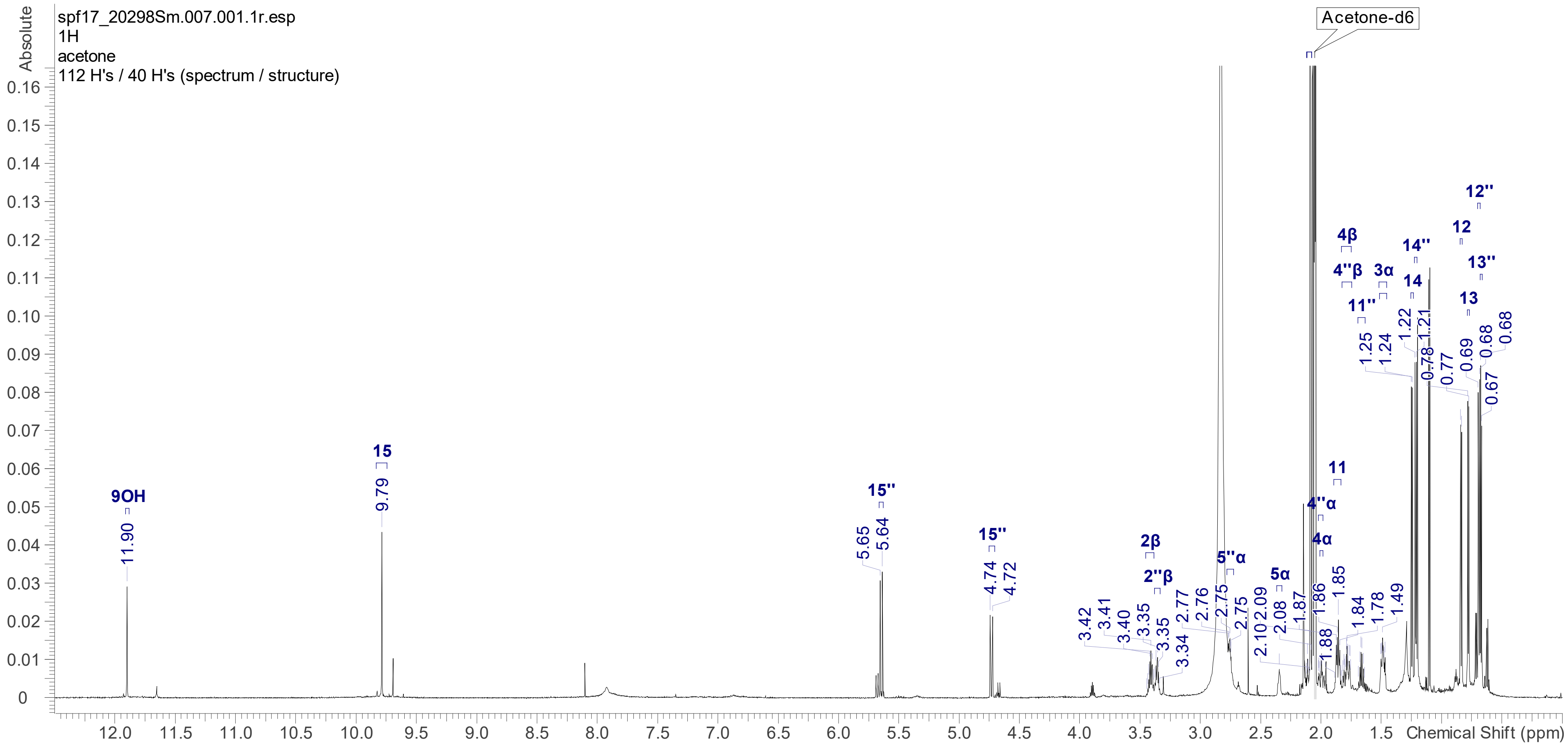

^{13}C NMR spectrum (175 MHz, acetone-d_6) of bis-heimiomycin B (**2**).

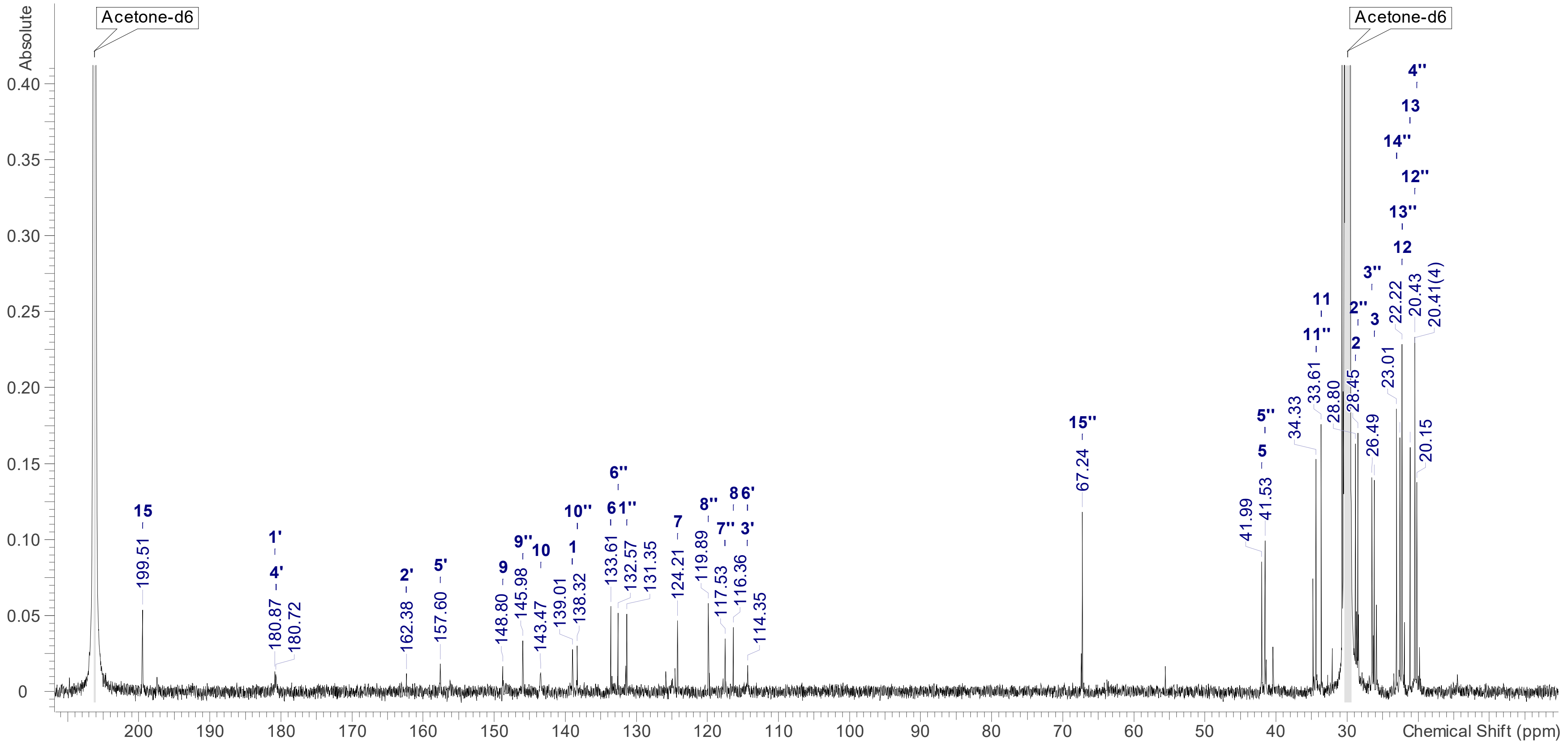

COSY NMR spectrum (500 MHz, acetone-d_6) of bis-heimiomycin B (**2**).

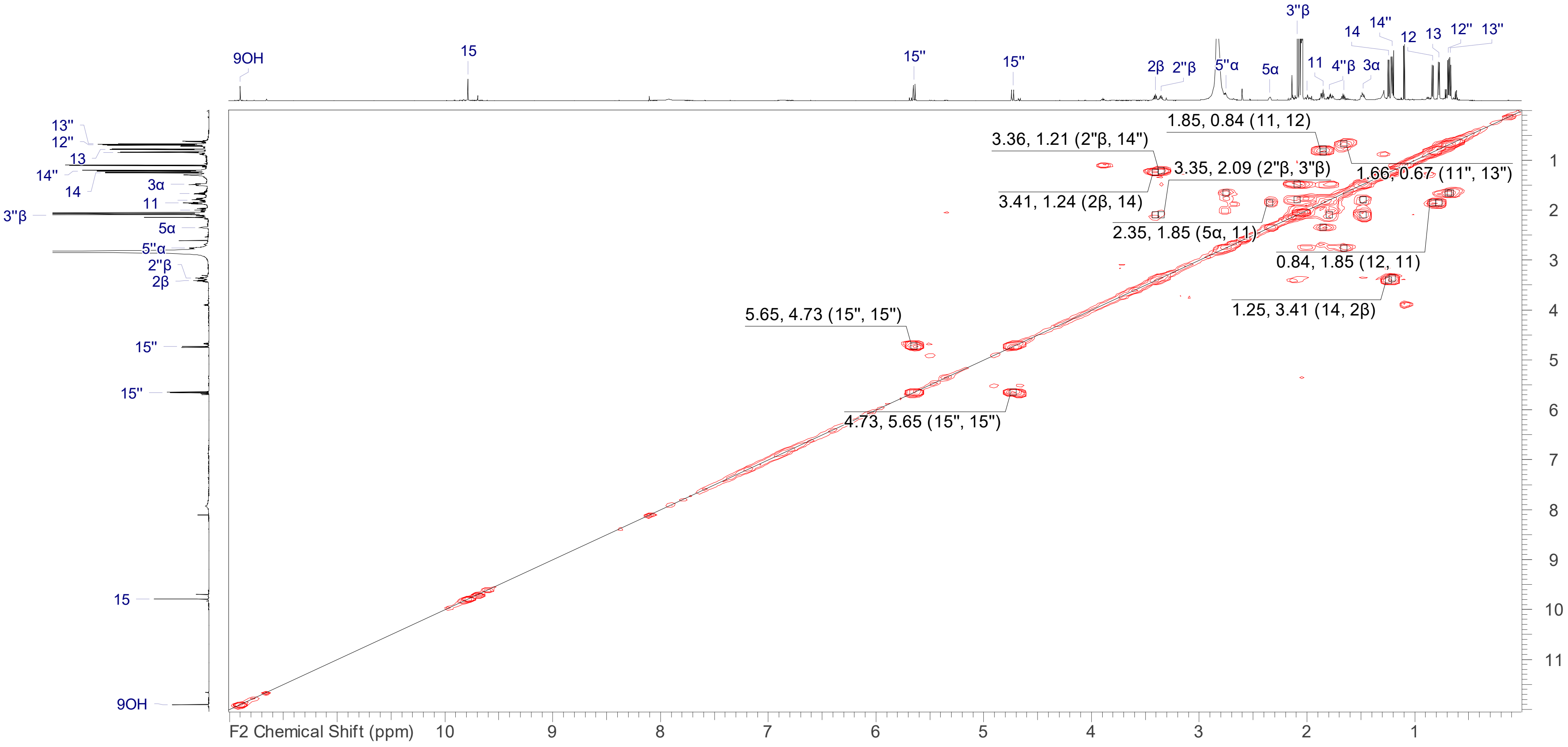

HSQC NMR spectrum (700 MHz, acetone-d_6) of bis-heimiomycin B (**2**).

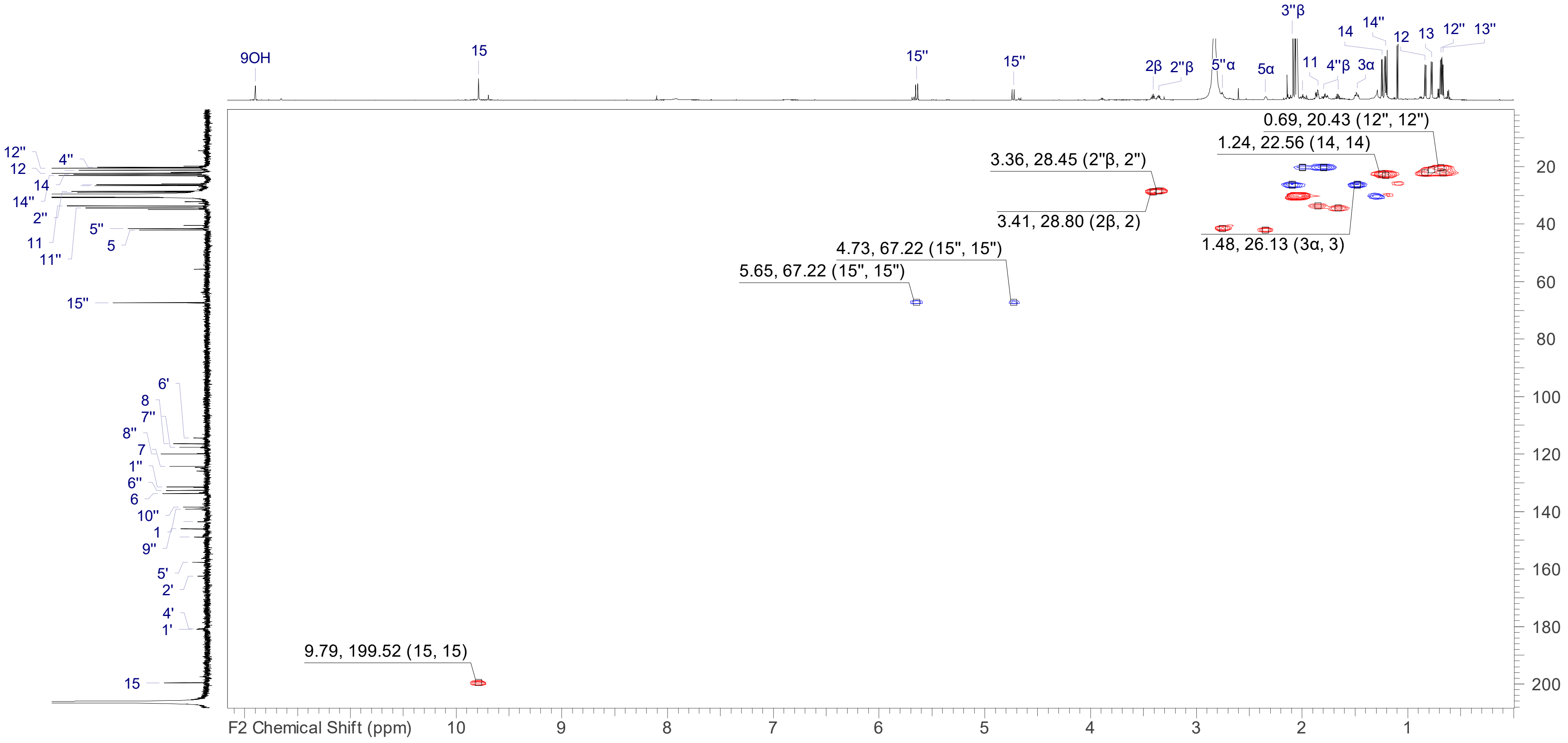

HMBC NMR spectrum (700 MHz, acetone-d_6) of bis-heimiomycin B (**2**).

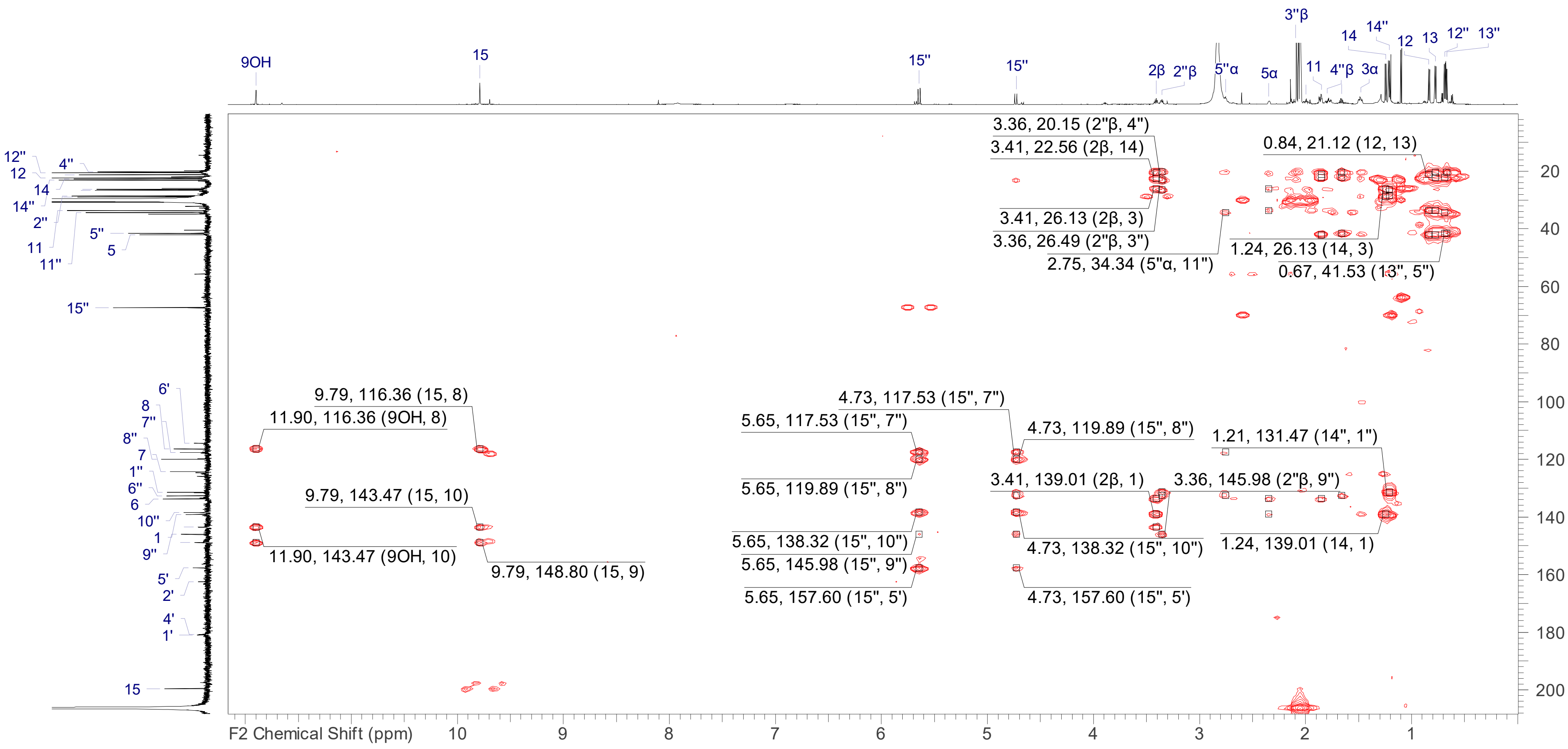

ROESY NMR spectrum (700 MHz, acetone-d_6) of bis-heimiomycin B (**2**).

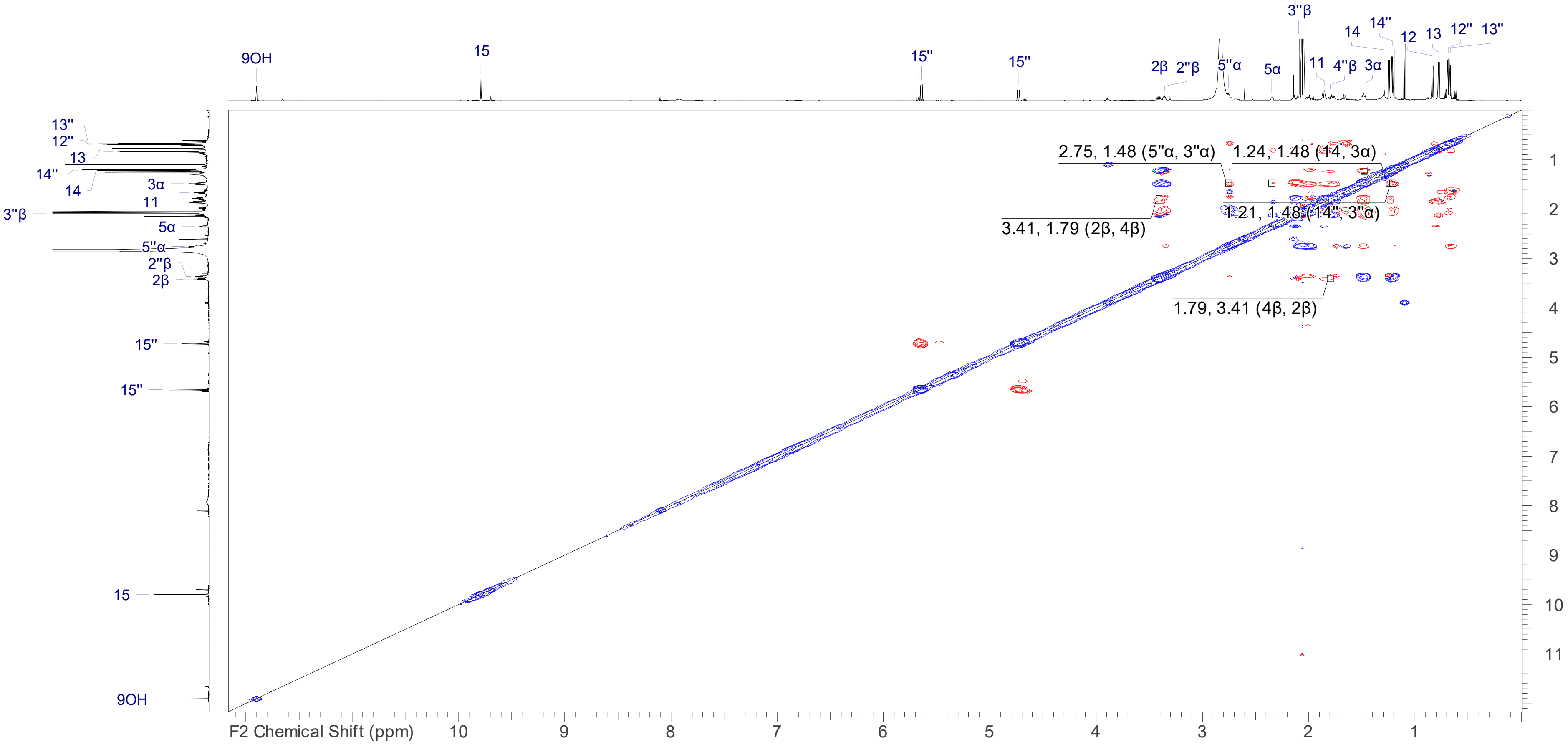

^{1}H NMR spectrum (700 MHz, acetone-d_6) of bis-heimiomycin C (**3**).

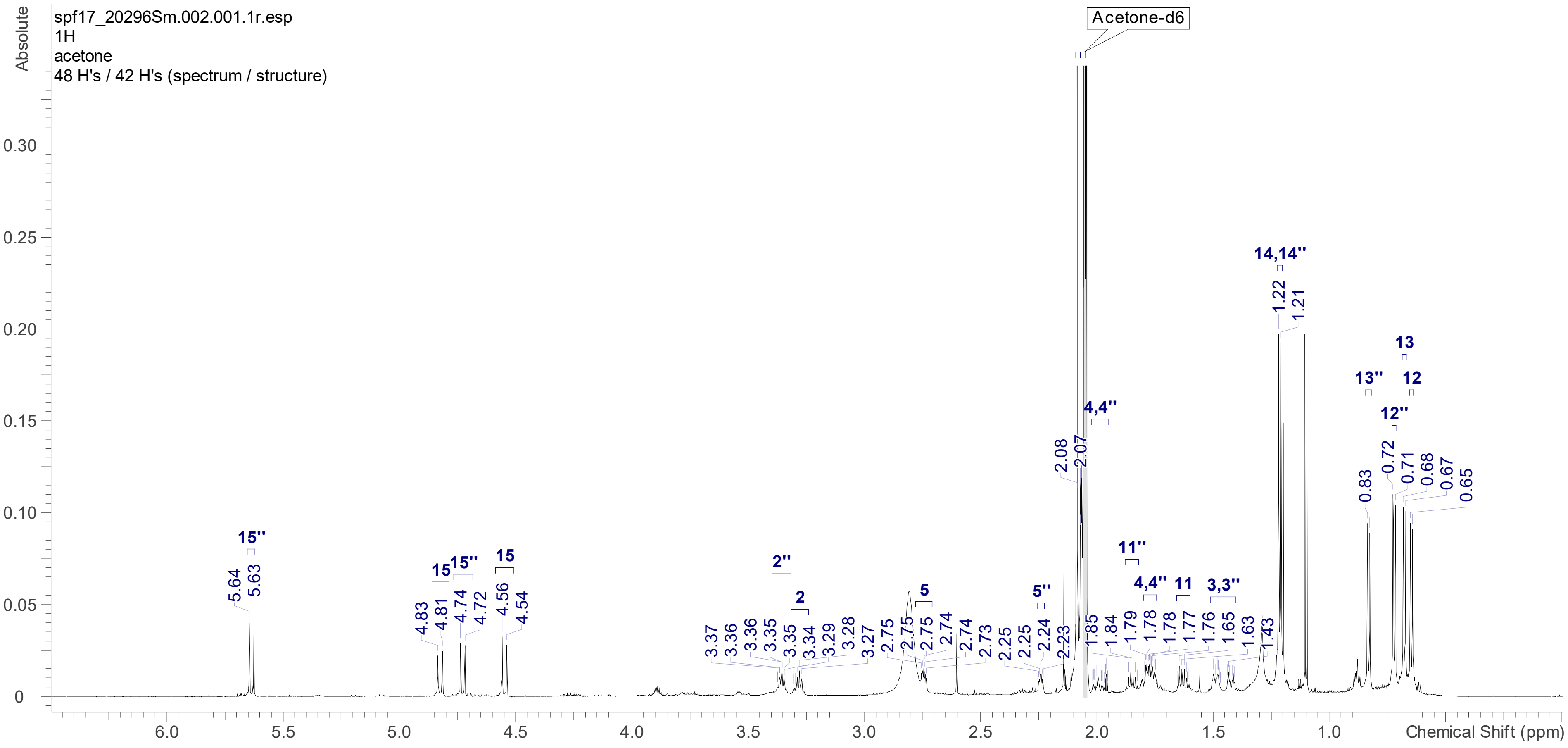

COSY NMR spectrum (700 MHz, acetone-d_6) of bis-heimiomycin C (**3**).

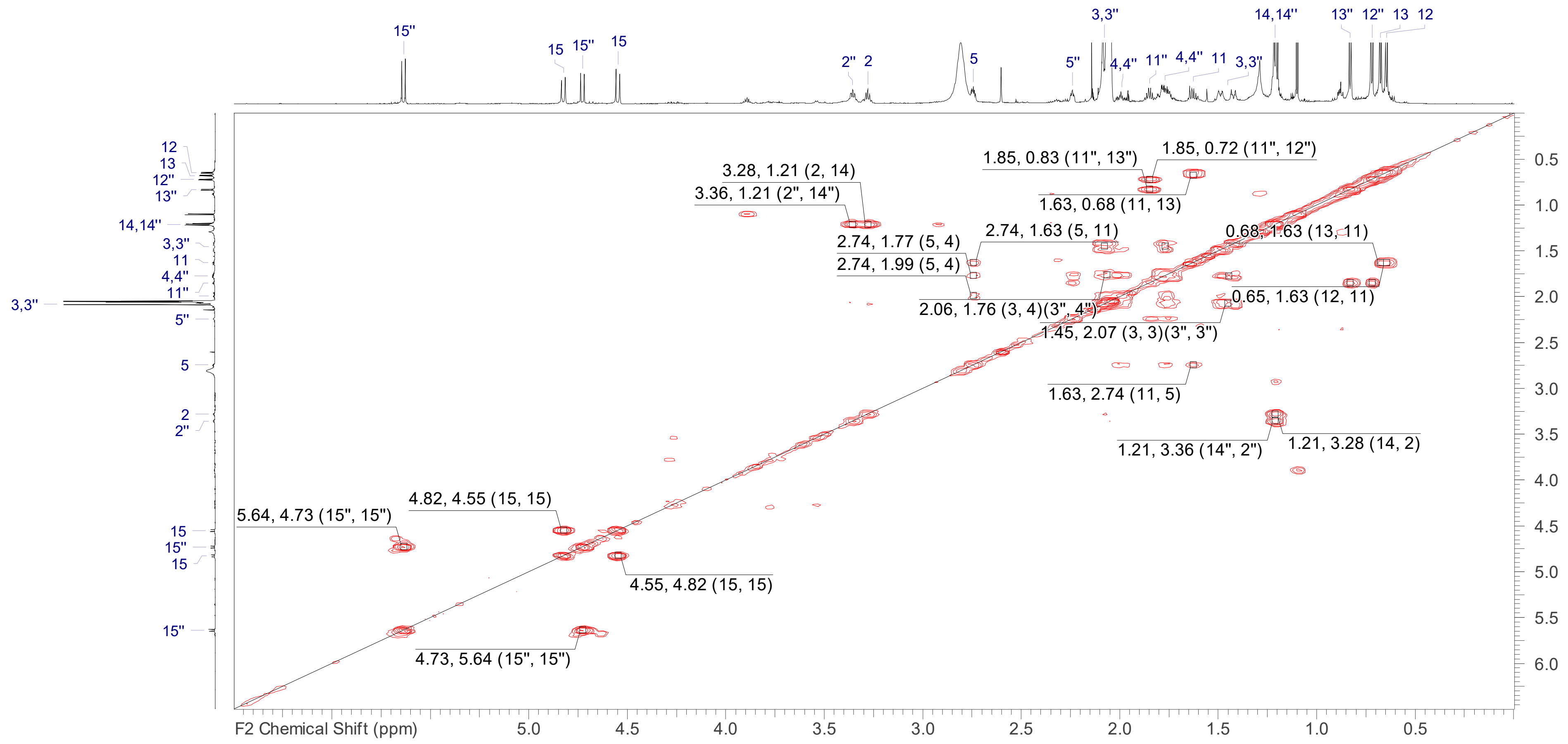

HSQC NMR spectrum (700 MHz, acetone-d_6) of bis-heimiomycin C (**3**).

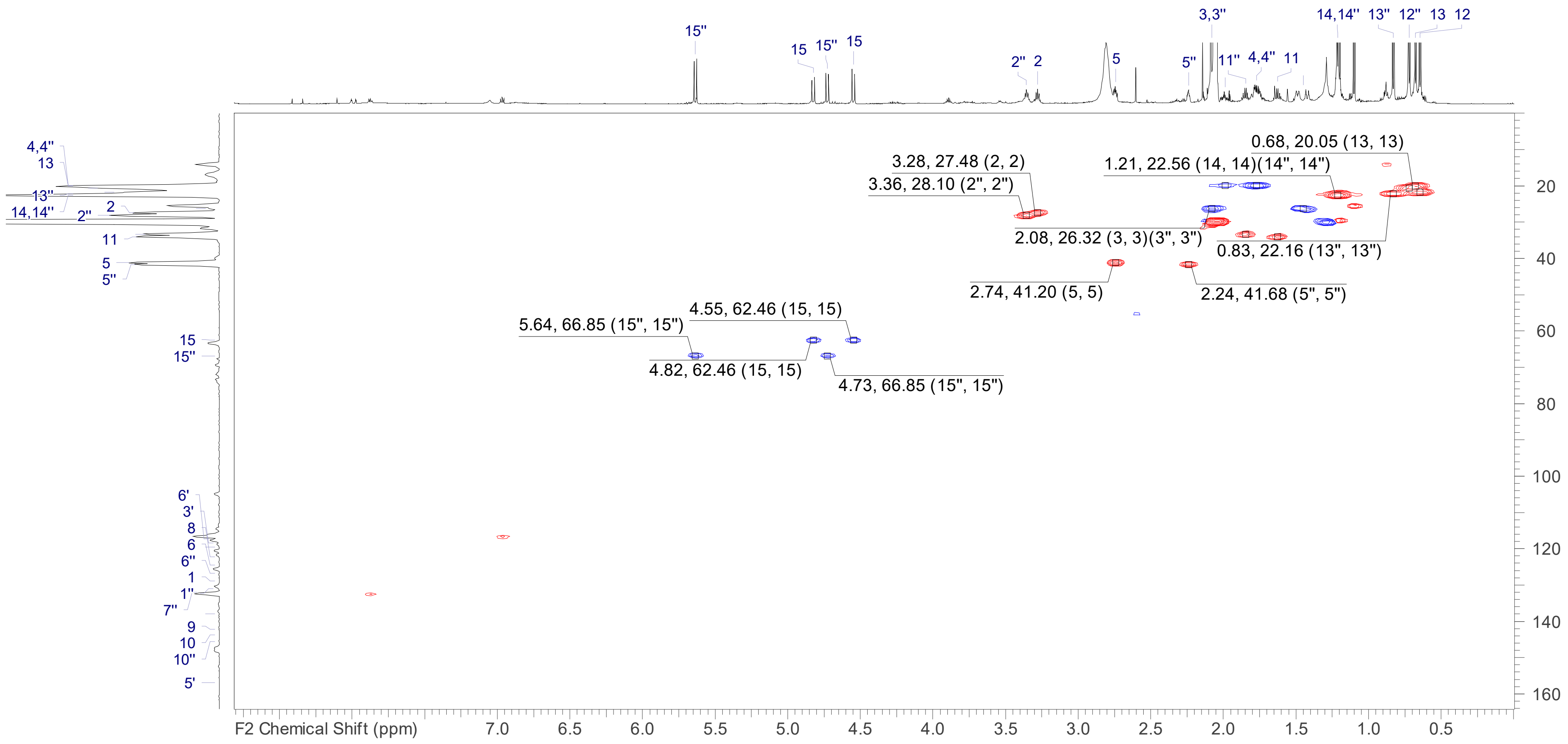

HMBC NMR spectrum (700 MHz, acetone-d_6) of bis-heimiomycin C (**3**).

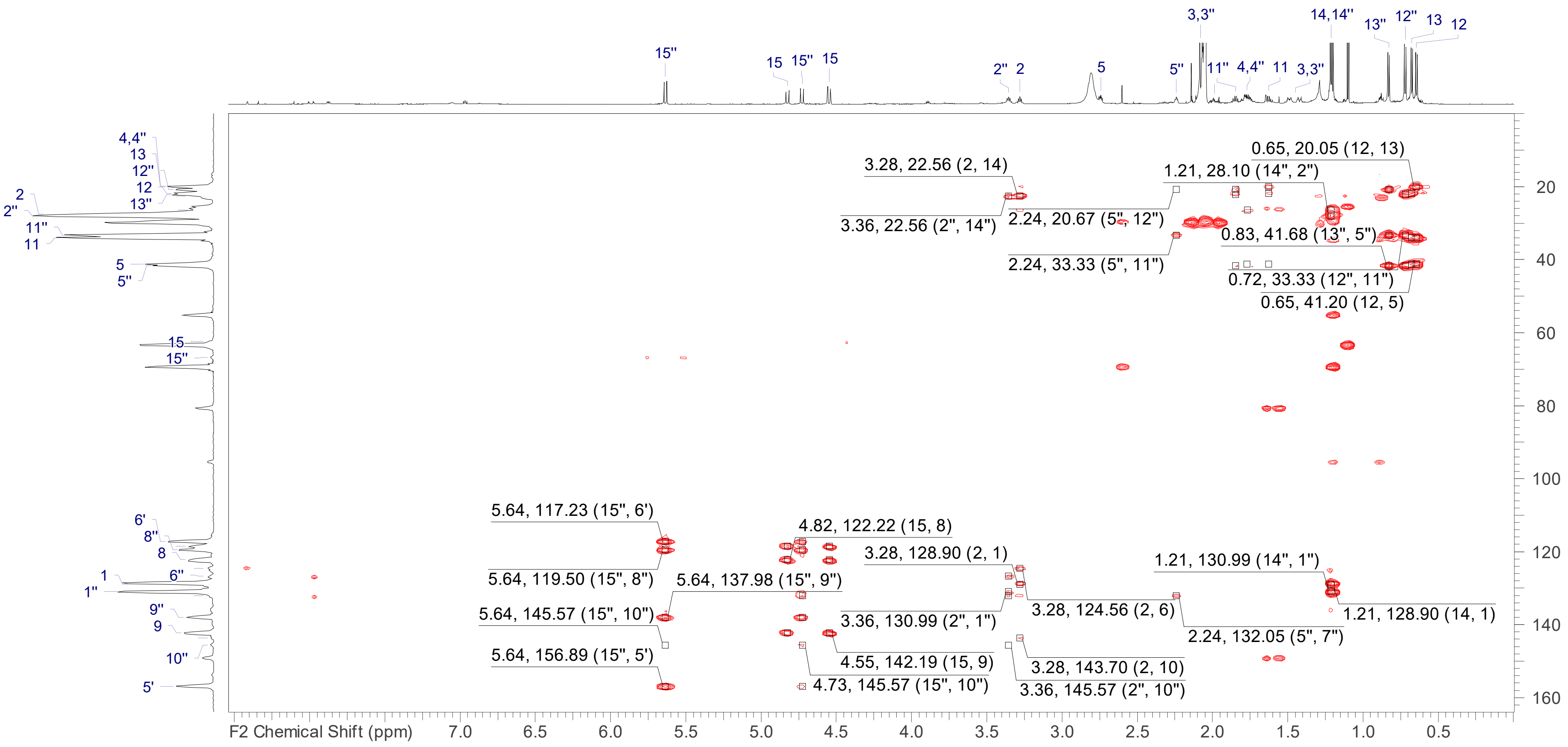

^{1}H NMR spectrum (500 MHz, acetone-d_6) of bis-heimiomycin D (**4**).

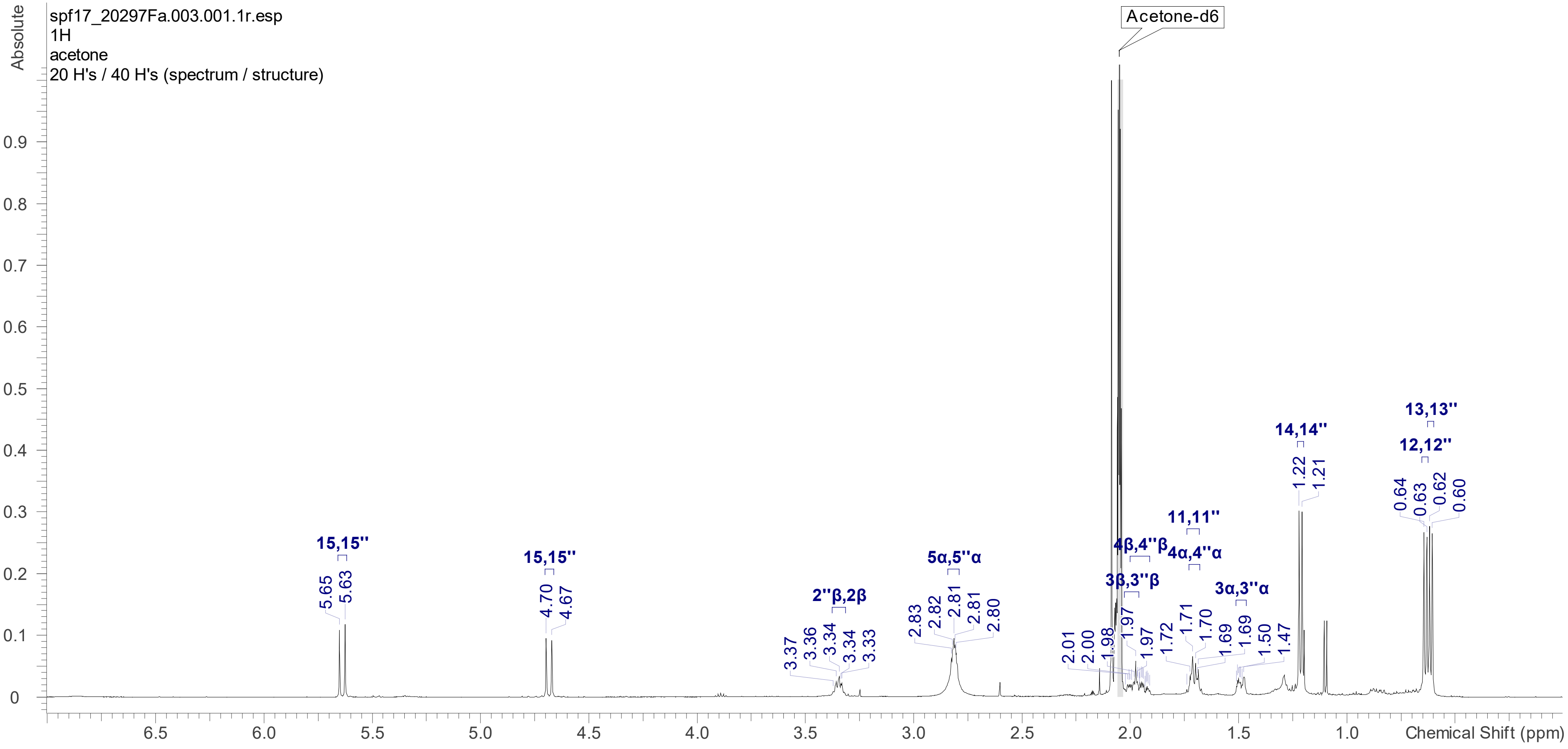

^{13}C NMR spectrum (175 MHz, acetone-d_6) of bis-heimiomycin D (**4**).

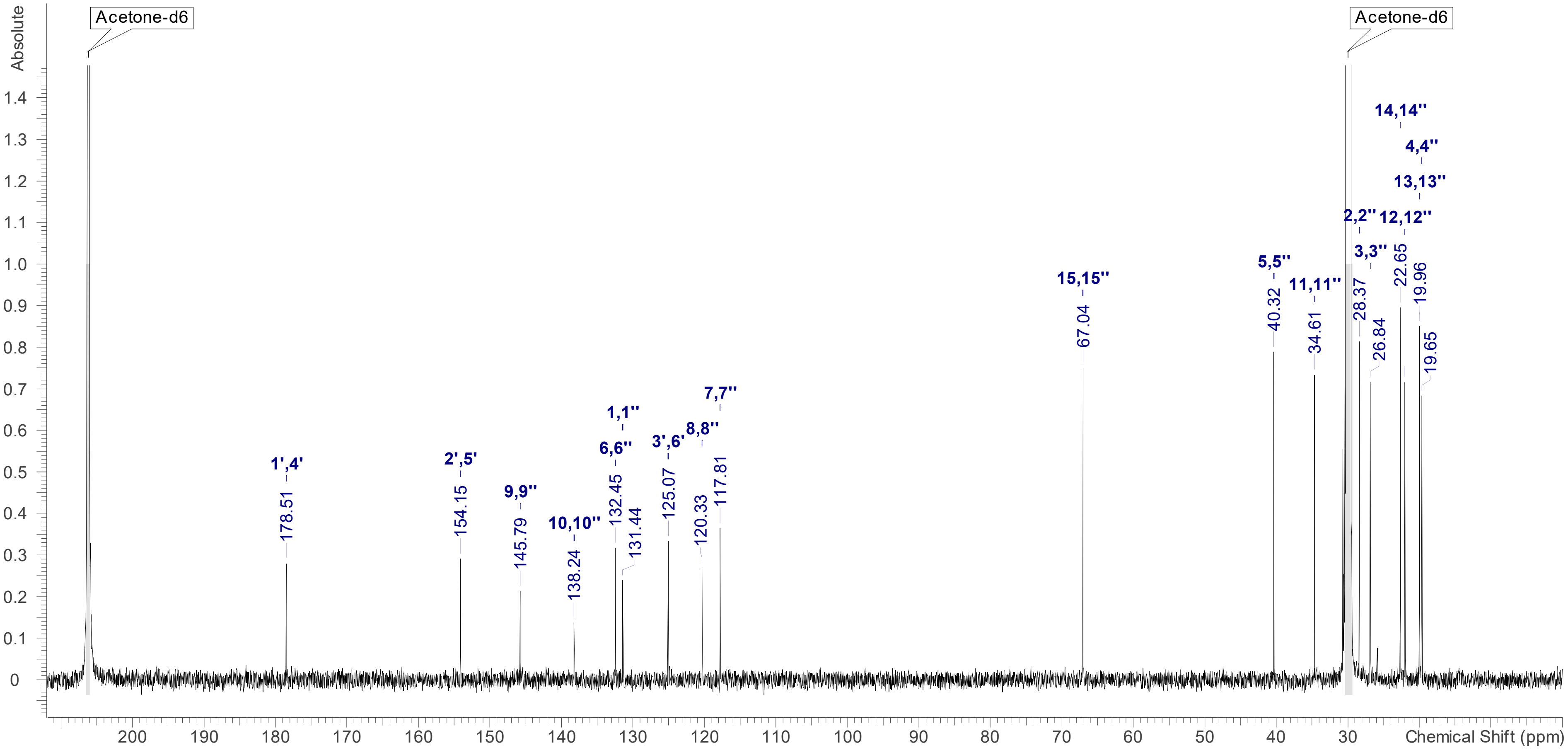

COSY NMR spectrum (500 MHz, acetone-d_6) of bis-heimiomycin D (**4**).

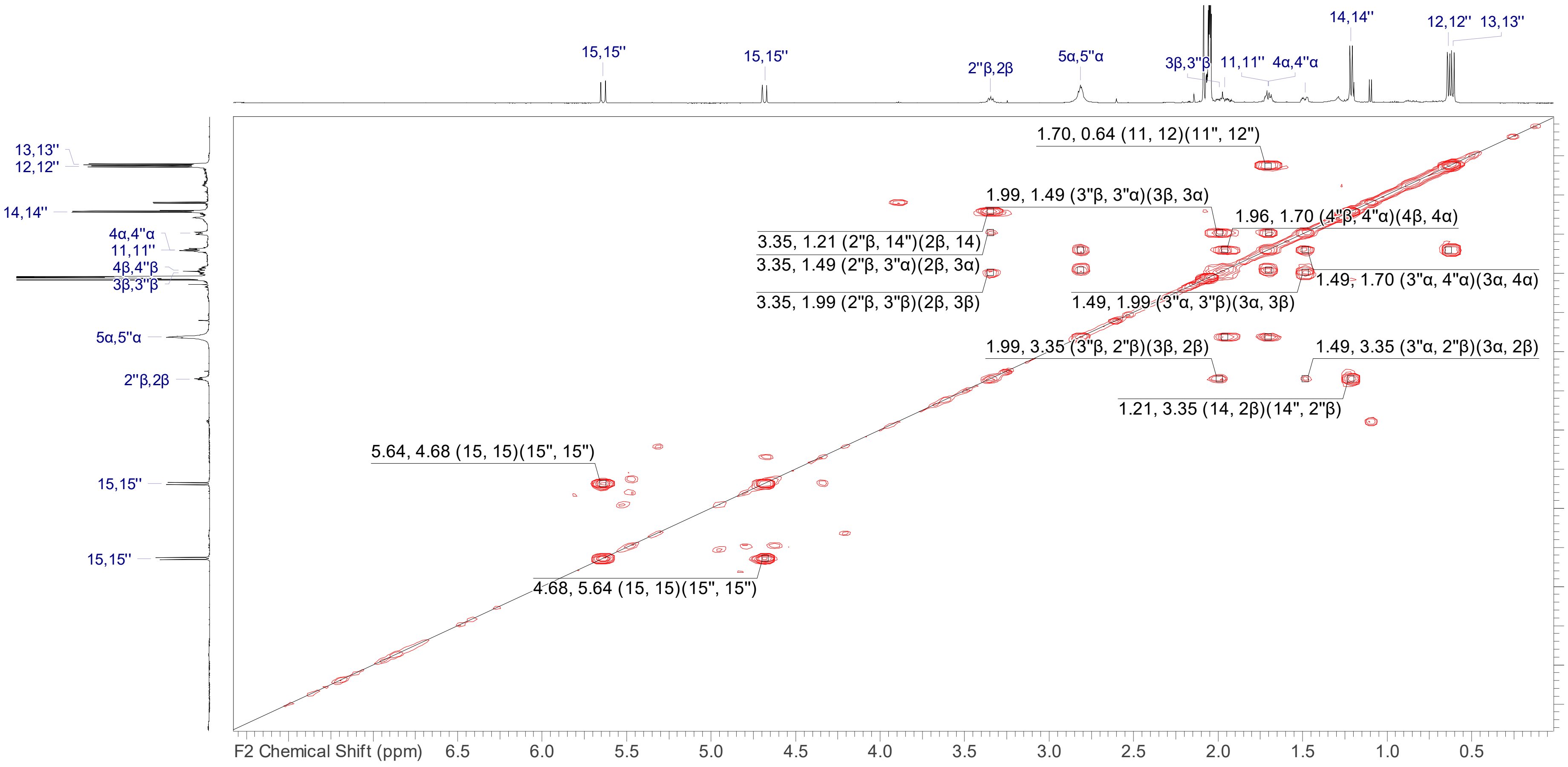

HSQC NMR spectrum (500 MHz, acetone-d_6) of bis-heimiomycin D (**4**).

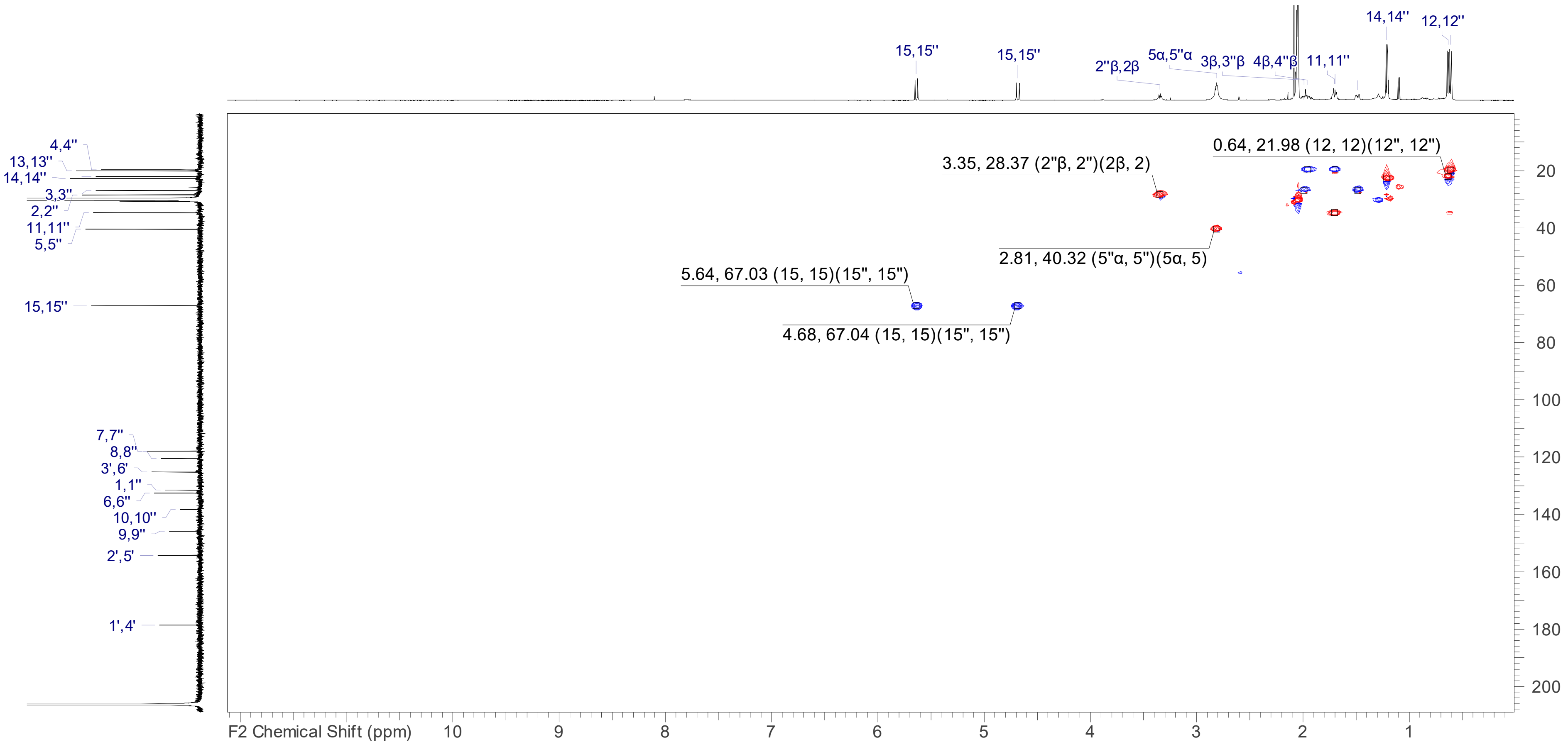

HMBC NMR spectrum (500 MHz, acetone-d_6) of bis-heimiomycin D (**4**).

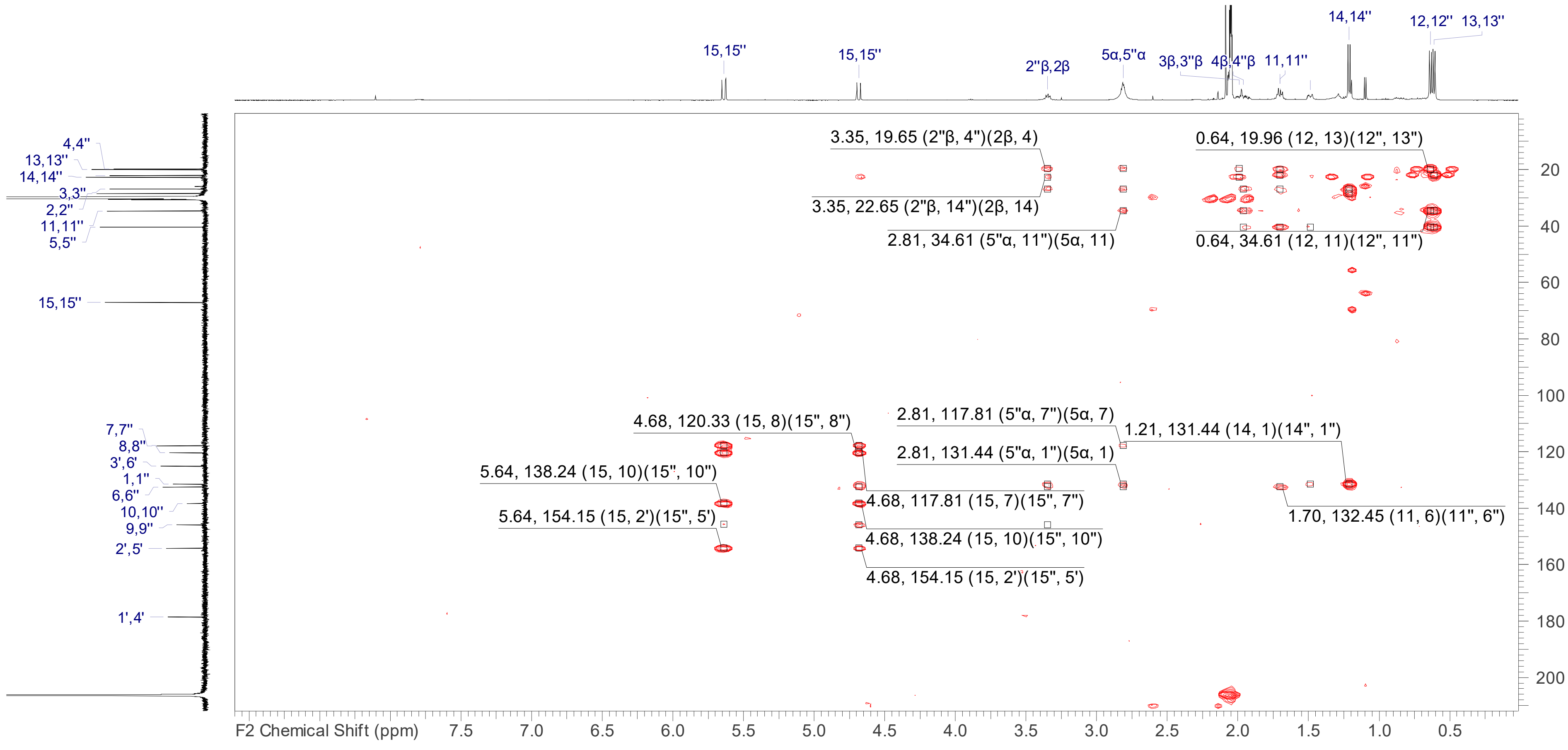

ROESY NMR spectrum (700 MHz, acetone-d_6) of bis-heimiomycin D (**4**).

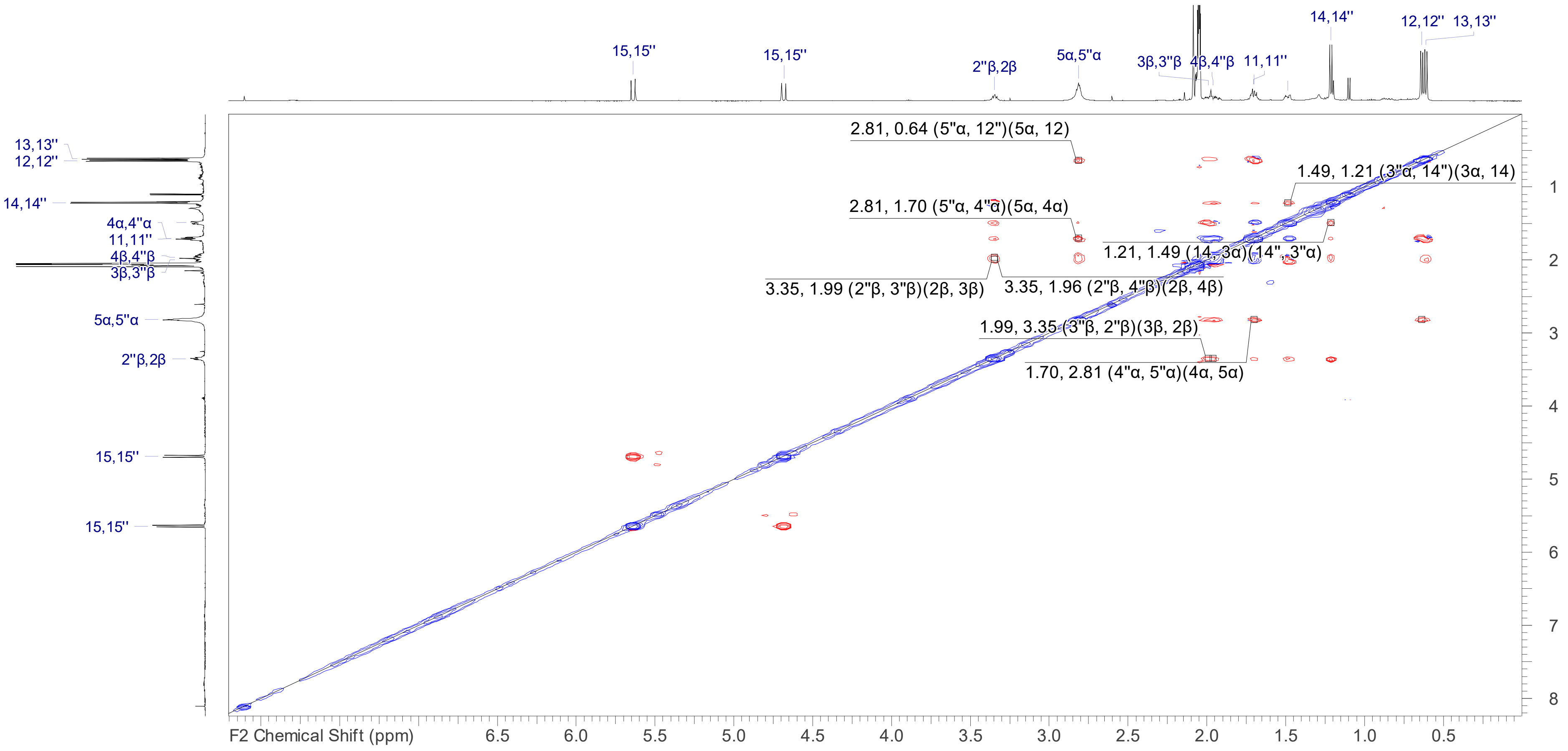

^{1}H NMR spectrum (700 MHz, acetone-d_6) of heimiomycin D (**5**).

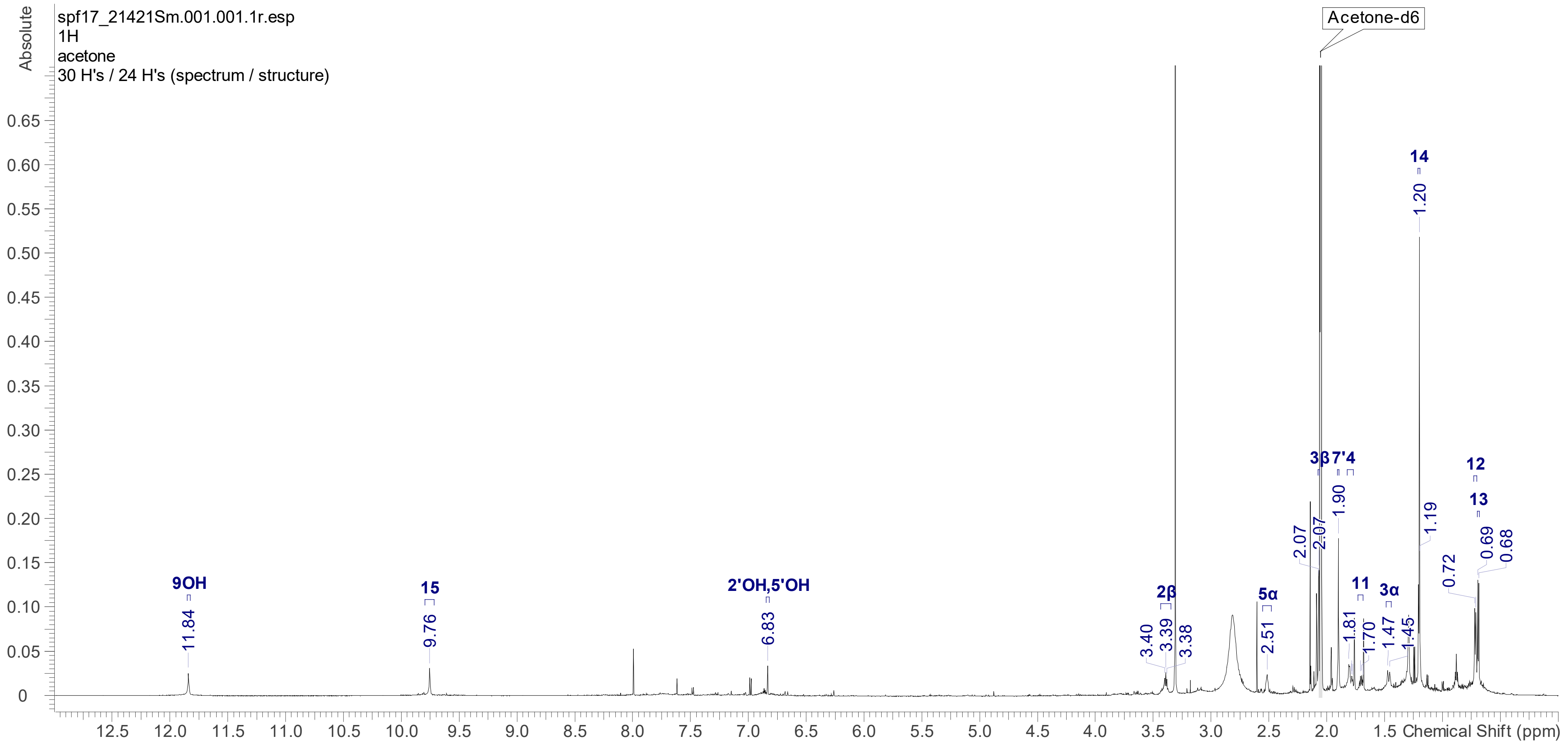

^{13}C NMR spectrum (175 MHz, acetone-d_6) of heimiomycin D (**5**).

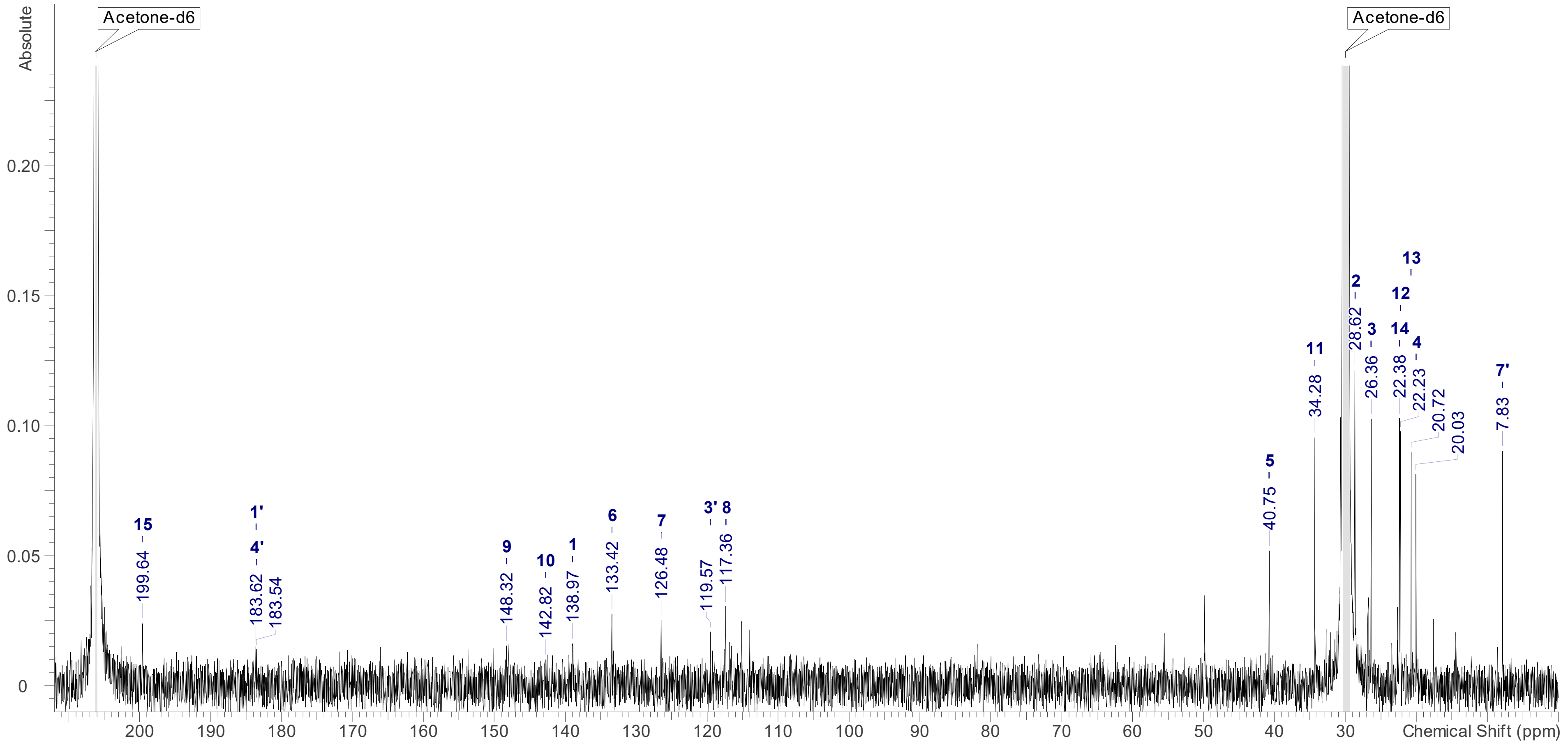

COSY NMR spectrum (700 MHz, acetone-d_6) of heimiomycin D (**5**).

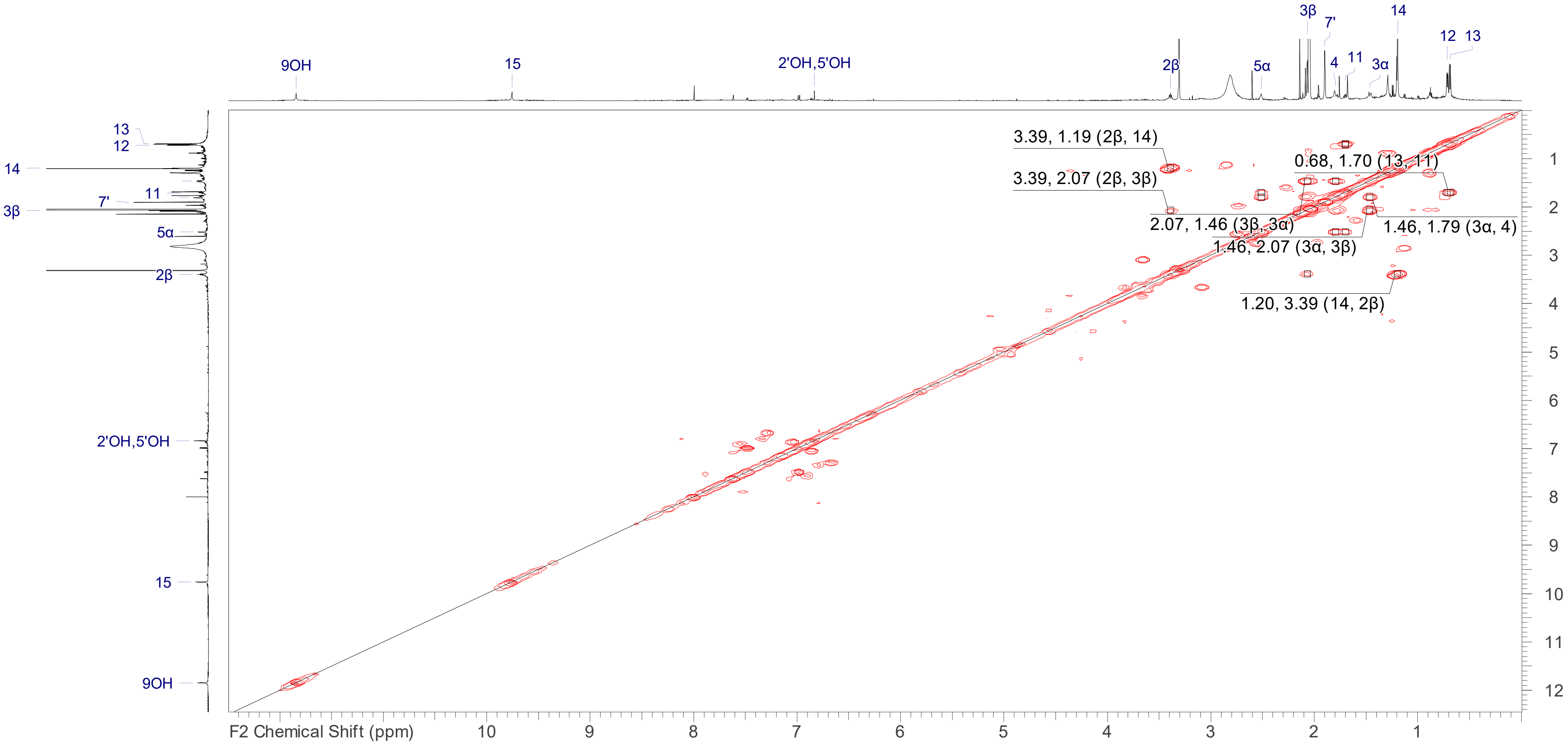

HSQC NMR spectrum (700 MHz, acetone-d_6) of heimiomycin D (**5**).

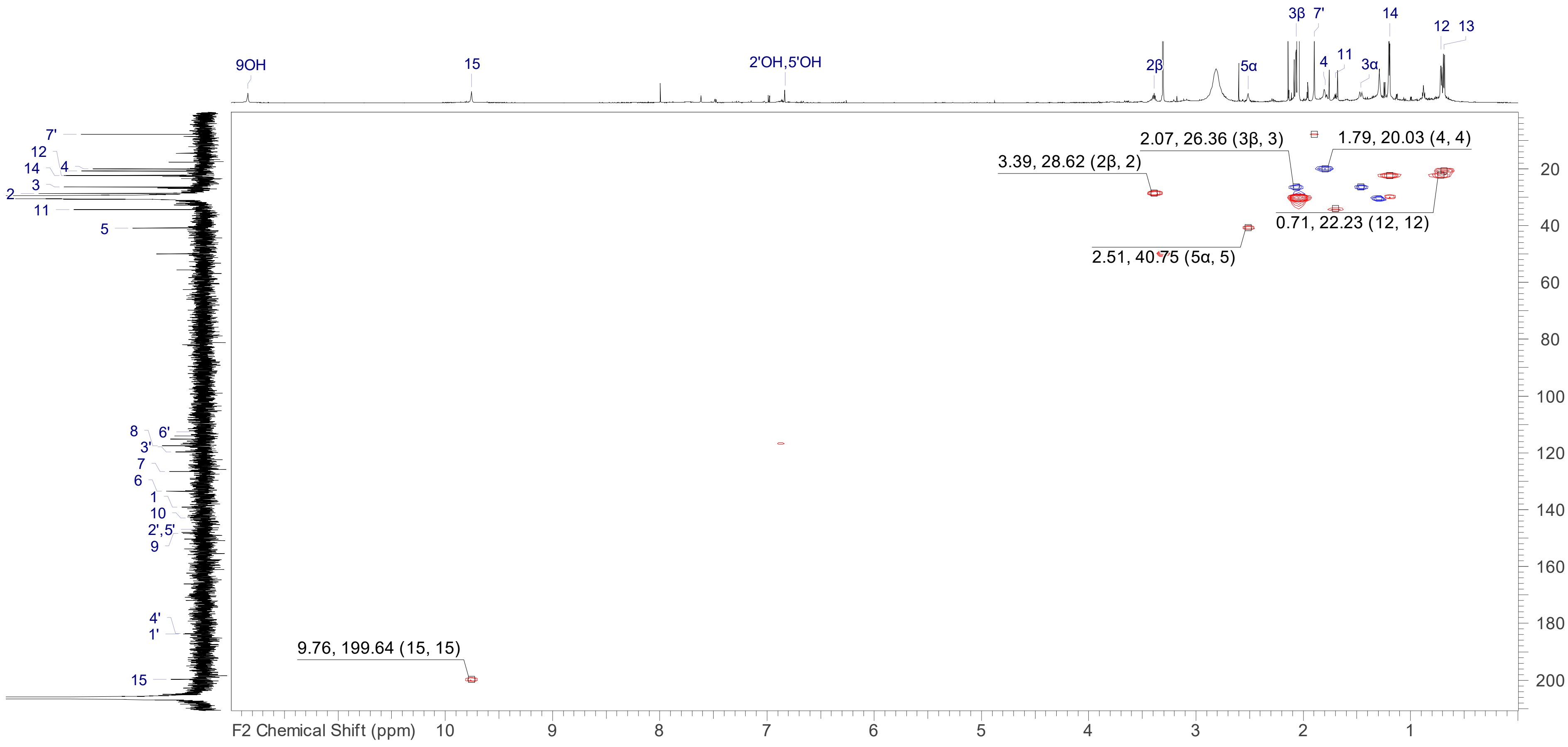

HMBC NMR spectrum (700 MHz, acetone-d_6) of heimiomycin D (**5**).

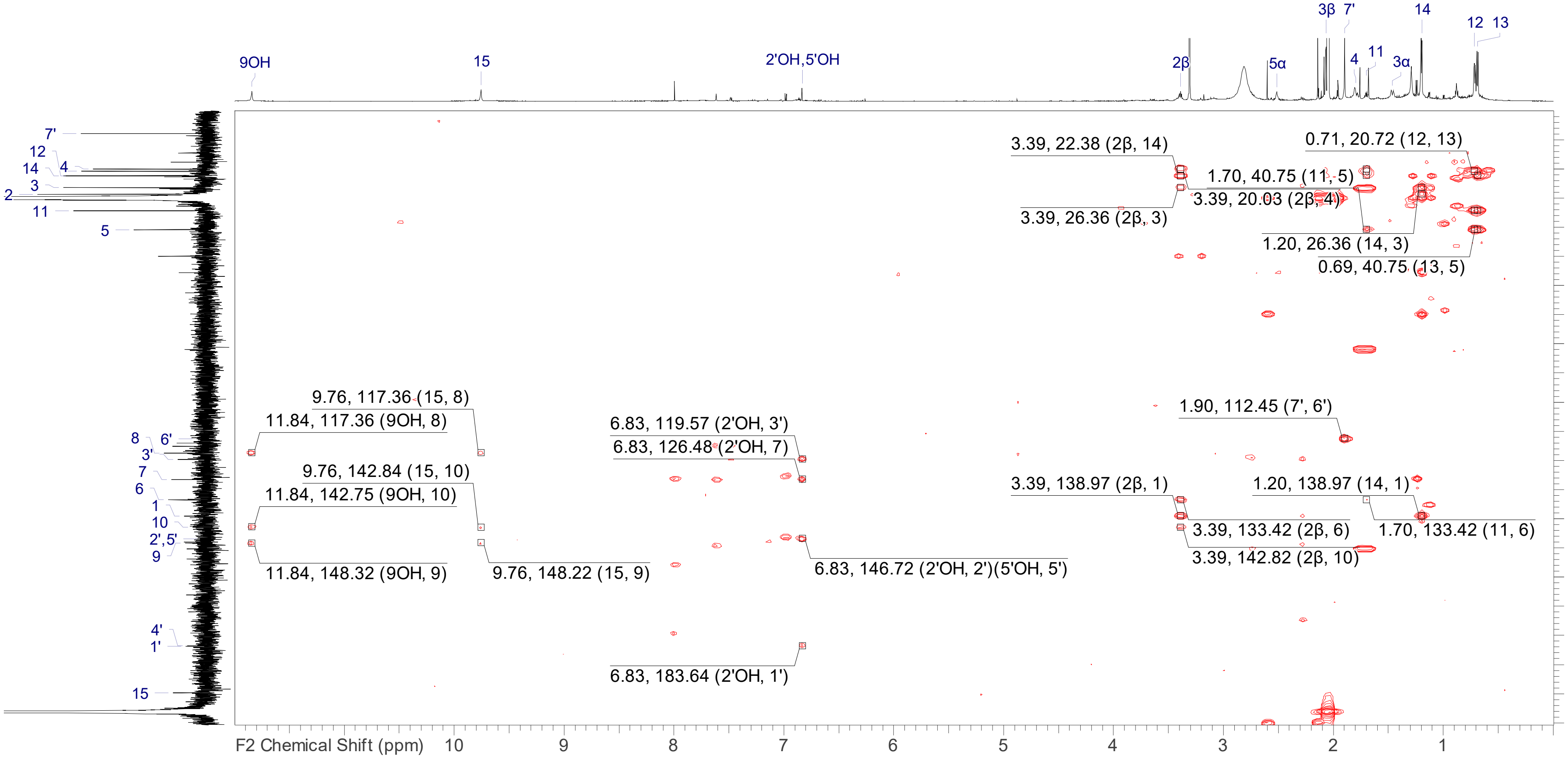

ROESY NMR spectrum (700 MHz, acetone-d_6) of heimiomycin D (**5**).

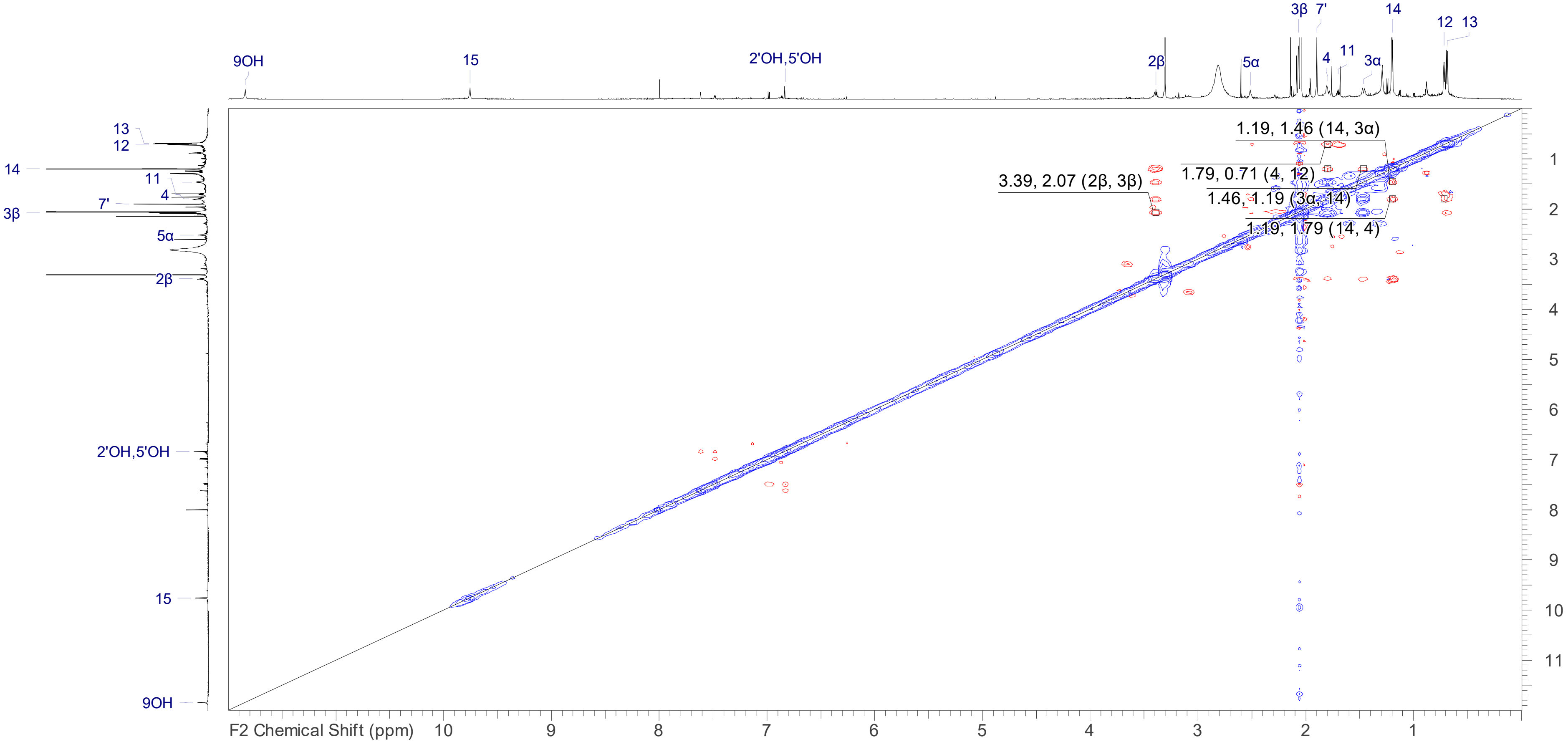

^{1}H NMR spectrum (500 MHz, acetone-d_6) of heimiomycin E (**6**).

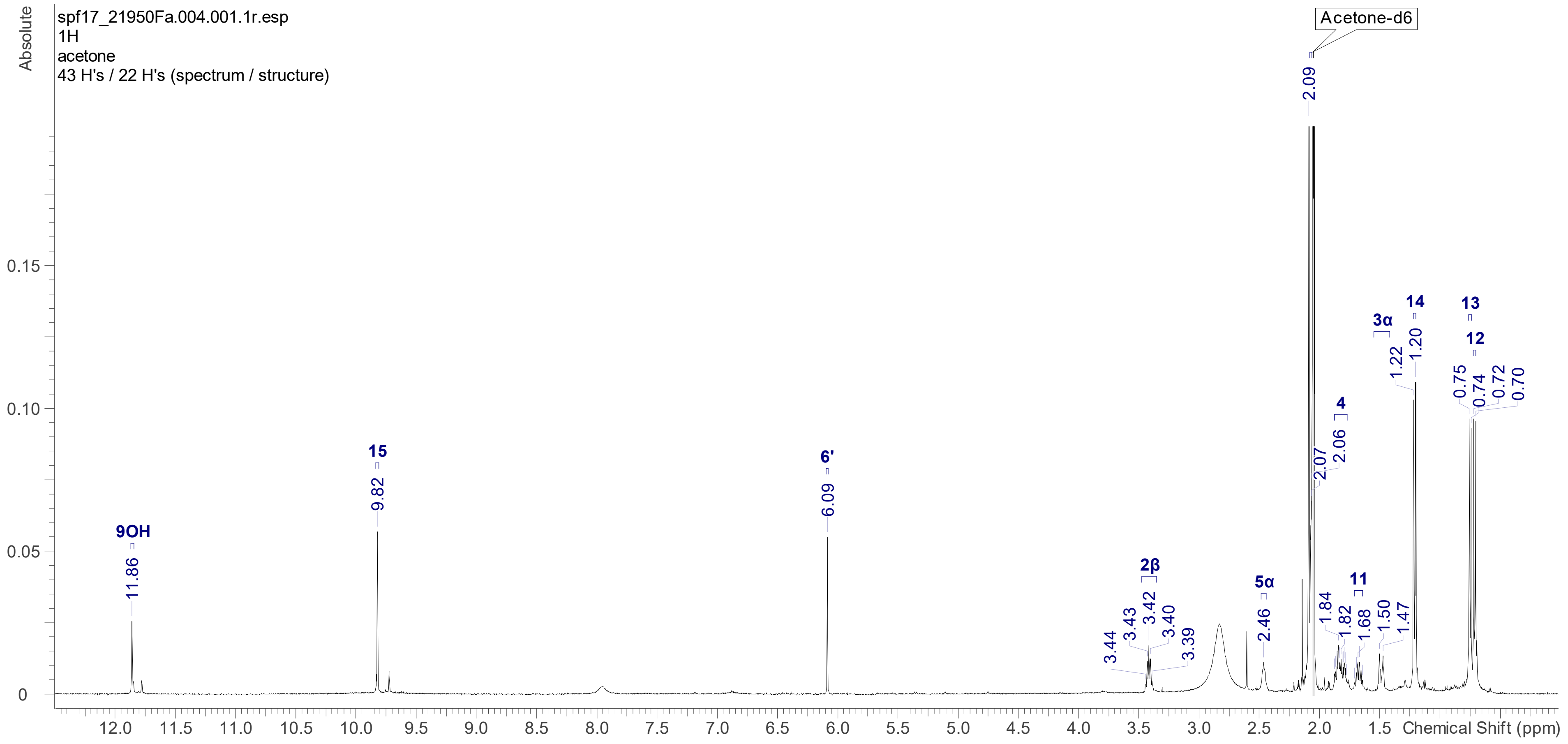

^{13}C NMR spectrum (125 MHz, acetone-d_6) of heimiomycin E (**6**).

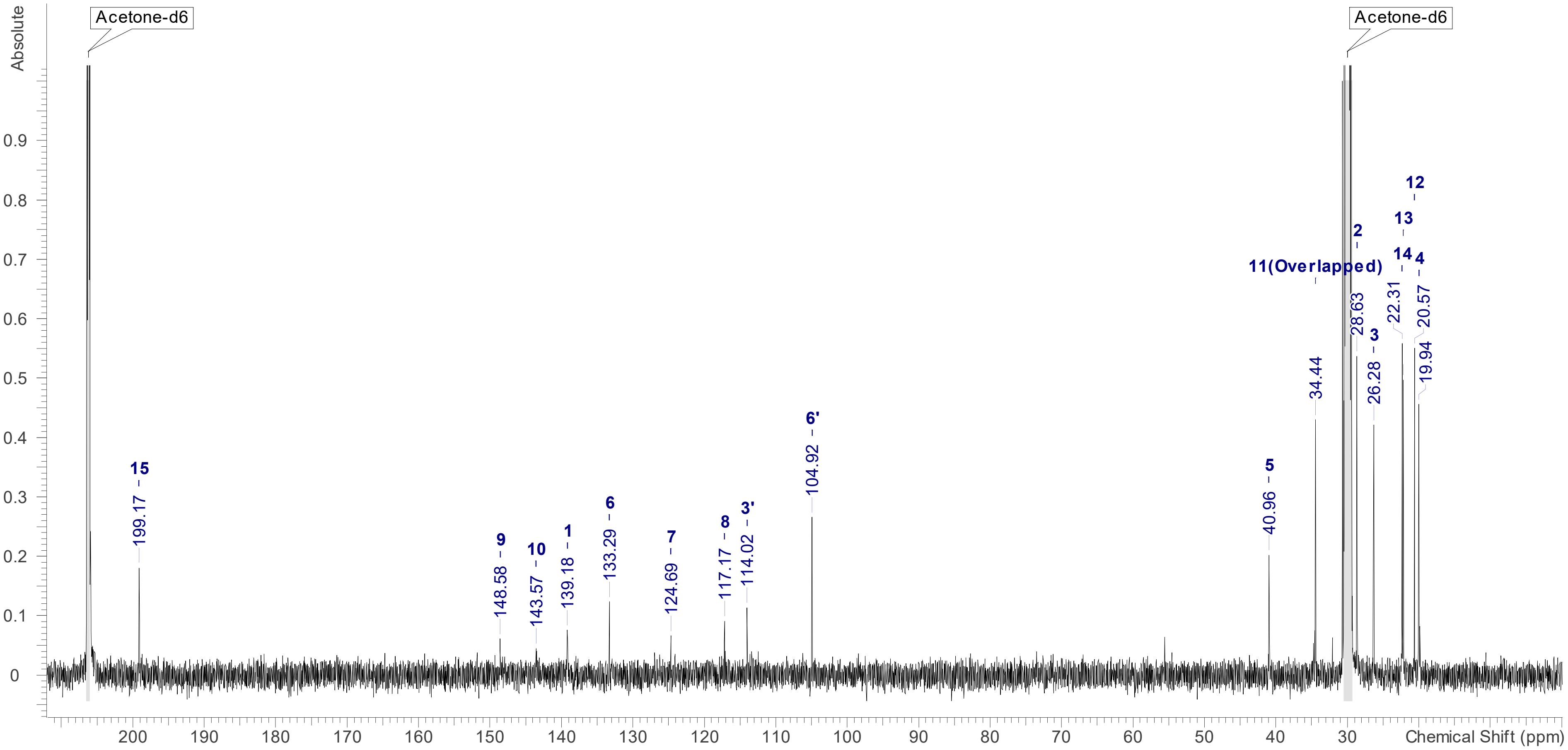

COSY NMR spectrum (500 MHz, acetone-d_6) of heimiomycin E (**6**).

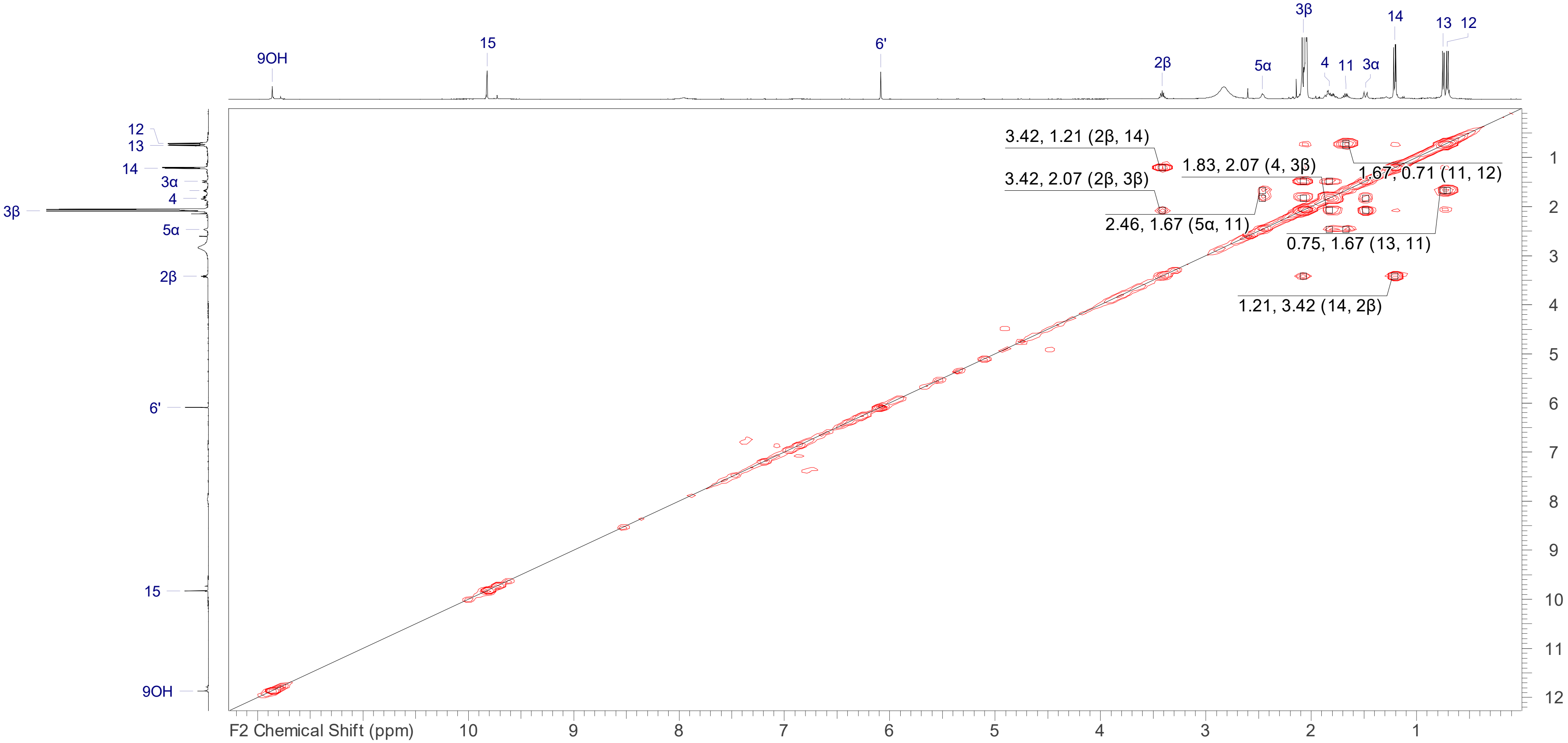

HSQC NMR spectrum (500 MHz, acetone-d_6) of heimiomycin E (**6**).

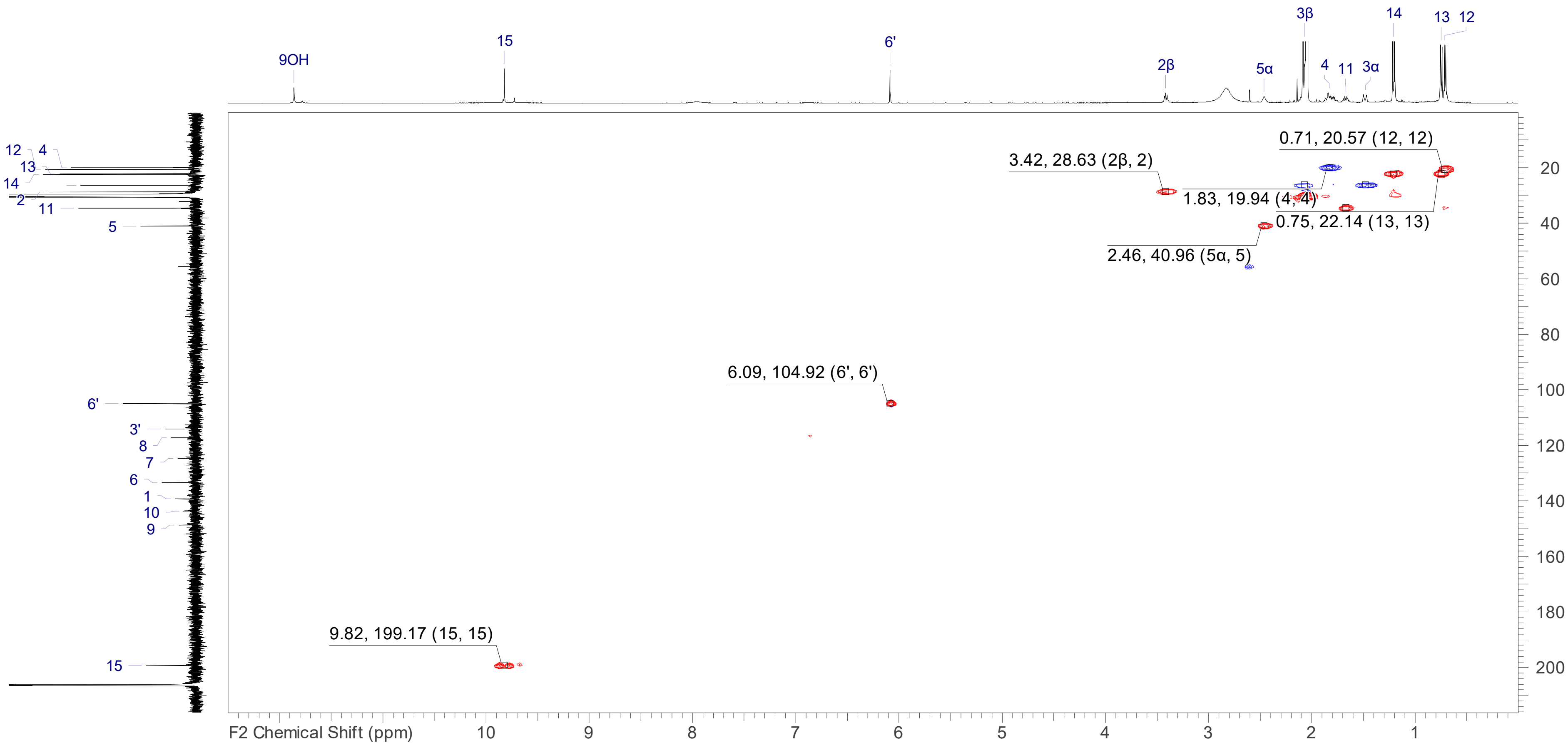

HMBC NMR spectrum (500 MHz, acetone-d_6) of heimiomycin E (**6**).

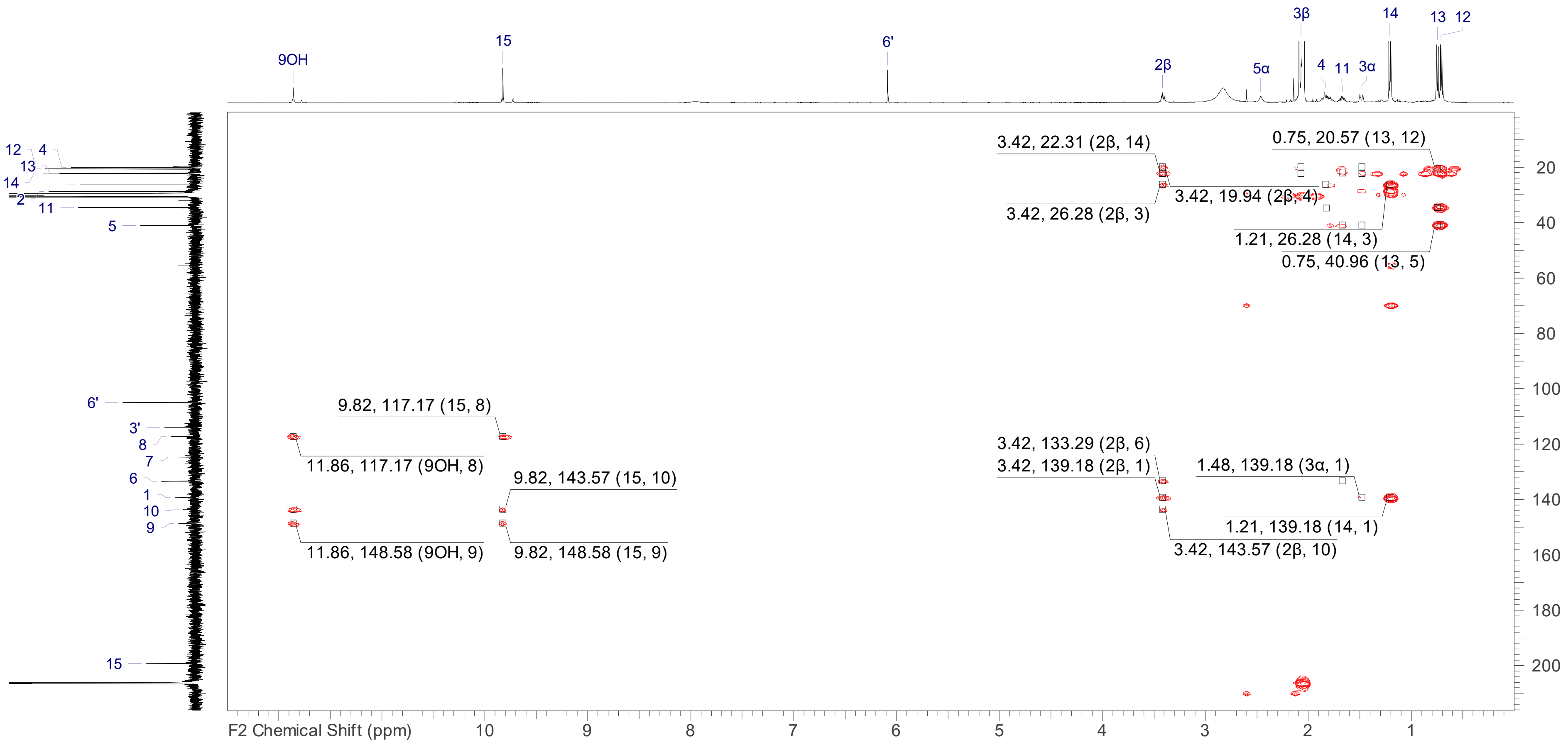

ROESY NMR spectrum (500 MHz, acetone-d_6) of heimiomycin E (**6**).

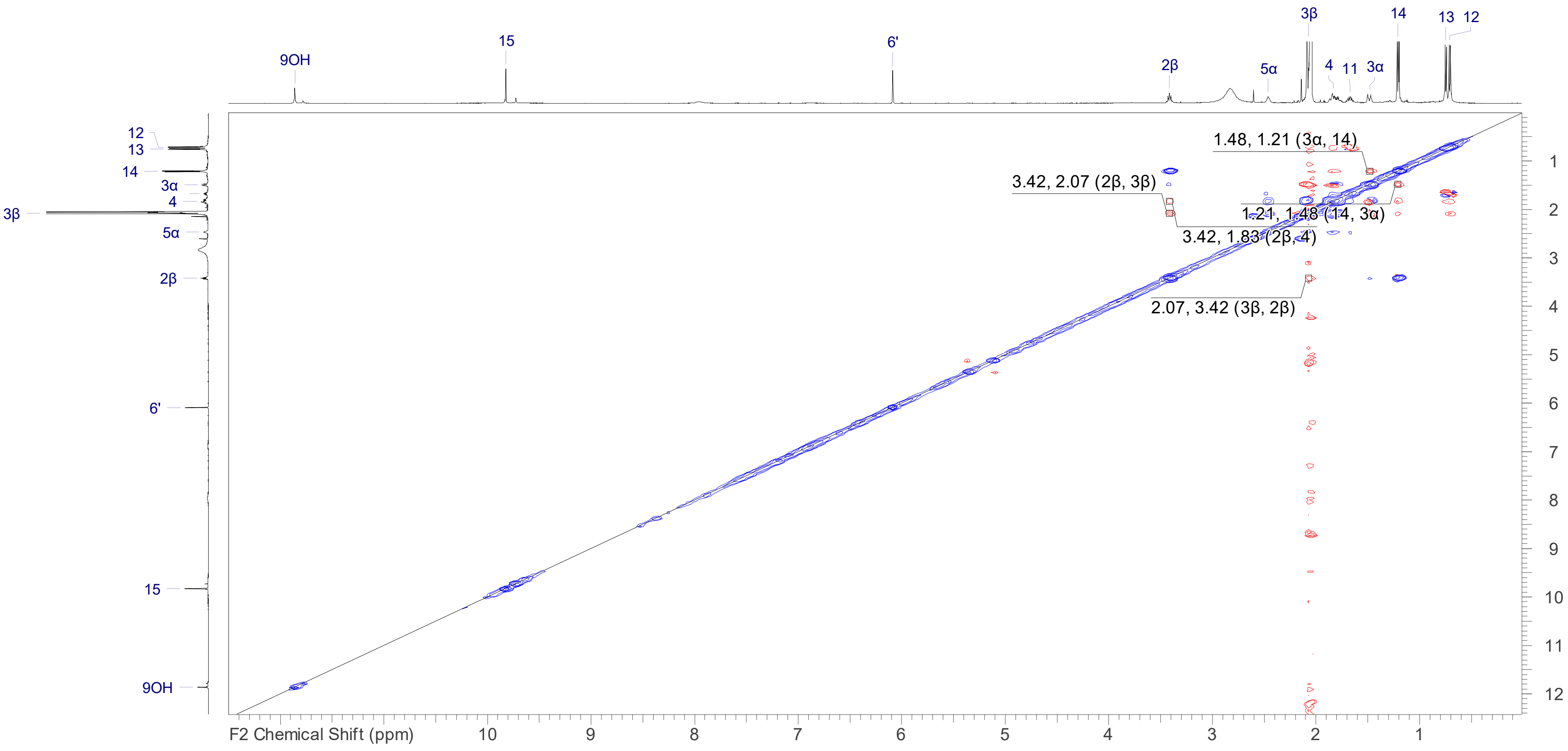

^{1}H NMR spectrum (500 MHz, acetone-d_6) of heimiocalamene A (**7**).

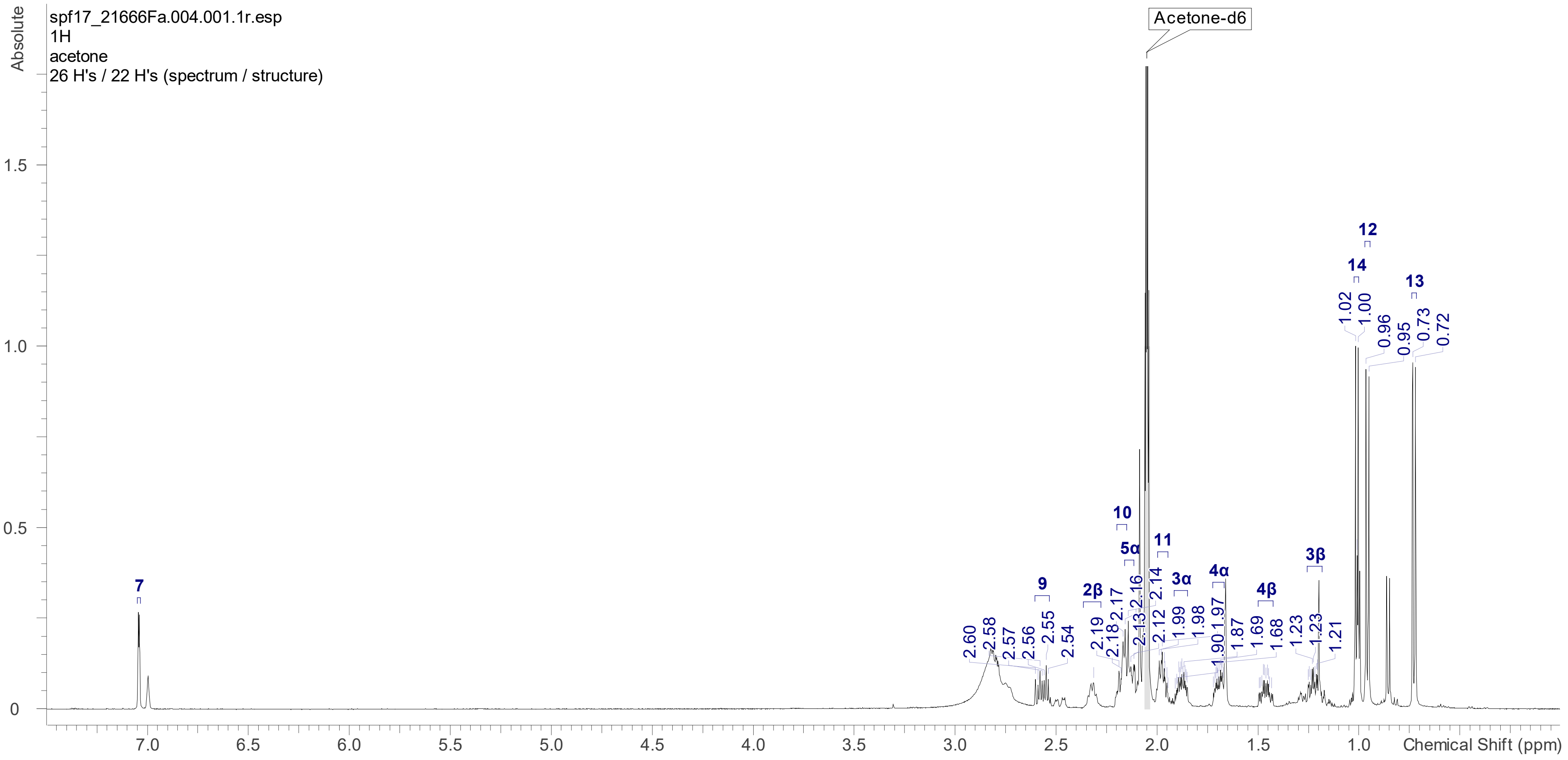

^{13}C NMR spectrum (125 MHz, acetone-*d*$_6$) of heimiocalamene A (**7**).

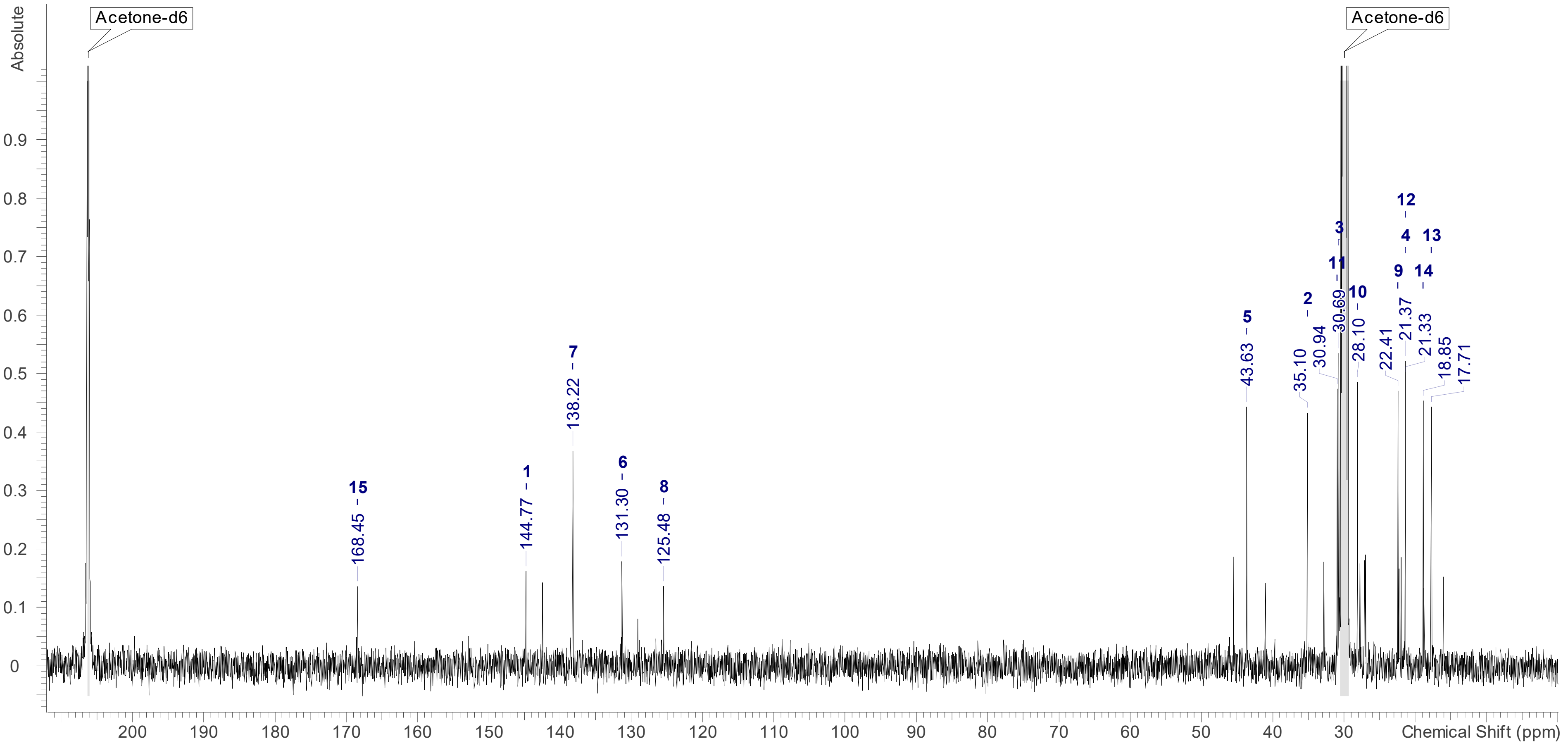

COSY NMR spectrum (500 MHz, acetone-d_6) of heimiocalamene A (**7**).

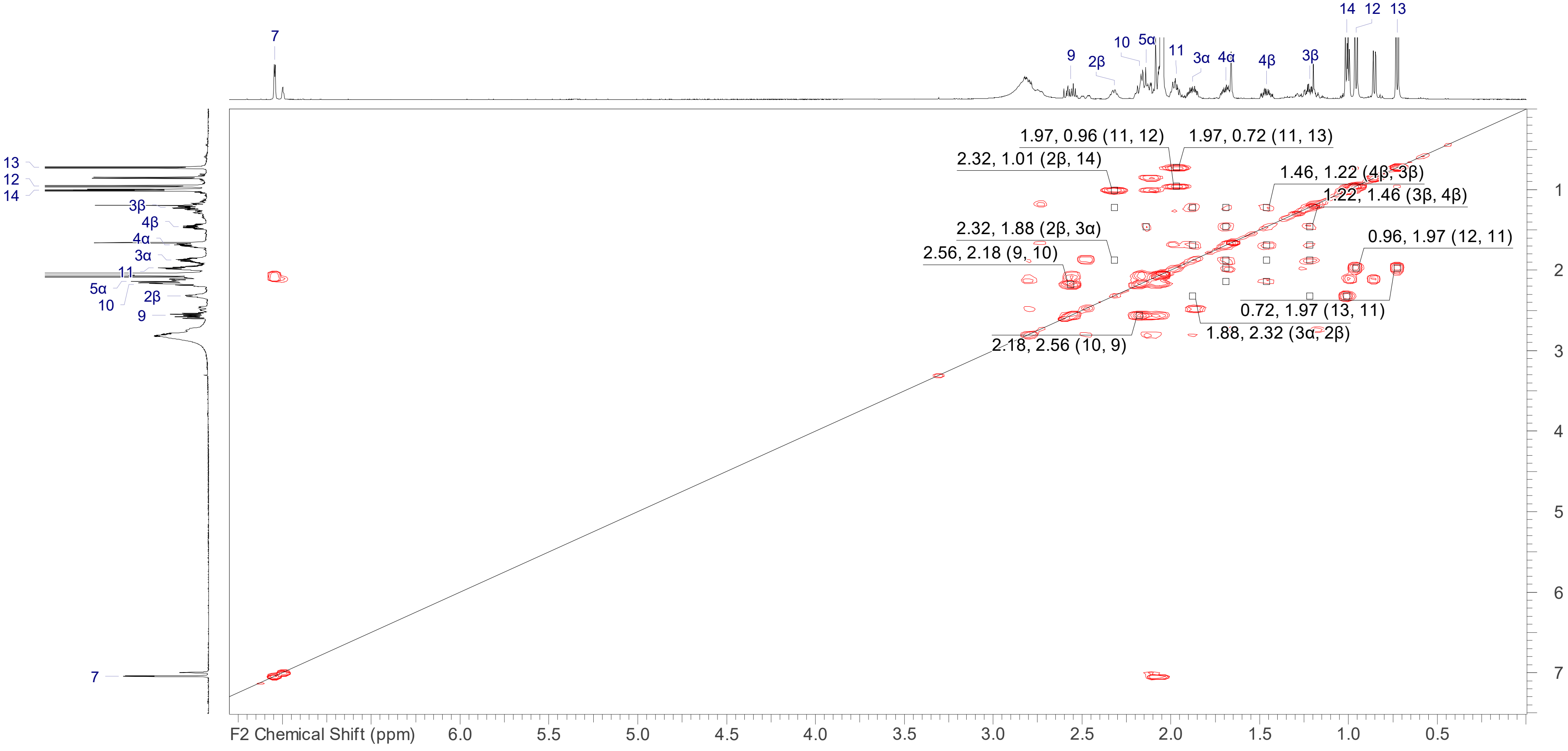

HSQC NMR spectrum (500 MHz, acetone-d_6) of heimiocalamene A (**7**).

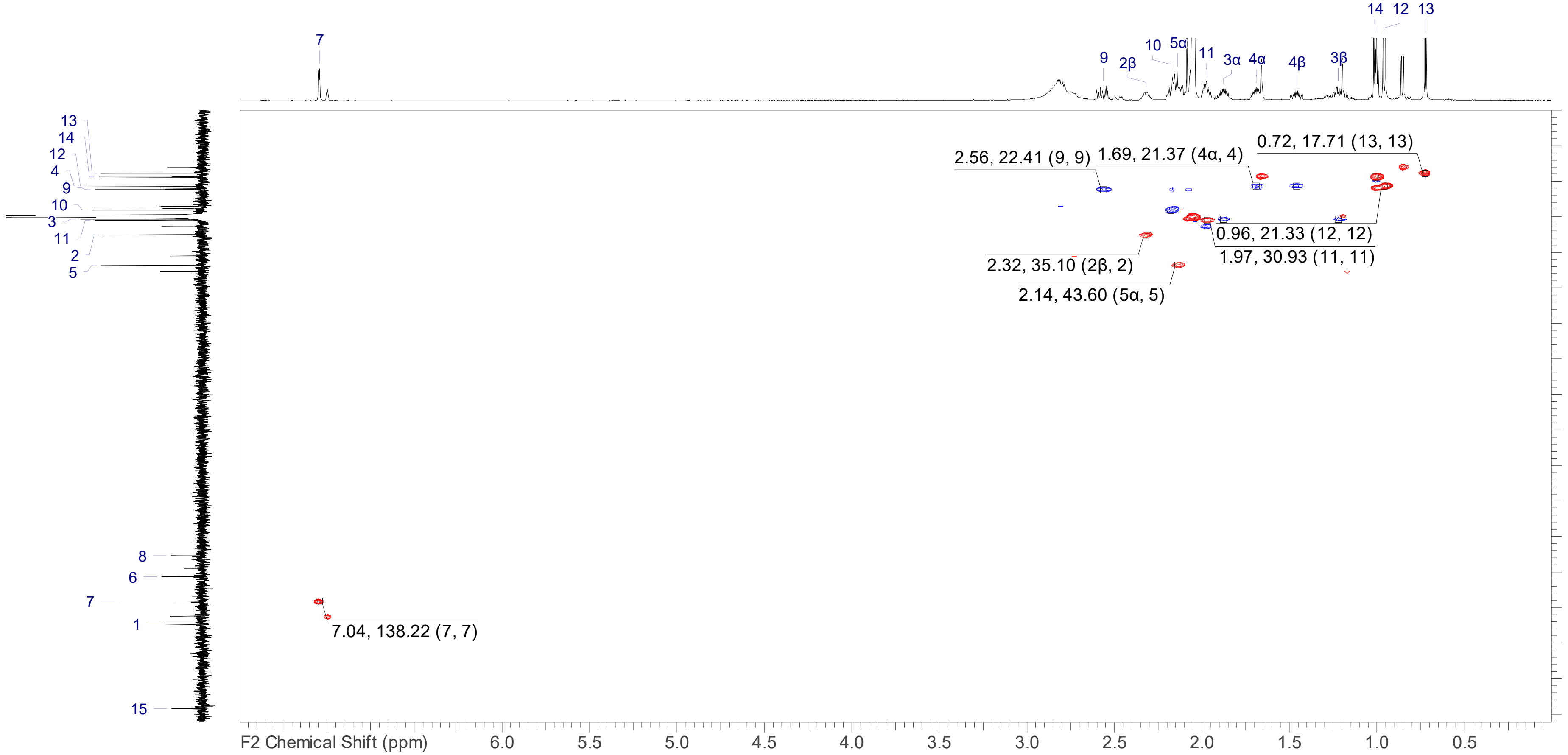

HMBC NMR spectrum (500 MHz, acetone-d_6) of heimiocalamene A (**7**).

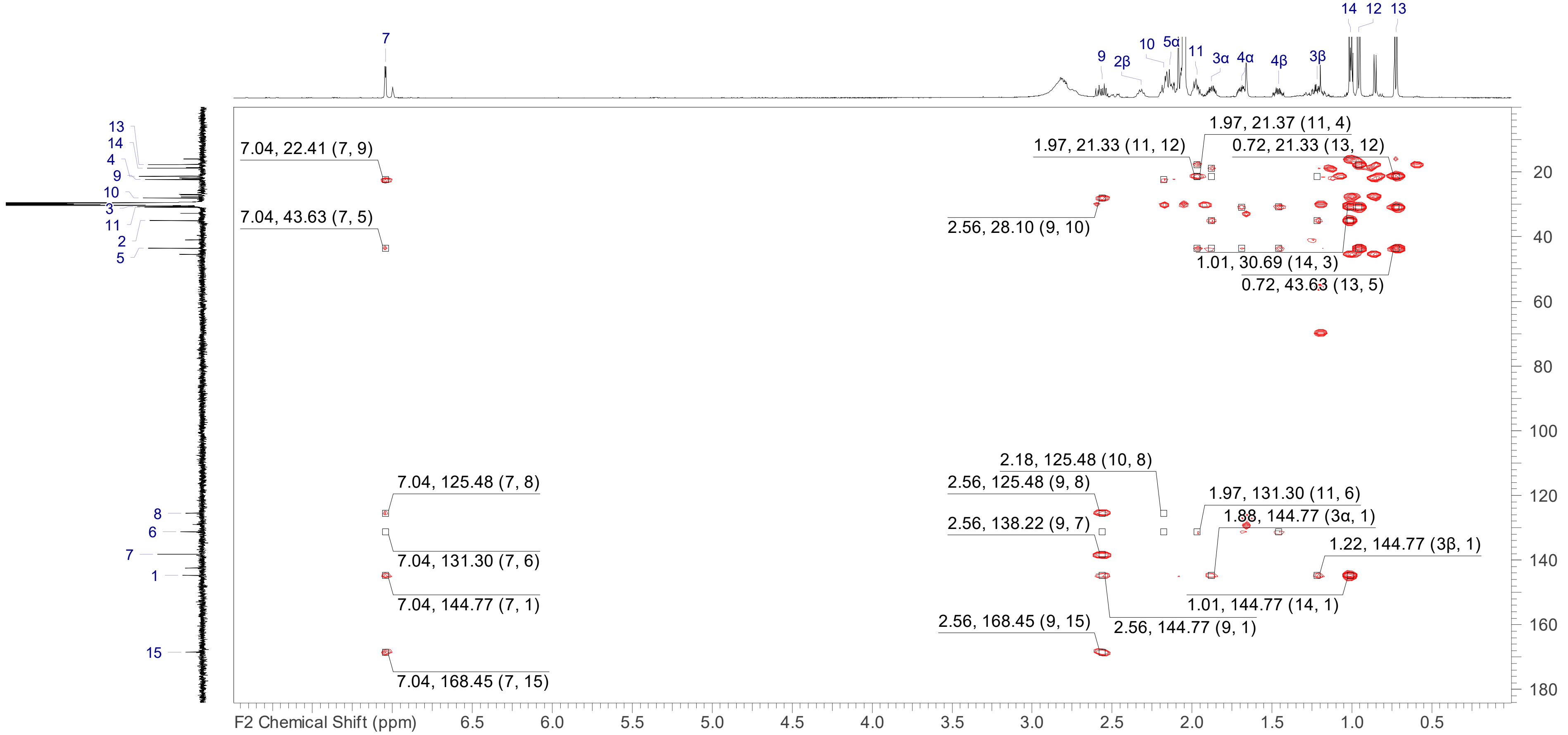

ROESY NMR spectrum (500 MHz, acetone-d_6) of heimiocalamene A (**7**).

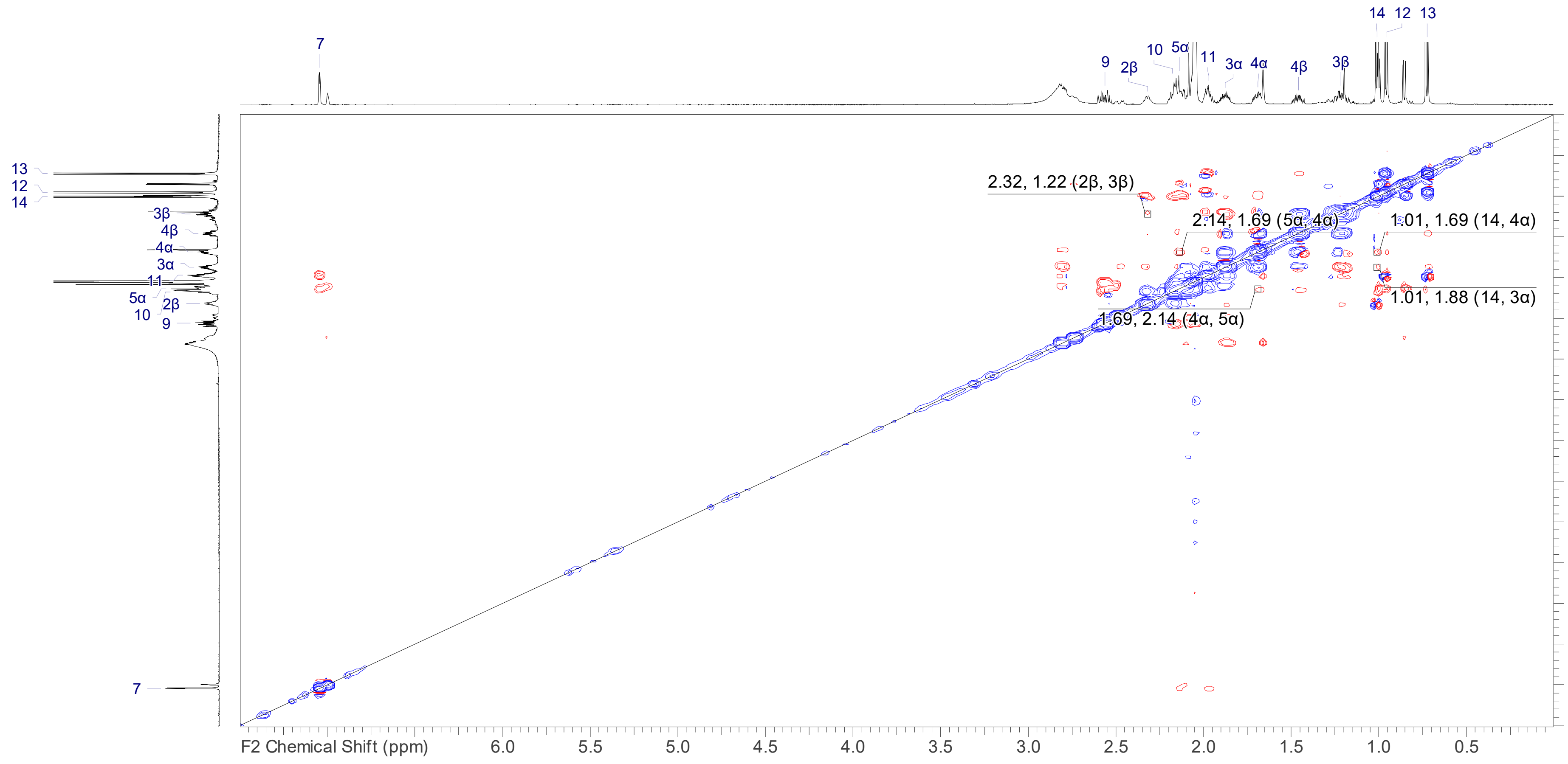

^{1}H NMR spectrum (500 MHz, methanol-d_4) of heimiocalamene B (**8**).

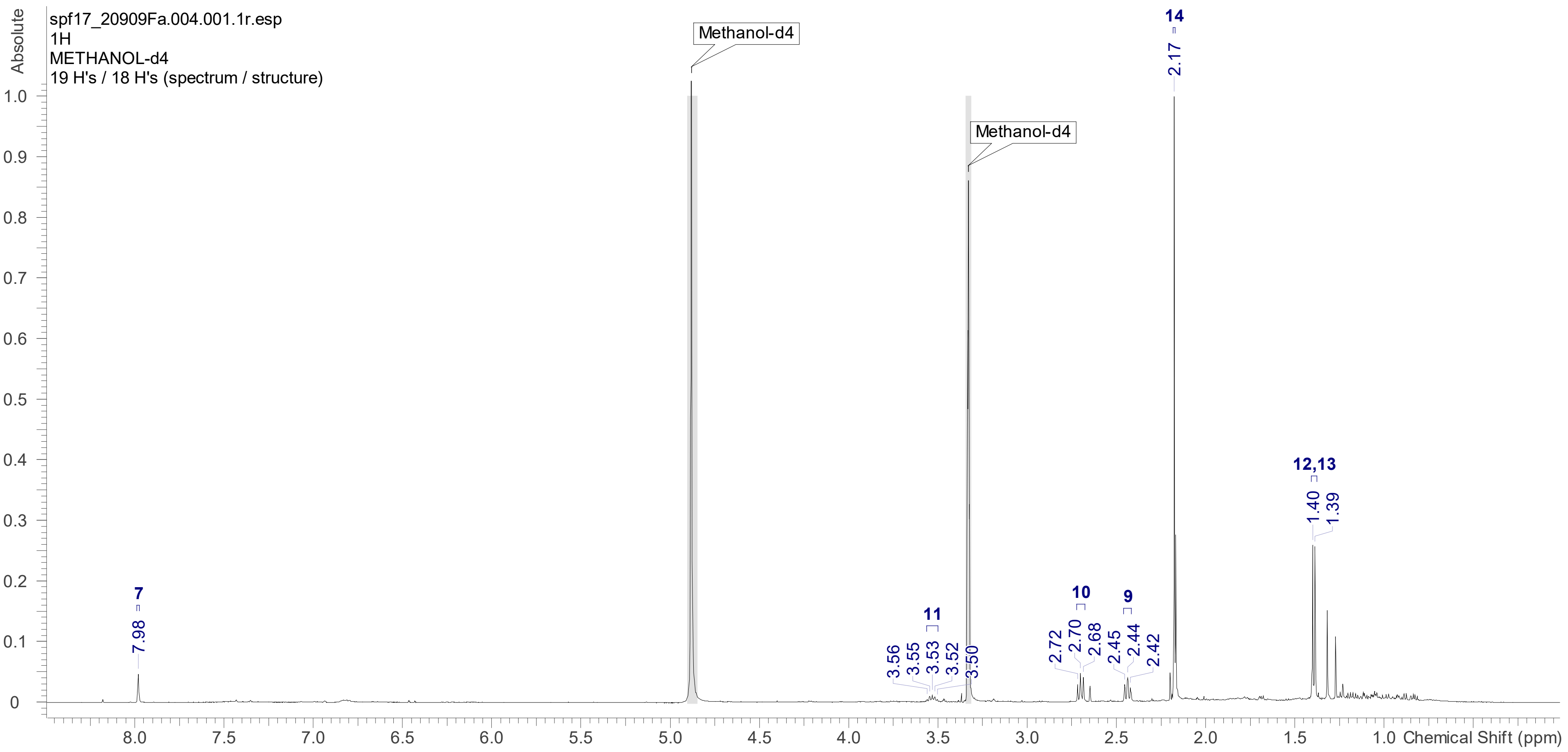

^{13}C NMR spectrum (125 MHz, methanol-d_4) of heimiocalamene B (**8**).

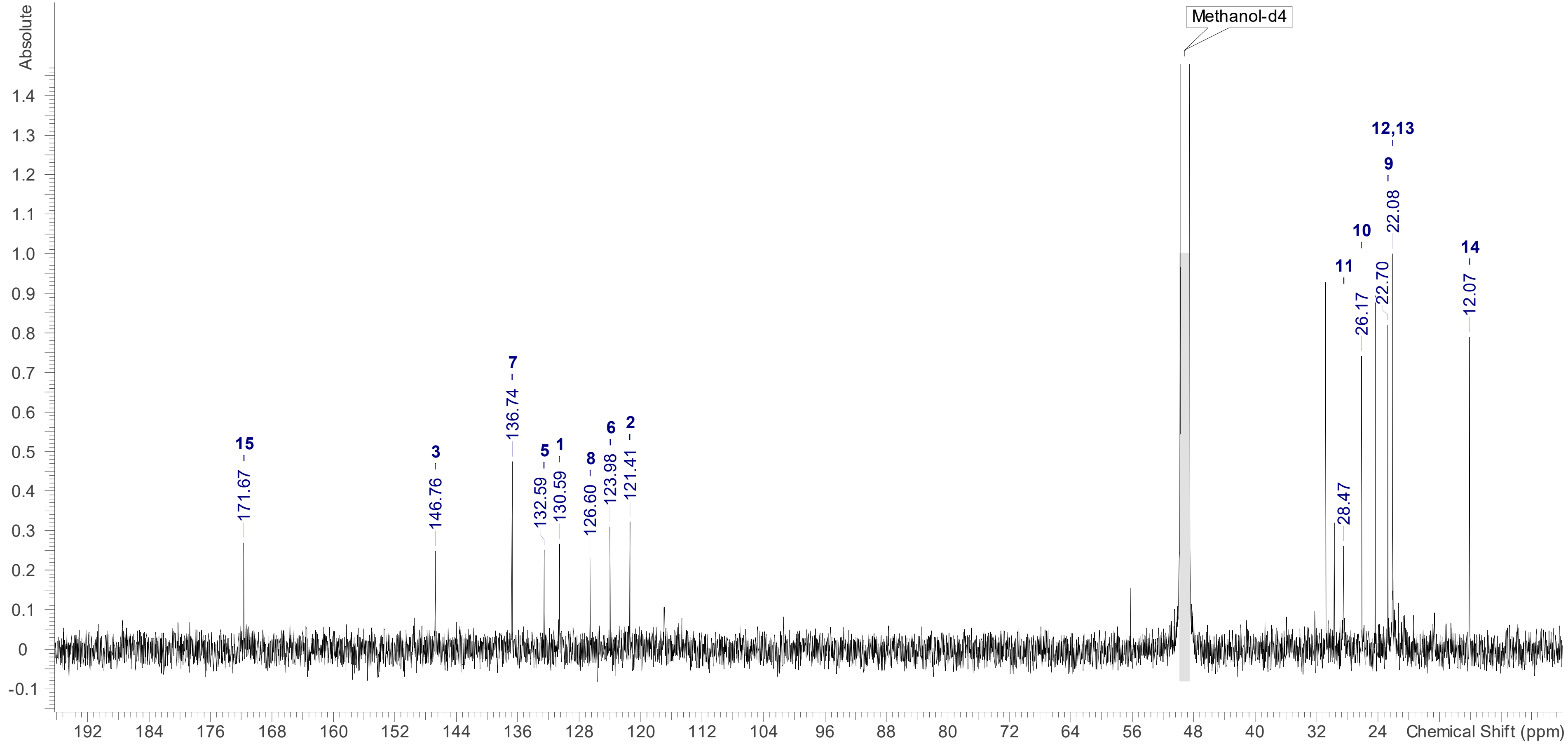

COSY NMR spectrum (500 MHz, methanol-d_4) of heimiocalamene B (**8**).

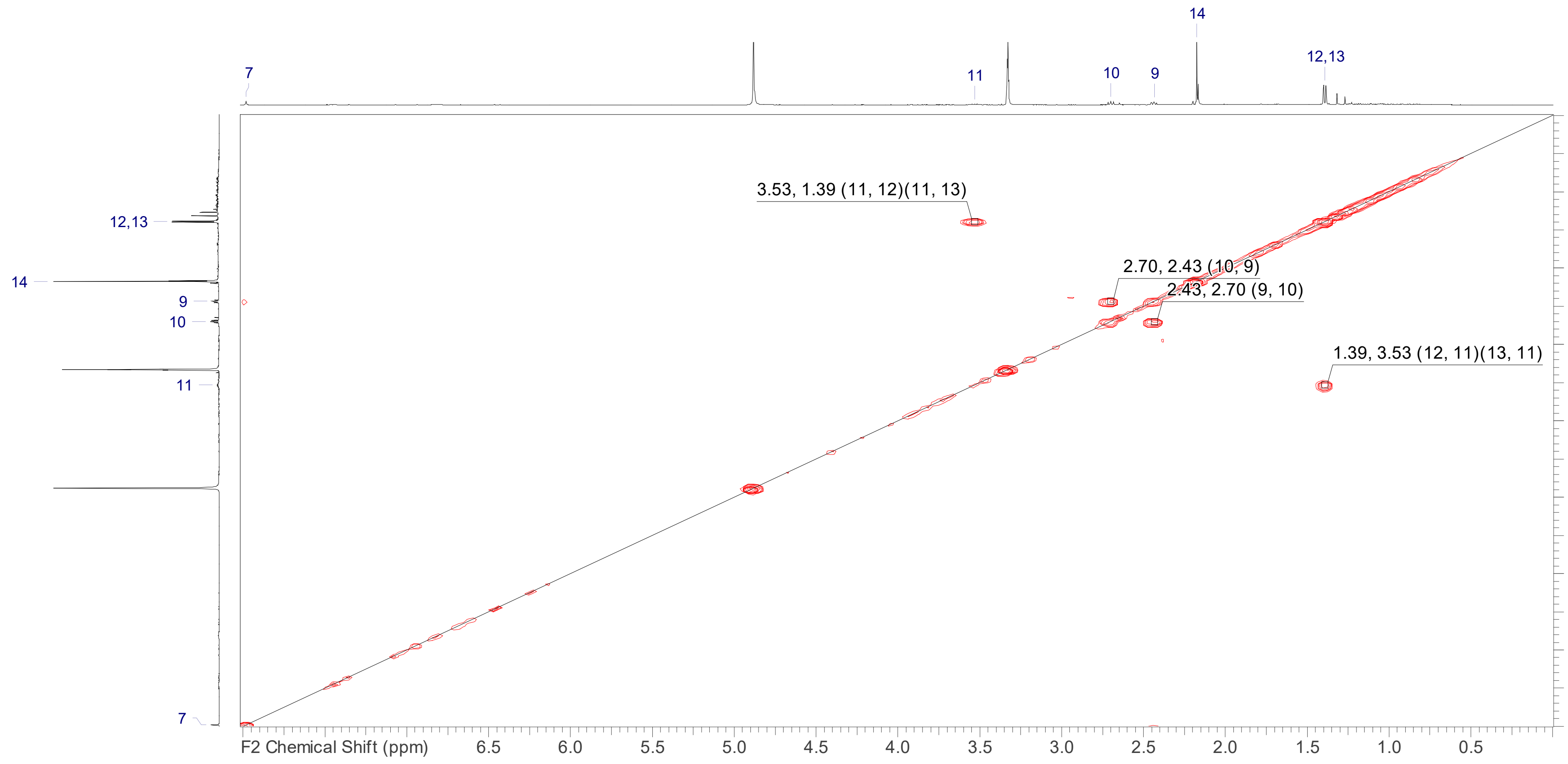

HSQC NMR spectrum (500 MHz, methanol-d_4) of heimiocalamene B (**8**).

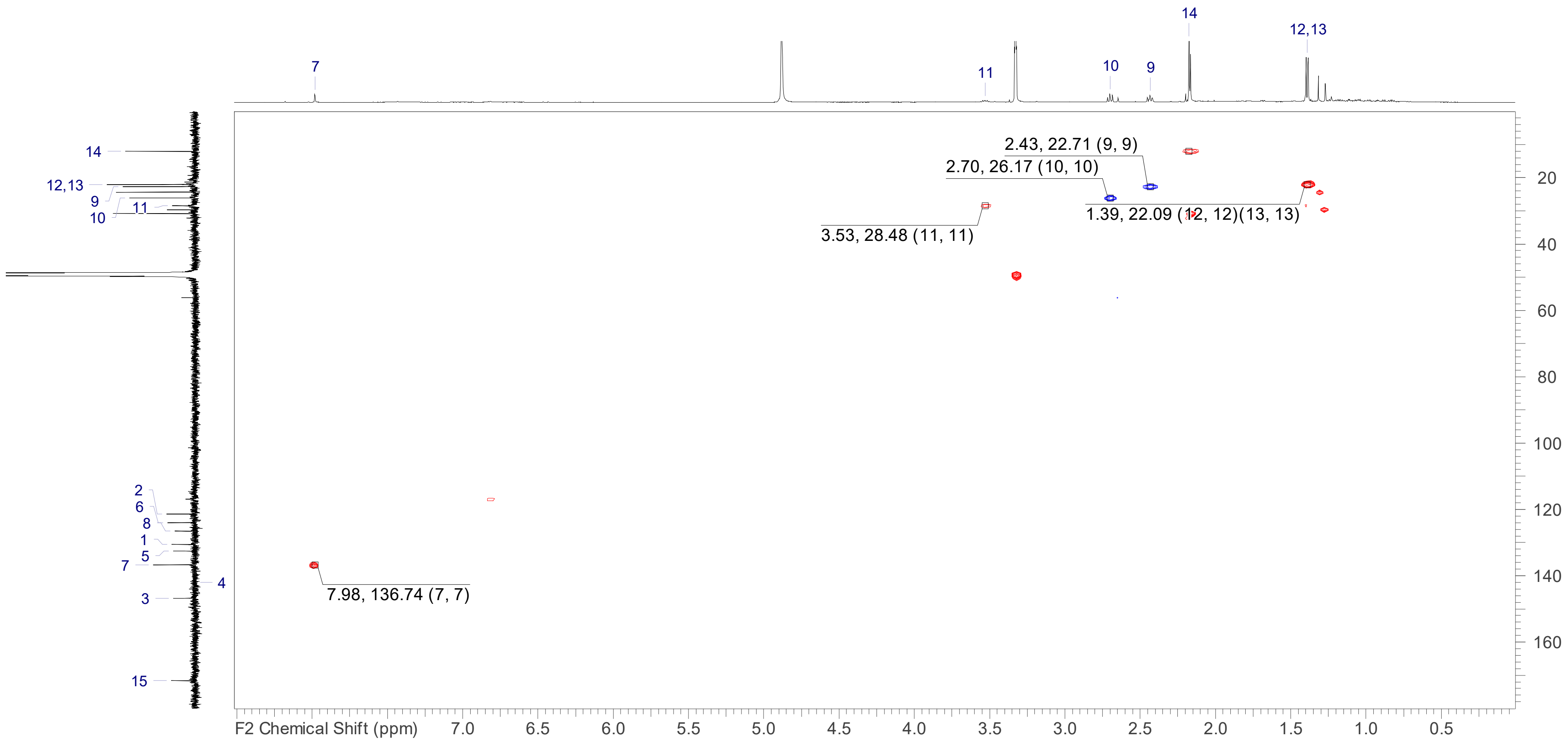

HMBC NMR spectrum (500 MHz, methanol-d_4) of heimiocalamene B (**8**).

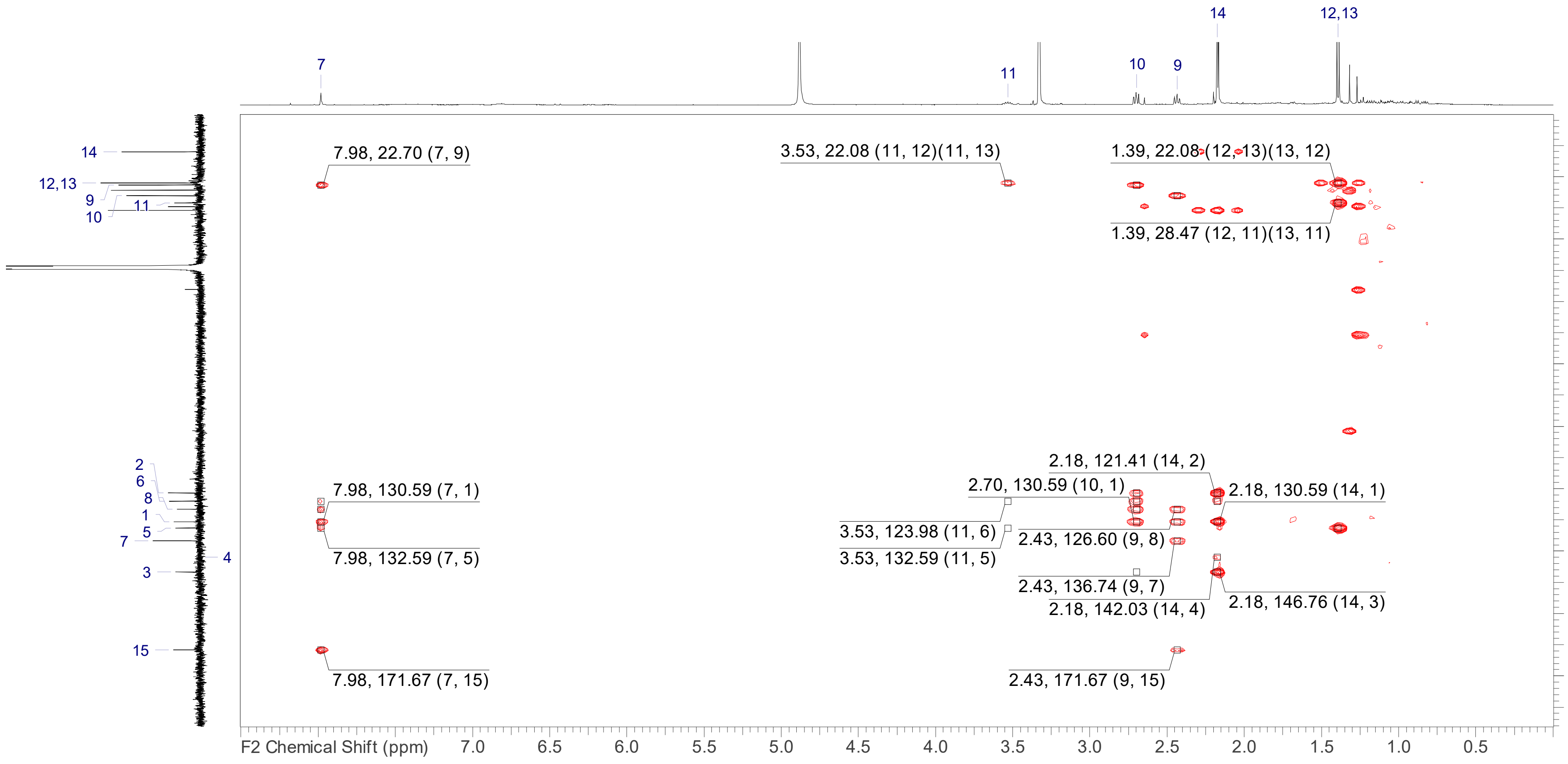

ROESY NMR spectrum (500 MHz, methanol-d_4) of heimiocalamene B (**8**).

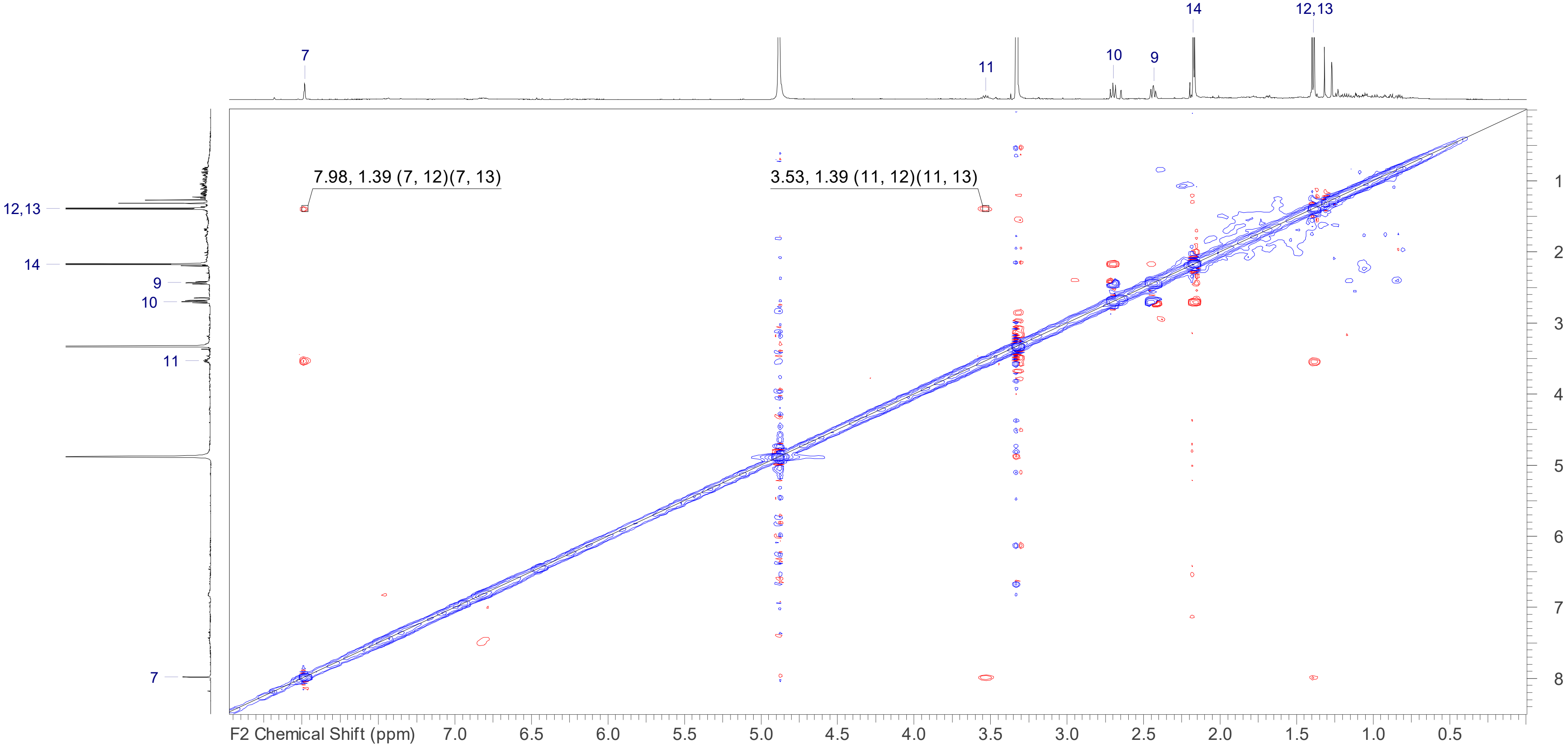

^{1}H NMR spectrum (700 MHz, DMSO-d_6) of heimiocalamene E (**11**).

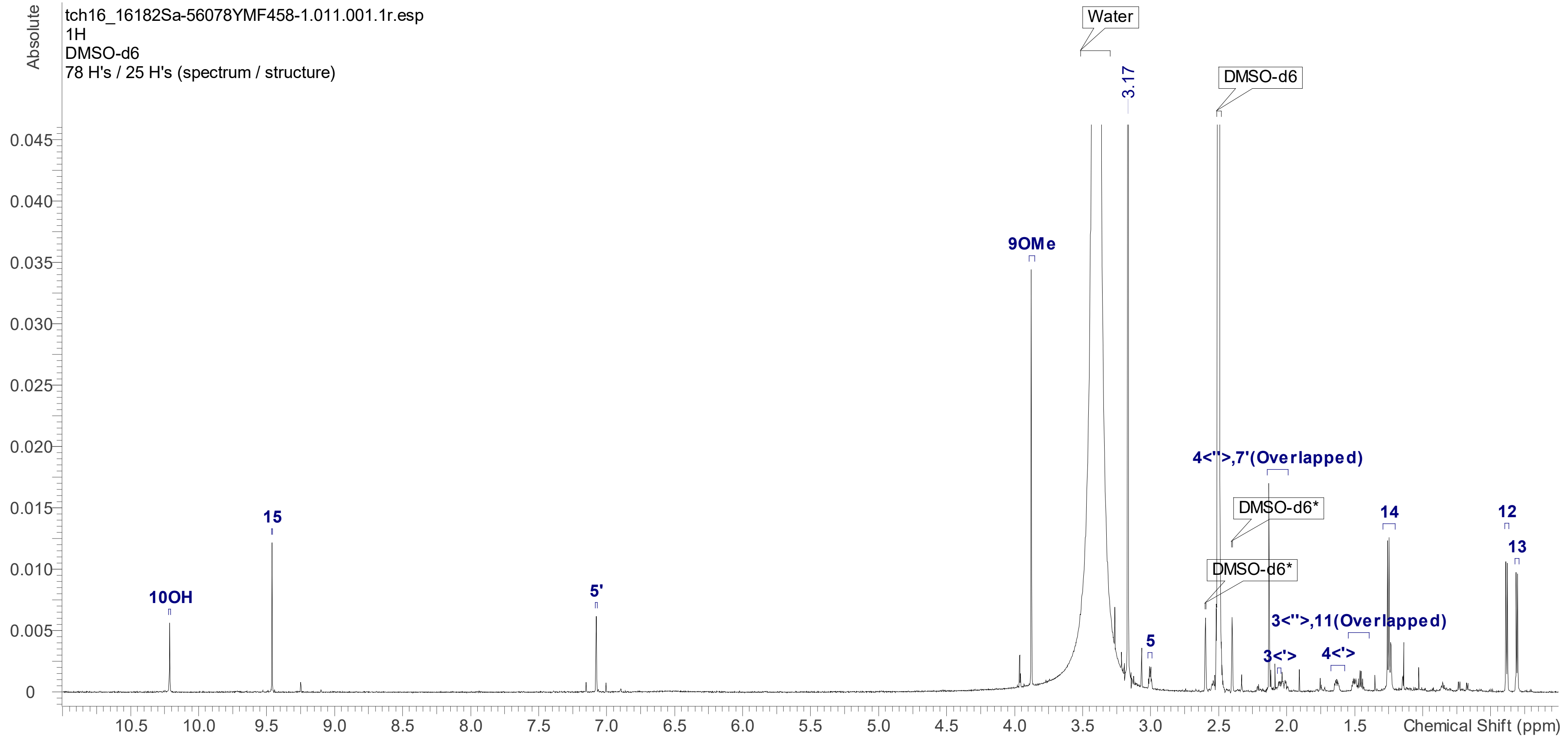

^{13}C NMR spectrum (175 MHz, DMSO-d_6) of heimiocalamene E (**11**).

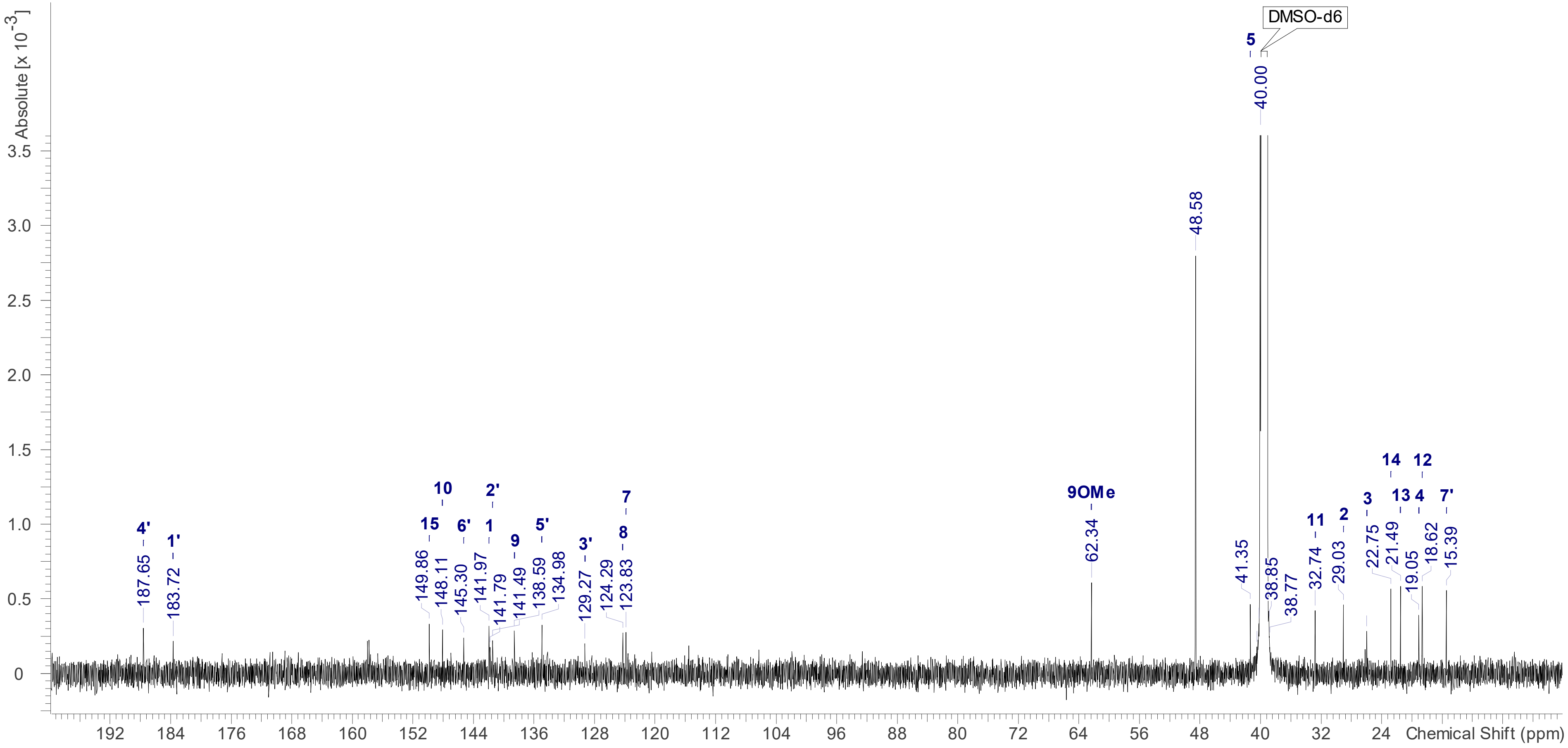

COSY NMR spectrum (700 MHz, DMSO-d_6) of heimiocalamene E (**11**).

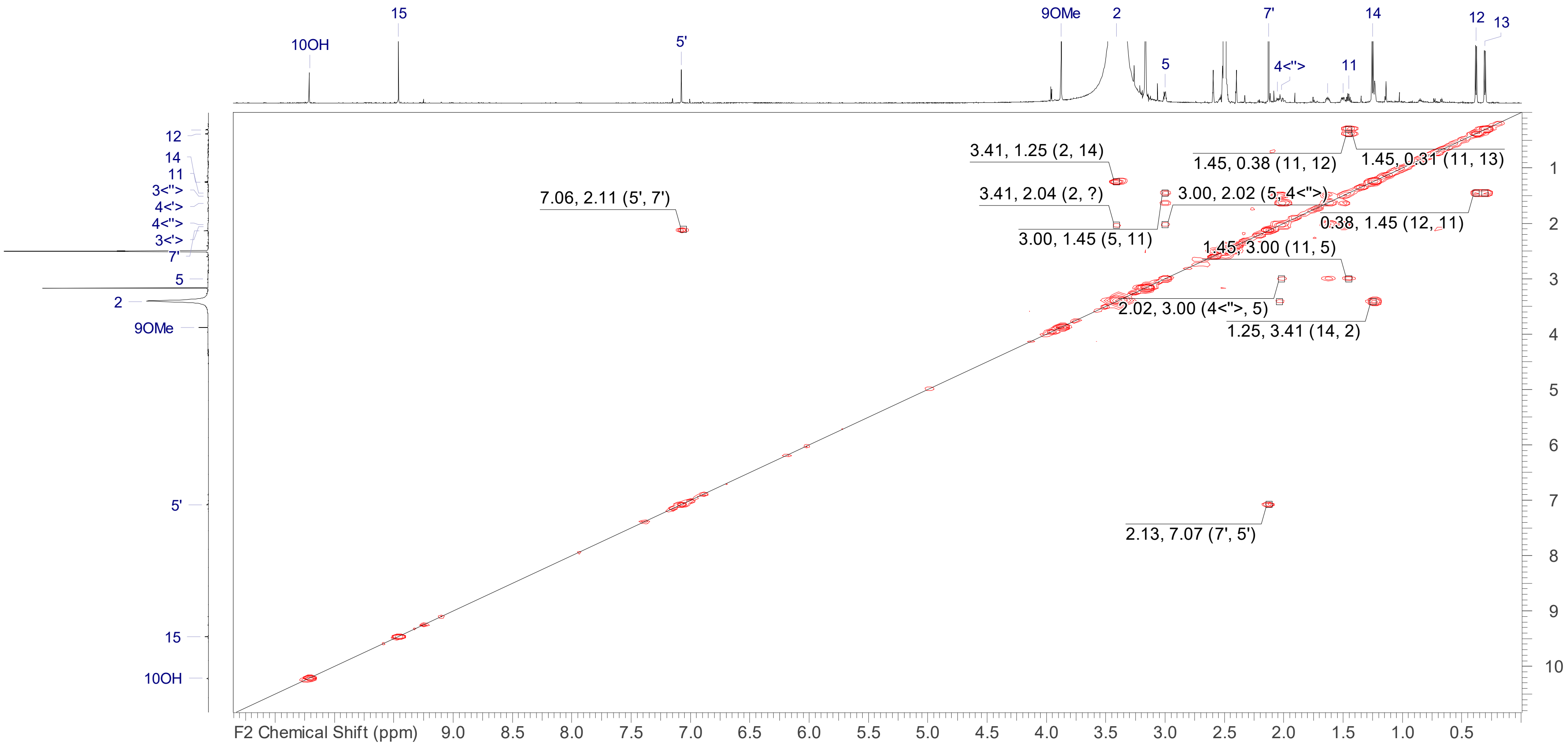

HSQC NMR spectrum (700 MHz, DMSO-d_6) of heimiocalamene E (**11**).

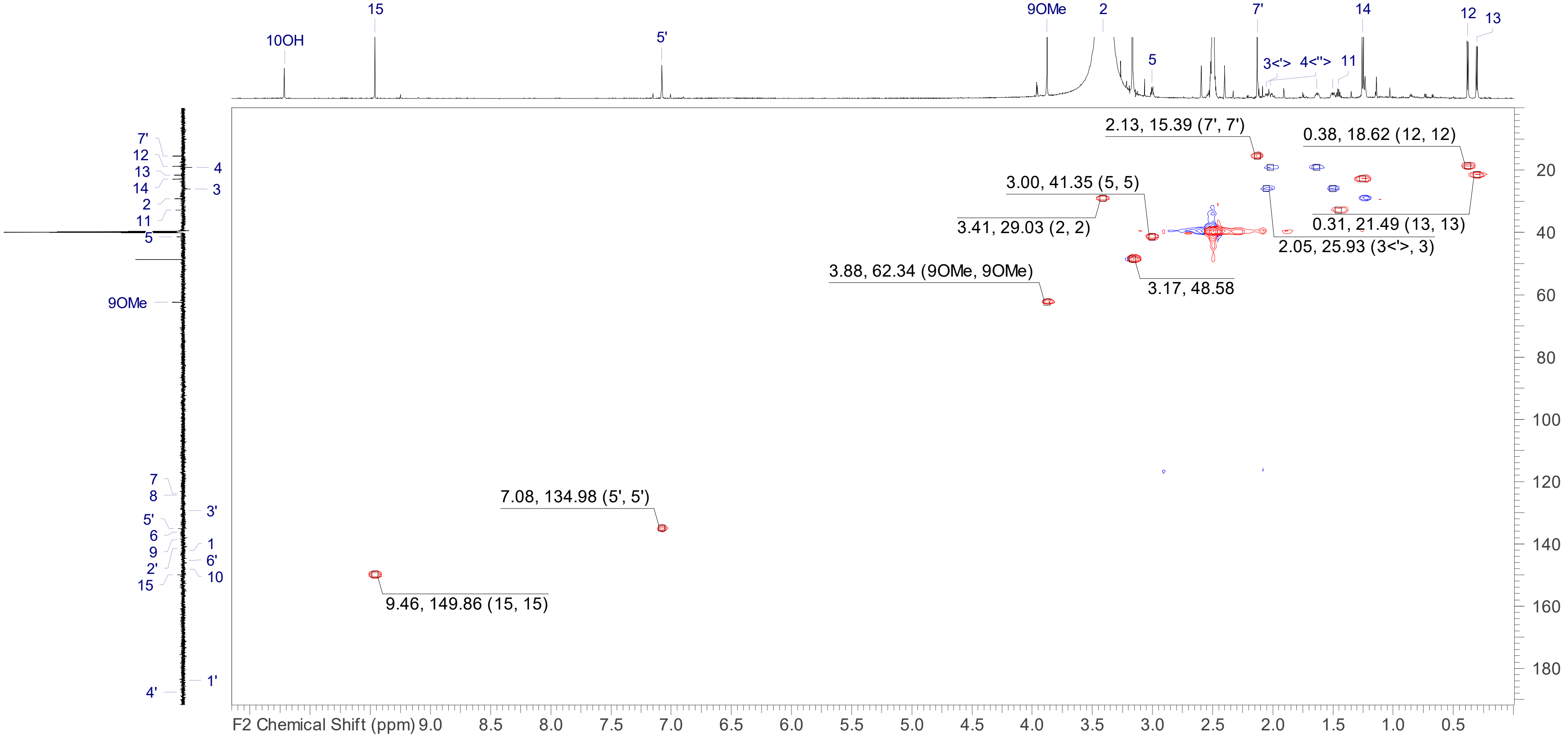

HMBC NMR spectrum (700 MHz, DMSO-d_6) of heimiocalamene E (**11**).

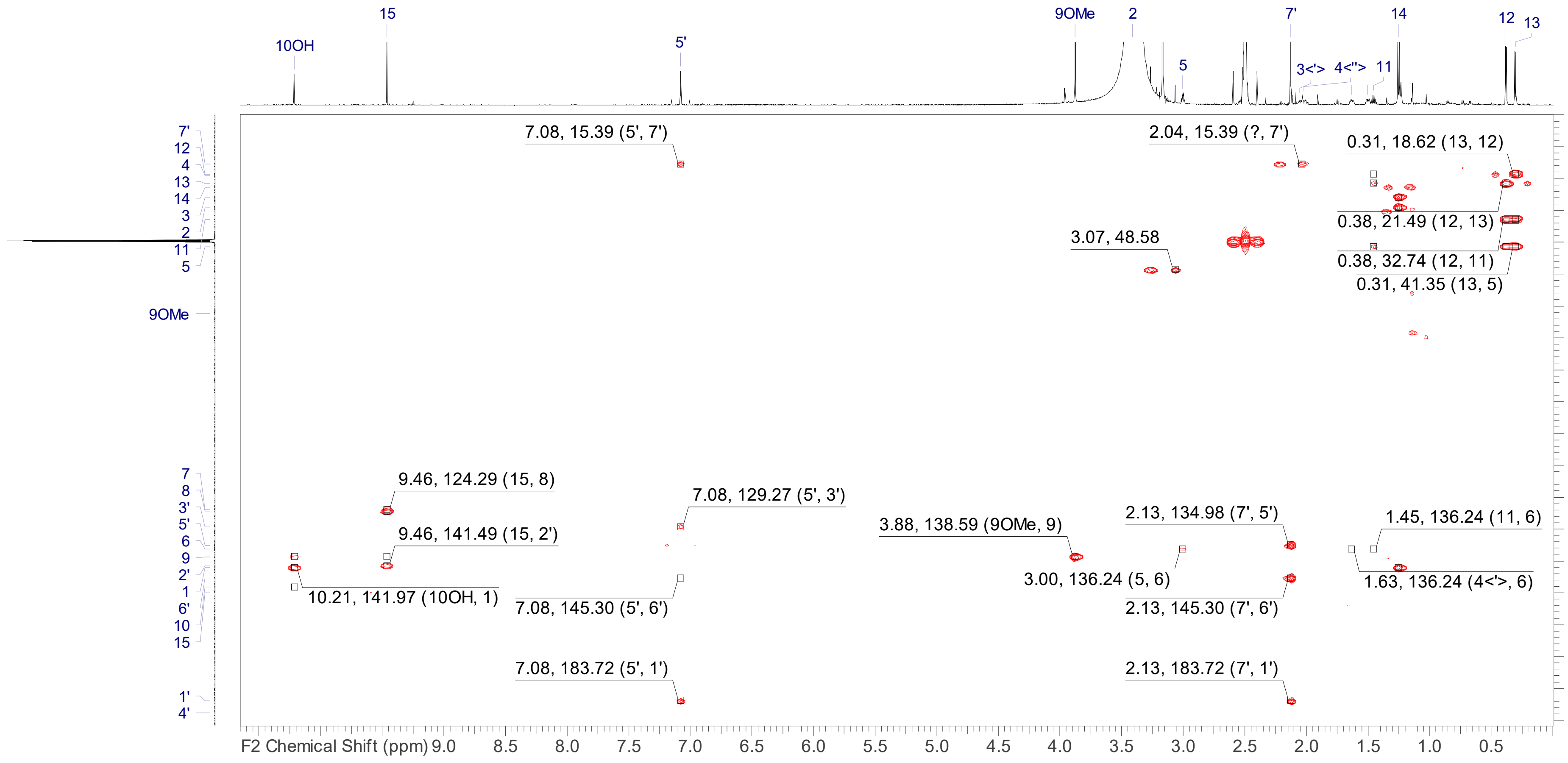

ROESY NMR spectrum (700 MHz, DMSO-d_6) of heimiocalamene E (**11**).

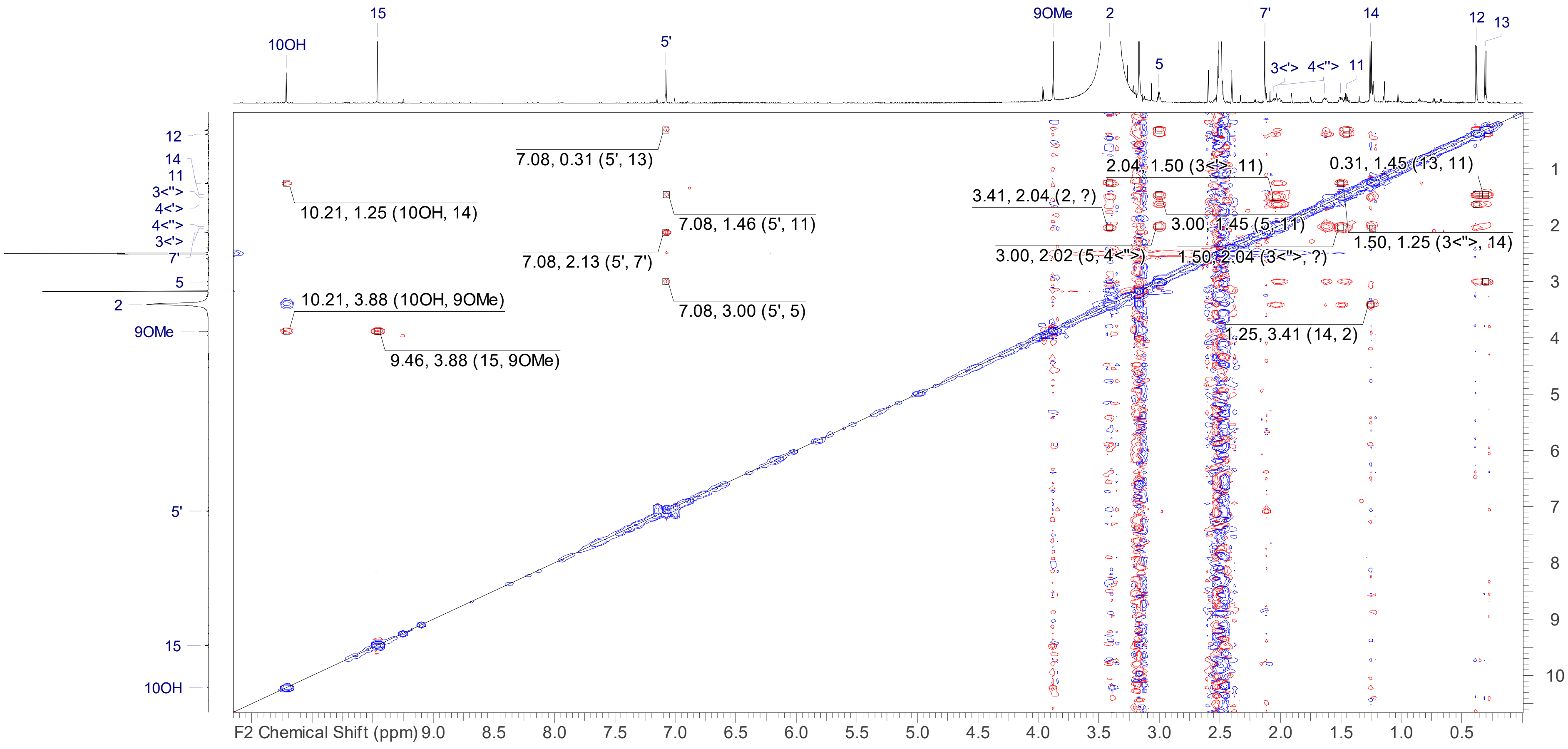

Supplement: Supplementary file 1 — np2c01015_si_001.pdf [file np2c01015_si_001.pdf]
